# Global stalled tropical cyclones in a changing climate

**DOI:** 10.1038/s41467-026-71320-3

**Published:** 2026-03-30

**Authors:** Zifeng Deng, Gabriele Villarini, Wenchang Yang, Gabriel A. Vecchi, Zhaoli Wang

**Affiliations:** 1 School of Civil Engineering and Transportation, State Key Laboratory of Subtropical Building and Urban Science, South China University of Technology, Guangzhou, China; 2 Department of Civil and Environmental Engineering, Princeton University, Princeton, NJ USA; 3 High Meadows Environmental Institute, Princeton University, Princeton, NJ USA; 4 Department of Geosciences, Princeton University, Princeton, NJ USA; 5 Pazhou Lab, Guangzhou, China

**Keywords:** Projection and prediction, Natural hazards

## Abstract

Tropical cyclone (TC) stalling refers to a storm wandering within a relatively small region. When TC stalling occurs, localized accumulated damage can increase substantially. However, the understanding of this special behavior globally, especially its response to climate warming, remains limited. Here, we provide a comprehensive global analysis of TC stalling and its response to climate warming, utilizing both observational data and climate model simulations. Our results reveal a distinct hemispheric asymmetry, showing that basins in the Southern Hemisphere are more prone to TC stalling than those in the Northern Hemisphere. Although a warming climate reduces the global probability of TC stalling occurrence, it significantly increases the daily rainfall by these storms, particularly over land and nearshore regions. Our analysis also indicates that, although the main drivers for the stalling vary in different basins, in general, they are mainly influenced by the steering wind vector (magnitude and direction) and TC location. Furthermore, changes in probability of TC stalling in climate warming are mainly affected by changes in the probability of TC exposure to a weak steering flow.

## Abstract (web summary)

Global analysis finds stalled tropical cyclones are more common in the Southern Hemisphere. Climate warming slightly reduces how often storms stall but increases rainfall from stalled storms, especially over land, heightening flood risk.

## Introduction

The motion of tropical cyclones (TCs) is a key factor in determining the location and extent of storm-related damages^1,2^. For a specific location, slow-moving TCs prolong the exposure to the storms, leading to greater accumulated damage. Thus, it is crucial to better understand how the motion of TCs may change under climate change.

TC stalling, which refers to a storm wandering within a relatively small region for a prolonged period due to its slow translation speed and/or large turning angle, is an important behavior of TC motion^3,4^. Hurricane Harvey (2017) exemplifies this behavior, with its re-curvature near Houston, Texas, where it remained for nearly three days, resulting in accumulated rainfall exceeding 1270 mm in 5 days^5,6^, more than 95% of the annual mean rainfall of 1317 mm per year over this region. This event caused unprecedented flooding and is one of the costliest natural disasters in U.S. history. Events like TC Ewiniar (2018), which caused record-breaking rainfall while stalling along the South China coast^7^, further highlight the role of stalling in amplifying the destructive potential of TCs.

Recent studies have focused on TC stalling in various cyclone-prone regions. TC stalling near the North American coast was found to have been increasing between 1944 and 2017, potentially associated with low-frequency natural variability^4^. Another study outlined the spatiotemporal climatology of stalled TCs across the North Atlantic (NA), revealing their patterns of seasonality and rapid intensification^8^. In parts of the Western North Pacific (WP), TC stalling has shifted poleward during the 1979–2020 period, with results suggesting that binary cyclone interactions may play a role in triggering these stalled events^3^. Despite these regional insights, a global perspective on TC stalling remains limited.

These observed changes in the historical record have been happening in a background characterized by human-induced warming. Recent studies indicate that climate change is expected to intensify different storm characteristics, including the average TC intensity, the TC rapid intensification, the proportion of strong TCs (1-min maximum sustained winds (MSW) of at least 113 kt), the average TC rainfall rates, and the frequency of TC induced heavy rainfall^9–15^. Additionally, while observational evidence remains inconclusive regarding the role of anthropogenic warming on the global TC frequency and mean translation speed^9,16–19^, numerical simulations indicate that climate change has reduced the global frequency of TCs and is projected to increase their future annual-mean translation speed^20–23^. Despite substantial progress in understanding the impacts of climate change on the aforementioned TC characteristics, it is still unclear how TC stalling responds to climate warming.

Here, we provide a global perspective on the climatology of TC stalling based on observational data and investigate how the frequency of TC stalling and its associated rainfall may change under climate change. Moreover, we examine the potential dynamical factors inducing TC stalling and investigate the primary drivers responsible for its variability in a warming climate. Our results reveal a distinct hemispheric asymmetry, showing that basins in the Southern Hemisphere are more prone to TC stalling than those in the Northern Hemisphere. Although a warming climate reduces the global probability of TC stalling occurrence, it significantly increases the daily rainfall by these storms, particularly over land and nearshore regions. Our analysis also indicates that, although the main drivers for the stalling vary in different basins, they are mainly influenced by the steering wind vector (magnitude and direction) and TC location. Furthermore, changes in probability of TC stalling in climate warming are mainly affected by changes in the probability of TC exposure to a weak steering flow.

## Results

### Global climatology of TC stalling

**Fig. 1:**
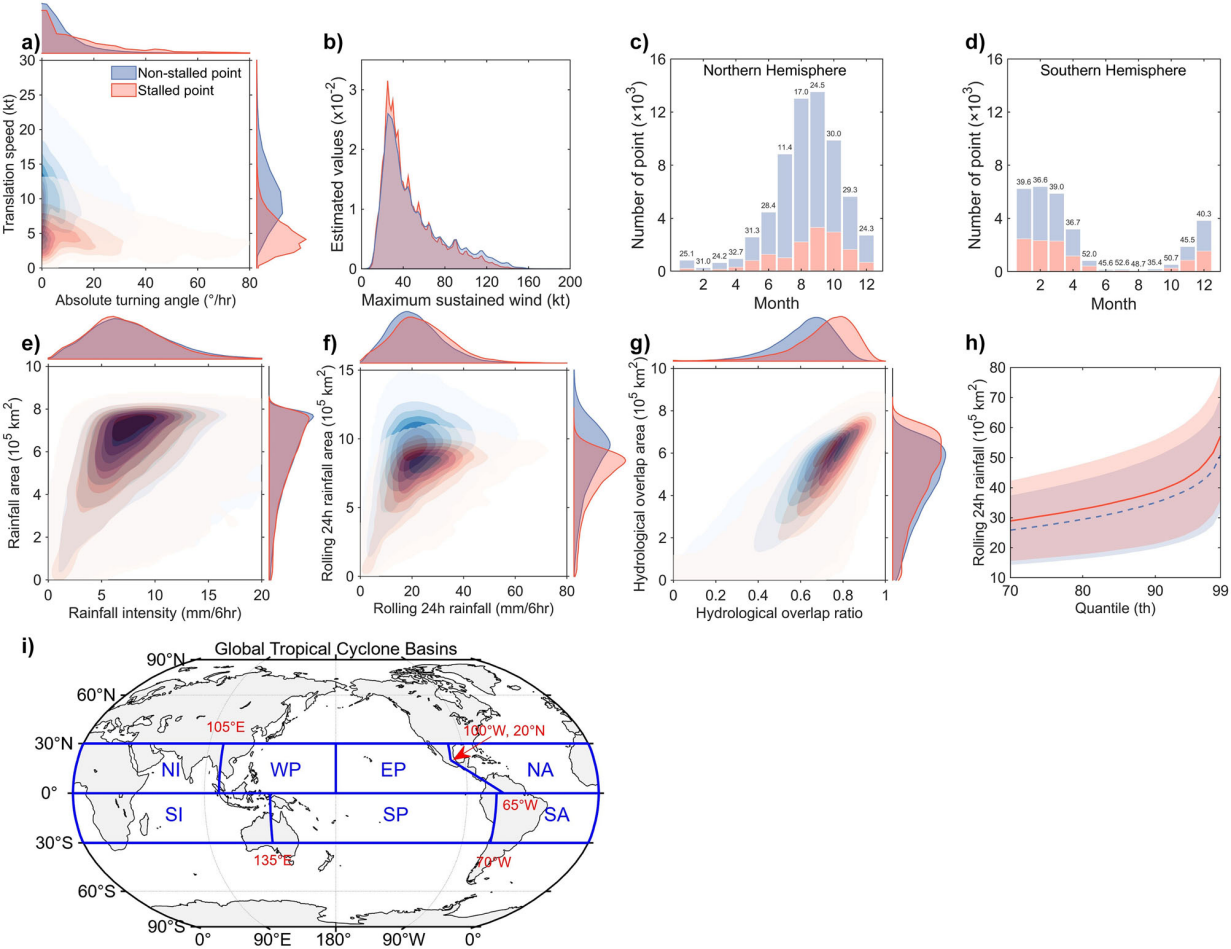
General characteristics of stalled and non-stalled track points of global tropical cyclone (TC). The two-dimensional kernel density estimations of (**a**) translation speed against absolute turning angle, (**e**) rainfall area against rainfall intensity at 6-hourly timescale, (**f**) rolling 24 h rainfall area against rolling 24 h rainfall, and (**g**) the overlap area against overlap ratio for the stalled and non-stalled TC track points (see Methods). The kernel density estimations of (**b**) maximum sustained wind (MSW) (kt). **c**, **d** Histograms of the frequency of the stalled and non-stalled TC track points in the seasonal cycle. The monthly ratios of stalled track points to total ones are shown above each bar, expressed as a percentage. Values of the areal mean rolling 24 h rainfall fields between the 70th and 99th percentiles are shown in (**h**), with the shading denoting the 95% confidence intervals of each percentile. Global TC basins are shown in panels **i**, with the latitude and/or longitude of key boundary points shown in red. The NI, WP, EP, NA, SI, SP, NH, SH, and GLO represent the North Indian, Western North Pacific, Eastern North Pacific, North Atlantic, South Indian, South Pacific, North Hemisphere, South Hemisphere, and Global basins, respectively.

Here, we defined the TC stalling as the behavior of a TC remaining within a 200-km radius for more than 48 h. During the 1982–2019 period, stalling occurs in ~28% of the tracked TC centers of circulation worldwide, resulting in a total of 1626 stalled segments; 44% of TCs experience stalling, and about 6% contain two or more stalling segments. Over land and nearshore regions (i.e., areas within 200 km of the coastline), stalling occurs in ~36% of TC track points, leading to 353 stalled TC segments (see “Methods”). Figure 1 shows the general characteristics of global stalled and non-stalled TC track points from 1982 to 2019. In terms of TC motion, stalled track points have lower translation speeds and greater turning angles compared to non-stalled points, features consistent with the definition of TC stalling. Specifically, the median translation speed of stalled track points was 8.2 km h^−1^, less than half of that of non-stalled track points (18.4 km h^−1^). The median turning angle of stalled track points was 12.1° h^−1^, more than double that of non-stalled track points (5.6° h^−1^). Regarding TC intensity, density estimates at high MSW for non-stalled track points are higher than those for stalled track points on a global scale, with this difference mainly occurring in the Northern Hemisphere (NH) (Supplementary Figs. 1–3). The ratio of TC stalling frequency to total TC activity (hereafter referred to as TC stalling ratio) of the Southern Hemisphere (SH) is 1.69 times that of the Northern Hemisphere (Fig. 1c, d and Supplementary Table 1), with the lowest TC stalling ratio observed in the NA basin and the highest in the South Pacific (SP) basin (Supplementary Figs. 1–3c).

Regarding TC rainfall, our results indicate that TC stalling generally produces more intense rainfall over a smaller area compared to non-stalled tracks (see the “TC rainfall” section in the “Methods”; Fig. 1f). Globally, on a rolling 24-h timescale, stalling leads to ~12% more accumulated rainfall within an area ~15% smaller than that of non-stalled tracks. However, the Eastern Pacific (EP) basin is an exception, where the accumulated rainfall from TC stalling is smaller than that from non-stalled storms. This can be attributed to the relatively lower sea surface temperature (SST) in the EP basin compared to that in other basins. Specifically, TC rainfall area is controlled primarily by its surrounding SST relative to the tropical mean SST (that is, the relative SST)^24^. This low SST in the EP basin results in small rainfall area of TCs and overlapping area (Supplementary Fig. 2f), thereby reducing the local rainfall accumulation induced by TC stalling. On the other hand, there is a smoother distribution of overlap ratio (see “Methods”) with a lower median for stalled points in the EP compared to the other basins, which may partially explain the rainfall characteristics in this region. Nonetheless, the extreme rolling 24-h rainfall values (i.e., above the 95th percentile of the rainfall distribution at a given location) of TC stalling are greater than those of non-stalled tracks in all basins, suggesting that TC stalling poses a greater hydrologic threat.

**Fig. 2:**
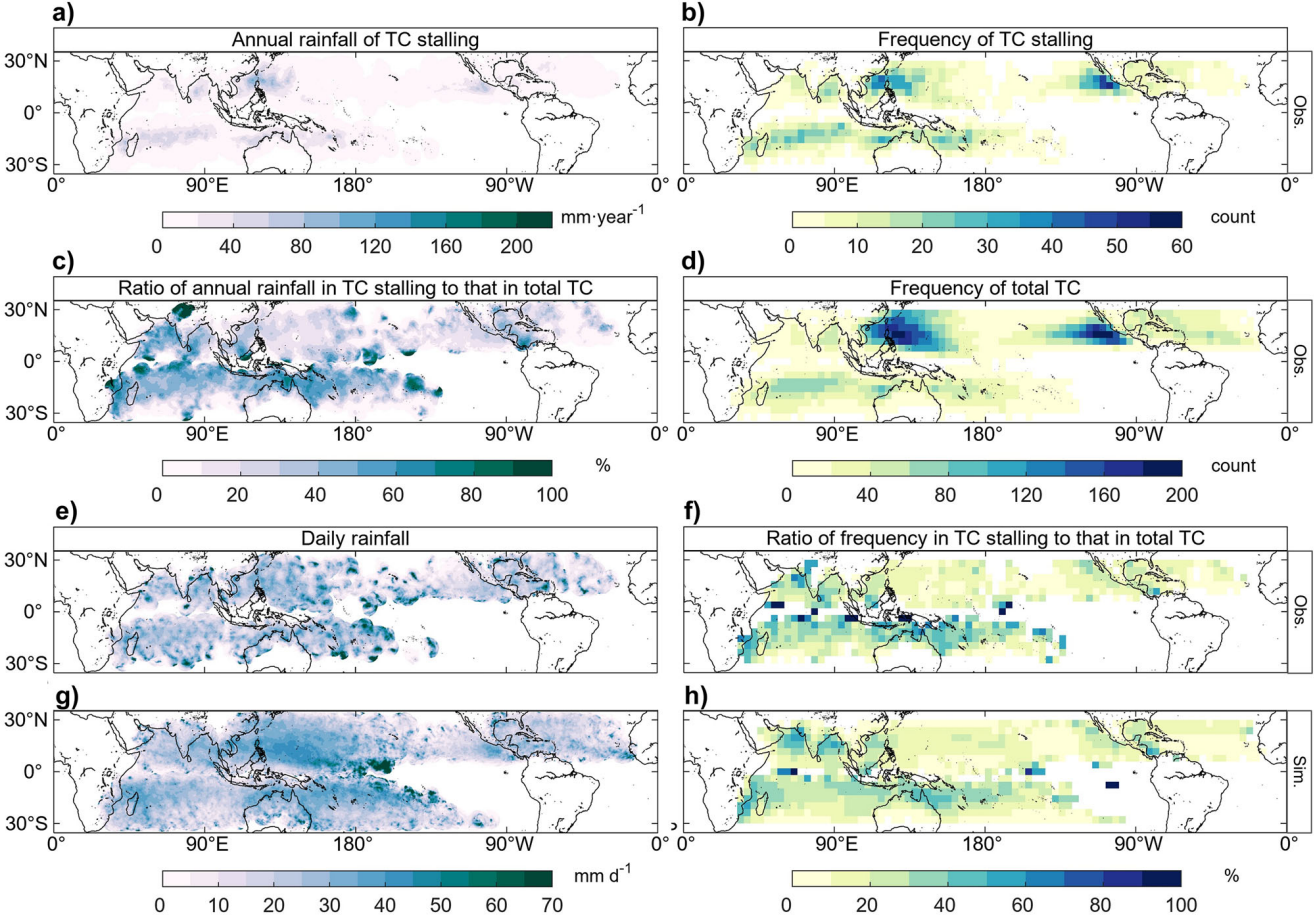
Spatial pattern of stalled and non-stalled tropical cyclone (TC) track points and corresponding rainfall. Spatial patterns of annual rainfall (**a**), frequency (**b**), and daily rainfall (**e**) of TC stalling, and the frequency of total TC (**d**), the ratio of annual rainfall (**c**) and frequency (**f**) in TC stalling to that in total TC (hereafter referred to as TC stalling ratio) are obtained from observations. Spatial patterns of daily rainfall (**g**) and ratio (**h**) of TC stalling are obtained from simulations. The results of daily rainfall are for the 2000–2019 period, while the other results are for the 1982–2019 period. To compare observed and simulated daily rainfall, the spatial resolution of the observed results (10 km) is resampled to that of the simulations (25 km). Frequency results are shown on grids with a spatial resolution of 4°.

Figure 2 shows the spatial pattern of the frequency and rainfall associated with TC stalling. Regions with high absolute frequency of TC stalling overlap with areas that experience large TC activity, so we focus on the TC stalling ratio. Although numerous TC stalling events occur in the South China Sea and Philippine Sea in the WP, as well as the EP off the coast of Mexico, the corresponding TC stalling ratios are only ~10% to 20%. In contrast, the average TC stalling ratio in the low-latitude regions of the SH (i.e., 4°S to 16°S) and the land and offshore regions of the Northern Indian (NI) exceed 35%, indicating that TC stalling is more likely to occur in these regions. Similar spatial patterns are obtained from the analyses of annual rainfall. However, due to a lack of coverage from geostationary satellites over an area centered on 70° east during the July 1983–June 1998 (except for April 1988–March 1989), there is an artifact in the observations over the NI during this period, commonly called the “Indian Ocean gap”^25^, and the results of this basin need to be interpreted with caution.

### Climate change impact on TC stalling

**Fig. 3:**
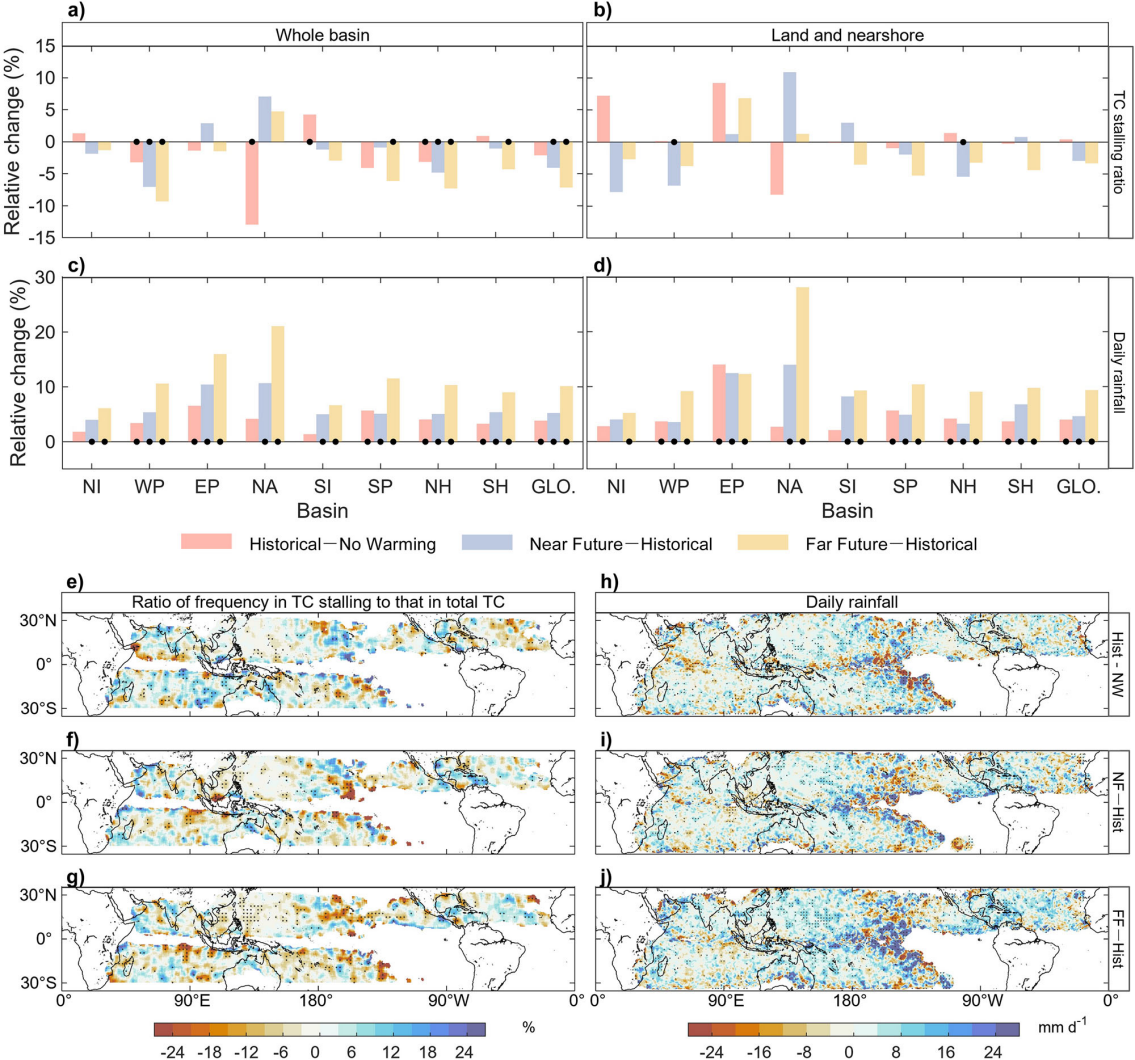
Changes in the ratio and daily rainfall of tropical cyclones (TCs) stalling under global warming. Relative changes in TC stalling ratios (**a**, **b**) and daily rainfall (**c**, **d**) between “Historical” scenario and “No Warming,” “Near Future,” and “Far Future” scenarios are calculated for the whole basin (**a**, **c**) and the land nearshore regions (**b**, **d**). Black dots in (**a**–**d**) indicate differences significant at the 5% level with two-tail *t*-tests. The NI, WP, EP, NA, SI, SP, NH, SH, and GLO represent the North Indian, Western North Pacific, Eastern North Pacific, North Atlantic, South Indian, South Pacific, North Hemisphere, South Hemisphere, and Global basins, respectively. Spatial changes of TC stalling ratio (**e**–**g**) and daily rainfall (**h**–**j**) between “Historical” scenario and “No Warming,” “Near Future,” and “Far Future” scenarios. In the results of TC stalling ratios, the ratios shown are based on the absolute frequency of TC stalling at least at the 25th percentile in the “Historical” scenario. To smooth the spatial pattern, the results for TC stalling ratios are shown on grids with a spatial resolution of 0.5°, while the results for each 0.5° × 0.5° grid are the result within a 4° × 4° area centered on that grid. “Hist” represents the “Historical” scenario, “NW” represents the “No Warming” scenario, “NF” represents the “Near Future” scenario, and “FF” represents the “Far Future” scenario (see “Method” for more details on the numerical experiments). Black dots in (**e**–**j**) indicate differences significant at the 5% level using bootstrap method.

Our high-resolution ensemble simulations reproduce the spatial and temporal patterns of the observed frequency and rainfall of TC stalling, providing confidence in exploring the impacts of climate warming on TC stalling (Fig. 2e–h and Supplementary Figs. 4–7) (see “Method” for more details on the validation). Therefore, we analyze the impact of climate change on TC stalling frequency and daily rainfall by comparing ensemble simulations under different scenarios (see Methods). Although climate change over the 20th century has led to a non-significant global decrease (−2.1%) in the TC stalling ratio, this decrease is projected to become more pronounced with increasing temperatures throughout the 21st century (−7.2% to −4.1%; Fig. 3a). On a basin-wide scale, climate change has already significantly reduced the TC stalling ratios in the WP (−3.2%) and NA basins (−13.0%), while it has increased the stalling ratio in the SI basin (~4.3%). Compared to the other basins, only the WP basin shows significant negative changes in the TC stalling ratio due to climate change over the 20th century and for future conditions. Except for the EP and NA basins, TC stalling ratios are projected to decrease in most basins, even though these changes are not significant. Over land and nearshore regions, global warming has not significantly altered TC stalling ratios either globally or within individual basins during the historical period but is projected to significantly reduce the TC stalling ratio in the WP basin between −3.7% and −6.8% (Fig. 3b). A previous study^4^ reports an increasing trend in near-coastal stalling events in the NA basin, different from our results; this difference is primarily attributable to differences in the spatial extent of the study areas: our analysis encompasses a broader domain covering all the land and nearshore regions in the NA basin, while their investigation focuses on the coastline of the American continent within the NA basin (see Fig. 2 in ref. ^4^). From a spatial perspective, climate change results in extensive, contiguous regions with decreased TC stalling ratios in the WP basin (Fig. 3e–j). In the South China Sea, Caribbean Sea, and Gulf of Mexico, the spatial patterns of changes in TC stalling ratio exhibit roughly similar characteristics between the “Near Future” and “Far Future” scenarios (see Method for more details on the numerical experiments). In the low-latitude SI basin, climate warming has increased TC stalling ratios in most of the region, whereas projections point to a change in the opposite direction, similar to what found in the NA basin, excluding the Caribbean Sea and Gulf of Mexico.

Regarding rainfall intensity, climate change has resulted in a significant increase in daily rainfall associated with TC stalling globally (3.8%), and these increases are further amplified with future warming across all basins (~5.2–10.1%) (Fig. 3c). Among them, only TC stalling-induced rainfall in the Pacific Ocean shows a significant positive response to climate change over the 20^th^ century, indicating that it is possible to already detect a global warming signal in the historical record for that basin. Spatially, there is a larger and more continuous area of positive change in the Pacific Ocean relative to the other basins (Fig. 3e–j), which is found in all warming scenarios. For land and nearshore regions, the simulations also indicate an increase in TC stalling-induced daily rainfall in the future (Fig. 3d). The positive change in the NA basin is the most dramatic in the future, approaching 30% in the “Far Future” scenario. Moreover, although projections indicate that the daily rainfall triggered by TC stalling is expected to be significantly less than that triggered by non-stalling storms globally, the projected increase in daily rainfall triggered by TC stalling is approximately 5.5–6.6% and 3.1–13.0% greater than that triggered by non-stalled tracks over land and nearshore regions of the EP and NA basins, respectively (Supplementary Fig. 8a–f). Moreover, TC stalling ratios over land and nearshore regions of these two basins are also projected to increase in the future. These results suggest a greater flood risk to North America caused by stalled than non-stalled tracks.

### Examination of the dynamical mechanisms for TC stalling

To understand dynamical factors that induce the TC stalling, we constructed basin-specific eXtreme Gradient Boosting (XGBoost) models^26^ incorporating both storm characteristics (i.e., longitude, latitude, maximum sustained wind speed (WindMax)) and storm-local environmental variables (see “Methods”). These variables include Steering Wind, the angle difference between TC motion and steering wind (Angle Diff), vertical wind shear (VWS), the area of high-altitude terrain (High Alt. Area), and the presence of coexisting TC interactions. Our XGBoost models have good out-of-sample prediction performances across all basins, with the Area Under Curve (AUC) values exceeding 0.83 and classification accuracy above 75% (Supplementary Table 2). The model in the NA basins achieved the highest performance, while that in the SI basin, though slightly lower, remained highly reliable. These robust metrics indicate that our XGBoost models have good prediction performance without overfitting, providing a reliable basis for interpreting dynamical drivers via SHapley Additive exPlanations (SHAP)^27^. The SHAP value (see Methods) is an interpretable artificial intelligence approach that represents the contribution from each feature to each individual prediction. A positive SHAP value indicates a contribution toward an increased stalling ratio and vice versa. The feature importance is defined by the mean value of absolute SHAP value, reflecting the sensitivity of stalling ratio to the dynamical drivers.

**Fig. 4:**
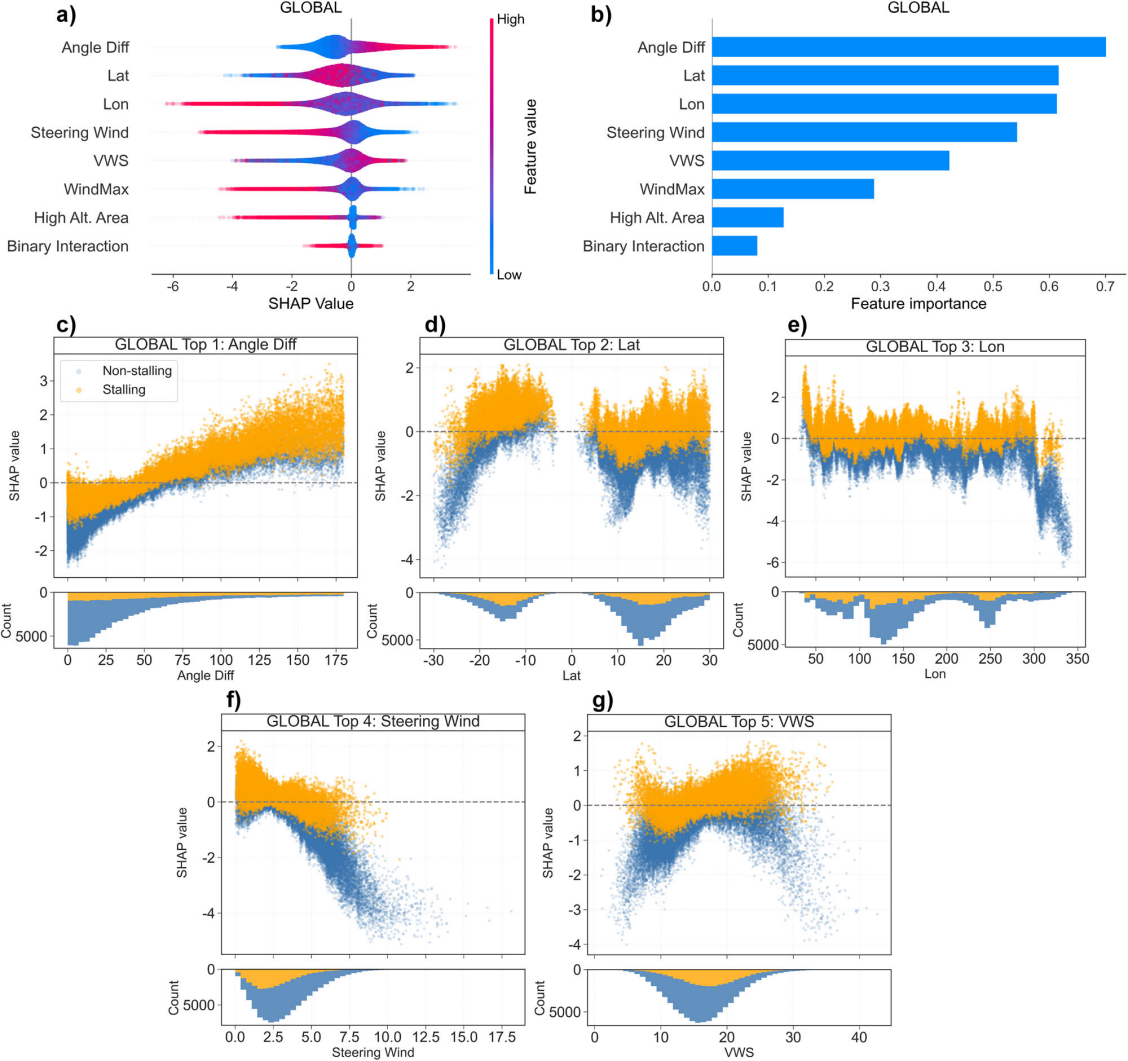
SHapley Additive exPlanations (SHAP) analysis from the global XGBoost model for tropical cyclone (TC) stalling. Beeswarm summary plot (**a**), feature importance (**b**), and relationship between the five most important features and stalling probability SHAP value (**c**–**g**) based on the global XGBoost model. Models are developed based on all the TC track points and storm-local environmental variables across the world.

Our SHAP analysis shows that the magnitude of the Steering Wind and the Angle Diff exhibit high feature importance across multiple basins (Fig. 4 and Supplementary Figs. 9–14). The probability of stalling scales positively with Angle Diff and negatively with steering wind speed. Physically, a large directional difference implies a misalignment between the TC motion and the steering flow, often inducing track deflection or deceleration^1^. Consequently, Angle Diff consistently ranks among the top two predictors in most basins (excluding the NA basin).

Geographic coordinates (Longitude and Latitude) emerge as critical features, including potential influences on TC from the Coriolis force, SST, and large-scale circulation regimes such as the trade winds, westerlies, and subtropical highs. The background steering wind varies significantly among different circulation regimes. For instance, in the SH, latitude exhibits a monotonic influence on stalling (Supplementary Figs. 13 and 14). This is because the steering wind in low-latitude regions is climatologically weak due to the South Pacific Convergence Zone and the weak Coriolis force^17,28^, and TCs are constrained by the subtropical high in the SH^29^, making them more likely to remain in low-latitude areas and thus increasing the stalling ratio. As for longitude, it only shows an evident monotonic influence on stalling in the NA basin (Supplementary Fig. 12). Specifically, the stalling ratio rises as TCs traverse westward toward the American continent. In the eastern and central Atlantic (extending from the African coast), TCs are typically embedded within the robust easterly trade winds along the southern periphery of the subtropical high, which facilitates rapid westward propagation and suppresses stalling. As storms reach the western NA and the vicinity of the American continent, the steering environment often deteriorates. This region is frequently characterized by the weakening of the subtropical high and the emergence of anomalous blocking highs, resulting in weak or opposite environmental steering flows that are conducive to stalling^23^.

With the exception of the SP basin, VWS generally has a negative relationship with stalling (Fig. 4). Dynamically, weak vertical shear fosters vertical coupling of the vortex. This structural coherence minimizes internal dynamical asymmetry, allowing the TC to be efficiently advected by the ambient steering flow. Conversely, strong shear induces vertical tilting and decoupling. The interaction between the displaced upper-level anticyclonic (clockwise in the Northern Hemisphere) circulation and the lower-level cyclonic (counterclockwise in the Northern Hemisphere) circulation can induce track deviations^1,30,31^, ranging from accelerated poleward drift to erratic looping or stalling. In the WP and NA basins, extreme VWS values exhibit a complex duality, being associated with both stalling and non-stalling events (Supplementary Figs. 10 and 12), likely related to the vertical wind profiles^32,33^. Notably, the SP basin presents a distinct anomaly, displaying an inverse relationship. Meanwhile, the steering wind at most TC track points in the SP basin is relatively low (falling below the baseline; Supplementary Fig. 14a). This implies that stalling in the SP basin is governed by a “quiescent environment” regime: stalling typically occurs when the entire vertical wind profile is weak, characterized by low wind speeds across all levels and small vertical differences in both speed and direction. Compared with the above-mentioned storm-local environmental variables, WindMax, High Alt. Area, and the coexisting TC interactions play subordinate roles across all basins.

### Climate change impact on TC environments

Building on the observational insights, we further investigate the impact of climate change on TC stalling by analyzing Steering Wind, Angle Diff, and VWS. To quantify the influence of these storm-local environmental variables under changing climate conditions, we solve the logit equation to obtain their critical threshold that corresponds to the probability of TC stalling in each basin in the “Historical” scenario. For example, the critical Steering Wind threshold in the WP basin is 3.5 m/s, which yields the mean probability of stalling during the simulated historical period (23.7%). The critical thresholds of storm-local environmental variables in each basin are shown in Supplementary Table 3. The response to VWS is non-monotonic: since both excessively high and low values are generally unfavorable for stalling, VWS is characterized by dual thresholds (VWS plots in Fig. 4 and Supplementary Figs. 9–14). We defined the specific ranges delimited by these thresholds as “stalling-inducing intervals.” The TC stalling ratio is higher when the values of environmental variables are in these stalling-inducing intervals (Supplementary Fig. 15).

We calculated the ratio of TC track points where their storm-local environmental variable values fall within the stalling-inducing intervals relative to the total track points (hereafter referred to as exposure ratio), focusing on the scenarios and basins exhibiting statistically significant changes in the TC stalling ratio. This exposure ratio reflects the probability of TC exposure to stalling-inducing environments. Table 1 shows that, in basins and scenarios with significant changes (indicated in bold), the signal of change in the stalling ratio aligns with that in the Steering Wind exposure ratio. This suggests that alterations in the probability of TC exposure to the stalling-inducing large-scale steering flow are the primary modulator of stalling variability in a changing climate. In the WP basin, although the Angle Diff exposure ratio exhibits a positive change (conducive to stalling) across the “No Warming,” “Near Future,” and “Far Future” scenarios, the significant and more pronounced reduction in Steering Wind exposure ratio and VWS exposure ratio dominates the signal. On the other hand, the climatological differences in Steering Wind between scenarios also suggest that the northward winds intensify across most parts of this basin, reducing TCs’ time in low-latitude areas under warming climate (Supplementary Fig. 16). The mean TC translation speed is higher in high-latitude areas than in the low-latitude ones^16^. For the NA and SI basins in the “No Warming” scenario, all three ratios of environmental variables showed consistent changes with the stalling ratios. Conversely, the SP basin under the “Far Future” scenario presents a more complex situation involving competing signals. Although both Angle Diff exposure ratio and VWS exposure ratio show positive changes, their impact is limited due to the insignificant change of the Angle Diff exposure ratio and the low feature importance of VWS in this basin (as determined by SHAP; Supplementary Fig. 14b). Consequently, the reduction in stalling ratio in this basin is likely driven by the significantly reduced probability of TC exposure to weak steering winds.

**Table 1:** Changes in the tropical cyclone (TC) environment for each basin under global warming

Basin	Variable	Relative change (%)		
		Hist - NW	NF - Hist	FF - Hist
NI	Angle Diff	3.73	3.10*	3.87
	Steering Wind	−3.23	3.90	4.28
	VWS	−7.88*	0.53	0.30
WP	Angle Diff	1.99*	**0.47**	**0.69**
	Steering Wind	−1.24*	−1.78*	−4.16*
	VWS	−4.73*	−6.36*	−10.37*
EP	Angle Diff	1.00	5.99*	7.87*
	Steering Wind	1.85	9.08*	8.70*
	VWS	−7.18*	−1.87	−3.67
NA	Angle Diff	−**2.01**	4.35	5.73*
	Steering Wind	−**1.91**	2.38	1.45
	VWS	−**0.03**	−6.05*	−6.30*
SI	Angle Diff	5.32*	−0.24	1.16
	Steering Wind	1.89*	−0.21	0.43
	VWS	**0.18**	−1.05	0.78*
SP	Angle Diff	−2.11*	3.54*	**1.30**
	Steering Wind	−0.78	−0.26	−2.34*
	VWS	5.49*	8.86*	12.26*

Values represent the relative changes in the ratio of TC track points where their storm-local environmental variable values fall within the stalling-inducing intervals relative to the total track points, comparing “No Warming,” “Near Future,” and “Far Future” scenarios to the “Historical” baseline. Asterisks (*) denote significant differences in relative changes at the 5% level using bootstrap method. Bold values indicate basins where the change in the overall TC stalling ratio is statistically significant (Fig. 3a).

**Fig. 5:**
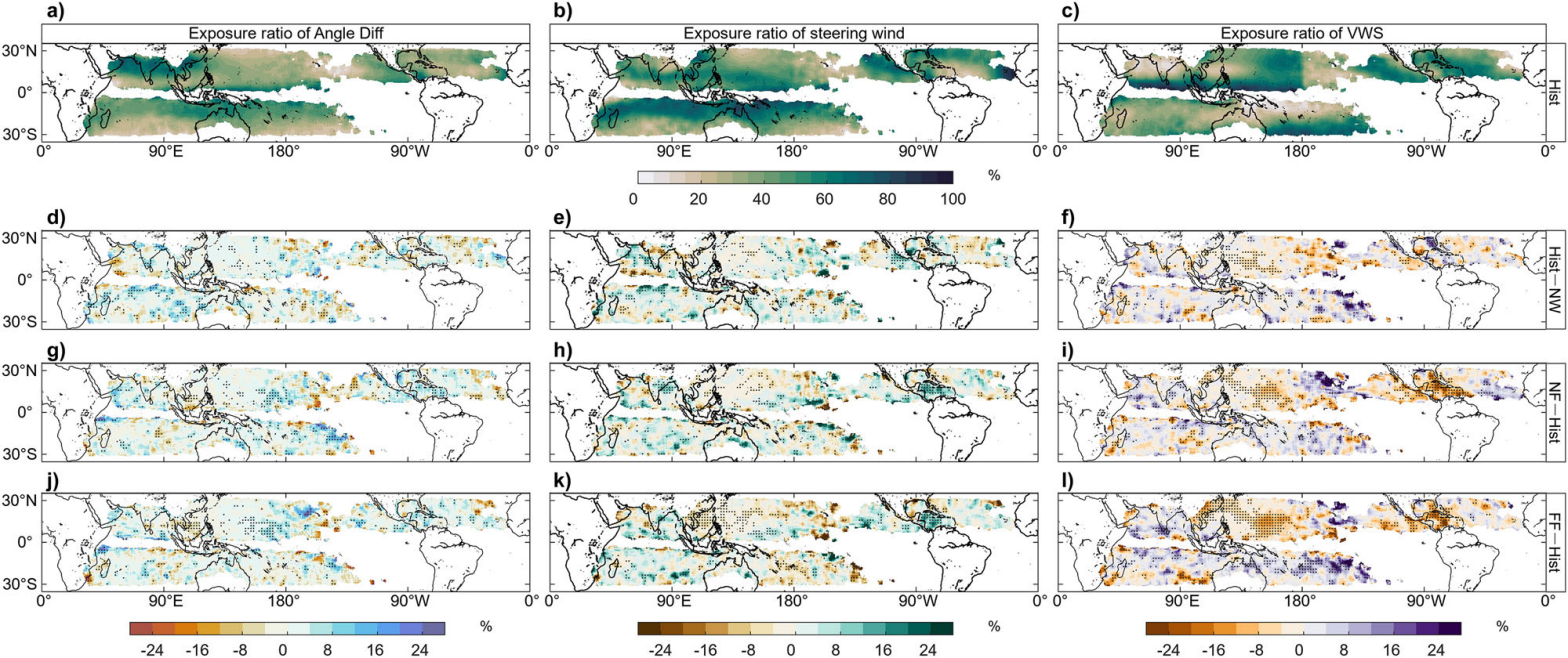
Spatial changes in the tropical cyclone (TC) environment for each basin under global warming. Spatial distributions (**a**–**c**) and their changes in the ratio of TC track points where their storm-local environmental variable values fall within the stalling-inducing intervals relative to the total track points, comparing “No Warming” (**d**–**f**), “Near Future” (**g**–**i**), and “Far Future” (**j**–**l**) scenarios to the “Historical” baseline. The results shown are based on the absolute frequency of TC stalling at least at the 25th percentile in the “Historical” scenario. To smooth the spatial pattern, the results are shown on grids with a spatial resolution of 0.5°, while the results for each 0.5° × 0.5° grid are the result within a 4° × 4° area centered on that grid. Black dots indicate significant differences at the 5% level using bootstrap method. In (**c**), the evident line (180°E) between the WP basin and the EP basin is caused by their different thresholds. “Hist” represents the “Historical” scenario, “NW” represents the “No Warming” scenario, “NF” represents the “Near Future” scenario, and “FF” represents the “Far Future” scenario.

The probability of exposure to stalling-conducive environments is also linked to the spatial distribution of TCs. In the SH, the analysis of the spatial changes of track points (Supplementary Figs. 17 and 18) and the spatial distribution of environmental variables (Fig. 5a–c) shows that the increased stalling ratio in the SI basin (“Historical” relative to “No Warming” scenarios) is associated with a higher frequency of TCs occurring in low-latitude regions (0–20°S). Conversely, the SP basin exhibits a decline in the stalling ratio under the “Far Future” scenario, which may be due to a reduced density of TC tracks in the low latitudes.

## Discussion

Previous studies on TC stalling have mainly focused on regional scales^3,4,8,23^, while our study takes a global perspective, providing an important reference for theories of TC climatology. We found that TCs in low-latitude regions of the SH are more likely to stall. Typically, stalling implies a risk of self-induced cooling (upwelling) and subsequent decay^34–36^. TCs that stall in higher latitudes (where waters are cooler and shear is higher) tend to dissipate rapidly and may not become a stalling event. However, the low-latitude regions of the Southern Hemisphere possess a deep oceanic mixed layer and high heat content^37,38^, which may act as a “thermal buffer,” sustaining TCs even when they wander. This suggests that the geography of stalling is not just a dynamical consequence, but also a thermodynamic survival function. This is why longitude and latitude have relatively high feature importance in the SI and SP basins. Future work is needed to further analyze the influence of the thermodynamic environment and the interior of TC on stalling.

Previous studies showed that TC stalling in the WP basin may be linked to coexisting cyclone interactions, in which a larger TC can change or weaken the steering flow of a smaller nearby TC^3,39^. However, our SHAP analysis indicates that the role of binary TC interactions is relatively limited and exhibits significant duality (Fig. 4 and Supplementary Figs. 9–14). This is likely because the dynamical perturbations induced by binary TC interactions are implicitly captured by the Steering Wind and Angle Diff in our model, thereby masking the explicit binary signal. On the other hand, the influence of binary TC interactions is basin specific. Globally, the probability of coexisting TCs at stalled track points is about 1.9–2.2% higher than at non-stalled track points (Supplementary Table 4). At the basin scale, the NI, SI, and WP basins all show a higher probability of coexisting TCs within different detection radii at stalled track points compared to non-stalled ones. However, results in the NA basin show the opposite changes, and in the EP basin and SP basin, different detection radii yield results in opposite signs. Although the binary cyclone effect involves physical mechanisms that influence TC movement^3,39^, and TC stalling is more strongly associated with this effect than non-stalling (Supplementary Table 4), the binary TC effect cannot be considered the main cause of TC stalling. These findings highlight the importance of understanding TC stalling from a global perspective.

Furthermore, even when the analysis is restricted exclusively to land and nearshore track points, the influence of high-altitude terrain remains limited across most basins, with the notable exception of the NI basin (Supplementary Fig. 19a–d), in the land and nearshore of the NI basin, High Alt. Area has a positive relationship with stalling. By isolating track points characterized by high topographic exposure (High Alt. Area > mean) and strongly negative contributions (SHAP value <−2; Supplementary Fig. 19e), we found that these track points may be governed by channeling effects^40,41^ (e.g., points in the Gulf of Aden and Gulf of Oman) or orographically induced deflection or jumps^40,42^ (e.g., points near the Arakan Yoma and on the south of the Himalayas).

Our analyses reveal the dynamical mechanisms driving TC stalling across three spatial scales. First, the large-scale synoptic environment establishes the macroscopic background for TC stalling. The high feature importance of geographic coordinates (longitude and latitude) (Fig. 4b) reflects that large-scale circulation systems provide a critical background field for the occurrence of stalling. These large-scale circulation systems shape the spatial climatological characteristics of steering wind intensity and direction, thereby determining the geographic distribution of stalled events^43,44^. Second, embedded within this macroscopic background, the storm-local environment exerts direct kinematic forcing on the TC. This is manifested in SHAP analyses by the high feature importance of Steering Wind and Angle Diff, with at least one of the two ranking among the top two predictors (Fig. 4b and Supplementary Figs. 9–14b). A weak steering wind and a large directional difference between the TC motion and the steering flow would force the TC to decelerate or deflect^1^. Third, the internal vortex structure acts as a mechanical receptor to external forcing, playing a modulating role during TC movement. When the vortex structure maintains vertical coherence, the TC responds efficiently to external forcing; in such cases, whether stalling occurs primarily depends on the external environmental conditions (e.g., stalling TCs in the SP basin; see Supplementary Fig. 14a). Conversely, strong VWS is prone to causing vortex tilting and dynamical interactions between the upper and lower levels, thereby inducing irregular track deflections or accelerated poleward drift^30,31^. The dynamical mechanisms across these three scales interact progressively, collectively driving the occurrence of TC stalling.

Climate change has profoundly altered TC behaviors^45,46^, but to date, there is a lack of research on the response of TC stalling to climate change. Using high-resolution ensemble simulations, we have provided a global assessment of the influence of climate change on TC stalling, with a focus on their frequency and rainfall. Our simulations show that the ratio of TC stalling is declining in most basins under climate change, which offers a subtle counterpoint to recent observational and simulated studies suggesting a general slowdown in TC mean translation speeds^2,16^. It is worth noting that TC stalling is a special behavior that differs from the widely discussed mean translation speed^2,22,47^. The latter is a general statistic of TCs and tends to be dominated by non-stalled track points, as it accounts for more than 70% of the total track points. In a warming climate, the decrease in stalling ratio is not contradictory to the decrease in the mean translation speed of TCs.

Our results also show that alterations in the probability of TC exposure to the stalling-inducing large-scale steering flow are the primary modulator of stalling variability in a warming climate. Although the SHAP analysis indicates that the directional misalignment between the steering wind and TC movements contributes more to stalling than the steering wind speed, the climate analysis results show that the role of this factor on the stalling changes caused by warming is less significant than that of the strength of environmental flow. This is particularly evident in the WP and SP basins. In both regions, climate change induces competing effects between signals of Angle Diff and Steering Wind. The simulated decrease in stalling ratios in these basins demonstrates that, in a warming climate, steering wind speed acts as a first-order constraint, overriding the stalling potential induced by directional misalignment. This finding simplifies the complexity of predicting future stalling storms: tracking the spatial extent of weak steering wind regimes (the stalling-inducing intervals) may be a more robust proxy than analyzing complex patterns of environmental flow.

Uncertainties remain in the future projections of TC frequency and motion. Although most climate models have projected decreases, or at least no change, in global TC frequency^48,49^, some models point to a future increase^50,51^. Regarding TC motion, some studies projected that future warming can lead to a robust slowing of TC motion, particularly in the mid-latitudes^2^, while others project that global TC translation speed would increase due to an increase in the relative frequency of TCs in the extratropics where the TC translation speed is much larger^22^. These previous studies could potentially lead to different results in the future change in the TC stalling. Future studies are needed to examine the variability across different climate models and multiple scenarios in TC stalling simulations. Given that atmosphere-ocean interactions modulate TC activity^52,53^, atmospheric-ocean coupled models with higher temporal and spatial resolution are needed to further investigate the physical mechanisms by which climate warming influences TC stalling behavior.

Compared to changes in TC stalling frequency, the simulated increase in daily rainfall is robust and significant, suggesting that climate change will further exacerbate the hydrological threat posed by stalling storms. North America, in particular, is expected to face greater hydrologic impacts by stalled compared to non-stalled storms. Future projections point to an intensification of TCs under climate change with high confidence^49,51,54^. Thus, climate change is likely to further amplify the destructive potential of TC stalling. The cumulative effects of hazard are more pronounced in areas affected by stalled track points than in areas affected by non-stalled track points, and thus TC stalling should be of greater concern. Our study highlights the importance of a deep understanding of the TC special behaviors being altered by anthropogenic factors.

## Methods

### Data

The observed TC tracks are obtained from version 4 of International Best Track Archive for Climate Stewardship (IBTrACS)^55,56^, which merges recent and historical TC data from multiple agencies to create a unified, publicly available, best-track dataset. We use records from the following agencies: National Hurricane Center (NHC), Joint Typhoon Warning Center (JTWC), and Central Pacific Hurricane Center (CPHC). To compare observed TC tracks with simulated TC tracks, we delete records with missing locations, those not at 00:00, 06:00, 12:00, or 18:00 UTC.

The observed TC rainfall is obtained from the Multi-Source Weighted-Ensemble Precipitation (MSWEP) V2.8^57^. This dataset merges rain gauge, satellite, and reanalysis data to obtain a rainfall estimate dataset with a 0.1° latitude × 0.1° longitude spatial resolution and a 3-h temporal resolution. We use the gauge-corrected historical version of MSWEP data, available from 1979.

To investigate the possible dynamical mechanisms of TC stalling, observed environmental variables were derived from the European Centre for Medium-Range Weather Forecasts (ECMWF) Reanalysis v5 (ERA5) with a spectral resolution of T639 (nominally 31 km)^58^. The main variables used in this study include 6-hourly horizontal winds at the 850 hPa and 250 hPa isobaric levels. Although 200 hPa is conventionally used to represent upper-level flows in TC analysis^59^, we selected the 250 hPa level to ensure vertical consistency and comparability with the output levels of the high-resolution ensemble climate simulations used in this study. Topographic features were characterized using the Global Multi-resolution Terrain Elevation Data 2010 (GMTED2010) at a spatial resolution of 30 arc-seconds^60^.

The period of study for this research is 1982–2019. Compared to the pre-satellite period, it is considerably less likely that any TC would be undetected in the post-geostationary satellite era^22^. Seven global TC basins covered in this study are defined in Fig. 1i.

### Model and numerical experiments

Large-ensemble simulations were conducted with AM2.5C360, an atmospheric general circulation model by the Geophysical Fluid Dynamics Laboratory (GFDL)^61^. AM2.5C360 has a horizontal resolution of about 25 km and is able to simulate many aspects of the observed TC variability over the past few decades during which reliable observations are available^62^. The AM2.5-C360 model generates realistic TC annual cycles and response to El Niño-Southern Oscillation^61,63^. We track the TCs in the climate model outputs using an algorithm that identifies features in the climate model output that satisfy several criteria resembling a real-world TC. The algorithm searches six-hourly output of the surface pressure and wind speed, vorticity at 850 hPa, and mid-level temperature fields to identify warm core cyclones^61,64^.

In the simulations for the historical scenario, we used the Atmospheric Model Intercomparison Project (AMIP)-type ensemble simulations (ten ensemble members). The “Historical” scenario representing the current climate is forced by SST from the Hadley Centre Sea Ice and Sea Surface Temperature dataset version 1^65^ (HadISST1) after groupwise bucket SST bias correction^43^. All the ensemble members are forced by the same historical SST and differ only in initial conditions. The “No Warming” scenario representing the absence of climate warming effects is simulated with five ensemble members. In this scenario, the atmospheric model is the same as that of the historical scenario except that the radiative forcings are fixed at the year 1880 level and the specified SSTs are modified so that the 15-year lowpass of global mean monthly SST anomalies (relative to the 1871–1890 monthly climatology) are subtracted at each ocean pixel. Simulations representing the future climate under the Representative Concentration Pathway 4.5 scenario are divided into two periods: the “Near Future” scenario from 2022 to 2059, and the “Far Future” scenario from 2062 to 2099. The future climate scenario is simulated with six ensemble members. In this scenario, the atmospheric model is forced by radiative forcings from the RCP4.5 scenario^66^. The SSTs are from the RCP4.5 scenario experiment using the GFDL-CM2.5-FLOR coupled climate model and bias-corrected by comparing monthly climatology with the HadISST1 observational data over the period of 2001–2020. Half of the six ensemble members of future climate AM2.5C360 are forced by 10-ensemble mean SST from the coupled model to focus on the global warming signal, while the other half of the ensemble members are forced by the first ensemble only SST to include some internal variability from the atmosphere-ocean coupling.

The difference between the “Historical” and “No Warming” scenarios represents the response of TCs to climate change since the pre-industrial levels, while the difference between the “Near/Far Future” and “Historical” scenarios represents the response of TCs to future climate change. Note that, to clearly illustrate the changes brought about by rising temperatures, the scenario differences in this study are all presented as warm scenarios minus relatively cold scenarios. The multi-year average SST gradually increases in the “Historical,” “Near Future,” and “Far Future” scenarios.

### TC stalling

TC stalling is defined as the behavior of a TC remaining within a 200 km radius for more than 48 h^3,4^. The 200 km radius yields a large enough sample size for statistical estimation and is close to the radius of the TCs’ inner core. Heavy rainfall and strong winds usually occur in the inner core of the TCs and their surrounding areas^67^. The track points satisfying the above criteria are termed “stalled points.” A stalled TC includes at least one stalled segment, defined as a sequence of nine or more consecutive 6-hourly stalled points. If the stalled segments identified based on different track points have at least one overlapping stalled point or if there is no break between the different stalled segments, these stalled segments are combined into a single stalled segment.

TCs with all the track points showing an MSW speed lower than or equal to 34 knots (17.5 m s^−1^) were excluded. To avoid the influence of extra-tropical transition on TC rainfall, the study region is constrained between 30°S and 30°N. The TCs in the South Atlantic are removed because the sample sizes are too small. The angular deviation between two successive 6-hourly track vectors v_1_ and v_2_ is $\theta={\cos }^{-1}({v}_{1}\cdot {v}_{2}/|{v}_{1}||{v}_{2}|)$. The TC translation speed (km h^−1^) of each track point is calculated by dividing the distance (km) from that point to the next point by their time interval. The absolute frequency is calculated by counting the number of 6-hourly TC track points.

The two crucial parameters in the definition of TC stalling are the minimum number of track points of the stalled segment, reflecting “stalled time,” and the “minimum enclosing circle radius” containing the corresponding stalled points. We calculated the cumulative density function of the minimum enclosing circle radius with 24 h, 36 h, 48 h, 60 h, and 72 h stalled time across all the basins over 1982–2019 based on observations (Supplementary Figs. 20–24). The results indicate that the number of identified stalled track points increases with increasing stalled time; conversely, the number of stalled track points decreases with increasing minimum enclosing circle radius. We selected 48 h as the stalled time and 200 km as the minimum enclosing circle radius because this combination is a compromise consideration between sample size and the extremeness of the events, and it is similar to what was used in a previous study^3^.

### TC rainfall

We aggregate the 3-hourly (MSWEP) rainfall fields into a 6-hourly field before assigning it to each 6-hourly TC track point. Rainfall greater than 0.6 mm 6 h^−1^ at a grid point is associated with the TC if the grid point is within 500 km from the TC center. Grids with rainfall greater than 0.6 mm 6 h^−1^ are defined as rainfall grids. Although significant rainfall may be observed outside the 500 km range from the TC center, this study used the commonly accepted 500 km threshold as a conservative choice to ensure greater confidence that the analyzed rainfall is caused by TCs. We define the area affected by the TC as a circle with a radius of 500 km centered on the points of the TC tracks.

We introduce several metrics to analyze the difference in climatology between stalled and non-stalled TC track points. The “overlap ratio” is calculated as the intersection area of TC-induced precipitation at two consecutive track points divided by their union area. For stalled segments, the rolling 24 h rainfall accumulation is determined by summing TC rainfall fields over each 24 h period and dividing the sum by the number of grid cells in the rainfall fields (with union areas counted only once), following the same method for non-stalled segments. The total area of the corresponding rainfall field is the rolling 24 h rainfall area. Also, we calculated the rainfall intensity and rainfall area for each 6-hourly track point.

We use daily rainfall to analyze the effect of climate warming on rainfall induced by stalled and non-stalled tracks. Daily rainfall at a grid point is associated with the TC if the grid point is within 500 km from the TC center at any time step of the day^13^. Grids with daily rainfall greater than 1 mm day^−1^ are defined as rainfall grids.

### Defining storm-local environments and storm characteristics

The storm-local environments reflect the environment experienced by TC track points. The 6-hourly data from ERA5 reanalysis were used to analyze the observed TC stalling, while the daily data from the AM2.5C360 model were linearly interpolated to a 6-hourly scale and then used to analyze the simulated TC stalling. Environmental variables include Steering Wind, Angle Diff, VWS, and High Alt. Area, and the presence of coexisting TC interactions. The steering winds are defined as the weighted average between winds at 850 hPa and 250 hPa^59^:

1 $${u}_{{steering}}=0.8\times {u}_{850}+0.2\times {u}_{200}$$

2 $${v}_{{steering}}=0.8\times {v}_{850}+0.2\times {v}_{200}$$

Vertical wind shear is computed as the square-root of the sum of the squared differences in the zonal and meridional winds between 850 and 250 hPa. The Angle Diff quantifies the directional misalignment between the TC motion and the environmental flow. It is defined as the absolute angular difference between the TC motion vector and the steering wind vector, constrained within a range of 0° to 180°. The TC motion vector is derived from the displacement vector extending from the current track point to the subsequent position. High Alt. Area is defined as the total area with an elevation exceeding 1000 m within a 500 km radius of the TC center. The 1000 m threshold was selected based on idealized numerical experiments indicating that terrain with a maximum height of this magnitude is sufficient to modify low-level flow of TC^68^. Coexisting TC interactions, widely known as the Fujiwhara effect^39^, describe the mutual dynamical influence between two spatially proximate cyclones driven by their respective vorticity fields, mass, and relative positioning. In this study, a coexisting TC interaction is defined to occur when the distance between the centers of two simultaneous TCs is no more than 2000 km.

Steering Wind (both zonal and meridional winds) and VWS were spectrally filtered to T11 resolution to remove cyclonic circulation features and retain only the large-scale, background environmental fields ref. ^14^ found that 95% of the T639-resolution cyclonic circulation is removed at a T11 truncation. Along-track sampling of mean values from the spectrally filtered fields and topographic data within a 500 km storm-centered radius was performed to obtain the storm-local environment variables.

Longitude and latitude serve as spatial proxies for the Coriolis force, SST, and large-scale circulation regimes. Although some studies found that local warmer SSTs stimulate earlier poleward TC recurvature in some cases^69,70^, detecting these thermal anomalies within a storm-local analysis framework can be challenging, as they may be statistically obscured by the large sample. However, the dynamical changes induced by SST are effectively captured by Angle Diff. Additionally, we used WindMax instead of the minimum pressure to characterize the intensity of TC because WindMax is more complete in the IBTrACS.

### Machine learning model and SHapley Additive exPlanations (SHAP) value

To investigate the dynamical drivers of TC stalling, we used the XGBoost algorithm^26^. XGBoost is a scalable implementation of the gradient tree boosting framework^71^ that has demonstrated good performance in various meteorological applications, including TC rainfall^72^ attribution and historical hurricane wind records^73^ reconstruction. Unlike traditional generalized linear models, XGBoost utilizes an ensemble of decision trees to model high-order interactions and non-monotonic relationships without requiring explicit functional form assumptions^26^.

The input feature vector consists of three storm characteristics (longitude, latitude, and WindMax) and five storm-local environmental variables (Steering Wind, Angle Diff, VWS, High Alt. Area, and coexisting TC interactions). The target variable is a binary label indicating whether a TC track point stalls. We optimized hyperparameters using a 5-fold cross-validation strategy. To prevent model overfitting, we divide the dataset into training (70%) and test (30%) sets, and preserve the original ratio of stalling samples in both subsets. The test set was only used for final model evaluation and was never involved in the cross-validation-based hyperparameter tuning process, thus avoiding data leakage.

We used the SHAP value method to interpret our XGBoost models. SHAP belongs to the method of ex post interpretation, drawing inspiration from Shapley values in cooperative game theory^27^. It provides a systematic mechanism to assign significance values to individual model features, helping quantify the impact of each feature on model predictions by considering all potential feature combinations^74^. The SHAP value for a specific feature represents its marginal contribution to the prediction, averaged over all possible feature permutations. For a given observation, the sum of SHAP values for all features, plus the model’s base value, equals the actual prediction output. The random seed initialization may significantly influence model training outcomes. To ensure the robustness of our results, we did not rely on a single fixed random seed for the data split; instead, all XGBoost and SHAP results reported in this study represent the ensemble mean derived from 50 independent training iterations with varying random seeds. In addition, the climatic characteristics of TC and its stalling definition determine the differences in the spatio-temporal distribution and class distribution of the samples, and such differences may affect the predictive ability of the model. Even so, the model still has good performances, with the Area Under Curve (AUC) values exceeding 0.83 and classification accuracy above 75% (Supplementary Table 2).

We utilized the TreeExplainer, a high-speed exact algorithm developed specifically for tree ensemble models^75^. In our analysis, a positive SHAP value indicates that the feature drives the model prediction towards “stalling,” while a negative value indicates a contribution towards “non-stalling.” We defined the feature importance as the mean value of absolute SHAP value. Furthermore, we analyzed SHAP dependence plots to reveal the functional form of the relationship between environmental variables and the probability of stalling.

The training of the XGBoost model and the SHAP analysis were conducted on a device equipped with an Intel Core i9-14900KF and a NVIDIA GeForce RTX 4070 GPU (GPU memory: 12GB) with data parallelization, with a total training time of 12 h.

### Validation of climate model simulations

Our high-resolution ensemble simulations reproduce important aspects of the observed frequency and rainfall of TC stalling, providing confidence in exploring the impacts of climate warming on TC stalling. Figure 2e–h shows the spatial pattern of observed and simulated ratios and daily rainfall of TC stalling based on the climate model results. The simulations well reproduce the regions with high observed TC stalling ratios based on observations. Notably, even in the region with a low total TC frequency, the simulations capture a local high stalling ratios in the central North Pacific Ocean. The simulations reproduce the spatial pattern of daily rainfall well, especially in capturing high values in the low-latitude central Pacific and northwestern Gulf of Mexico (stalled region of Hurricane Harvey).

We calculate the time series of both observed and simulated TC stalling ratios and daily rainfall under current climate conditions and estimate their long-term trends (Supplementary Figs. 4–7). In the NI basin, to avoid artifacts within the Indian Ocean gap, the validation period for TC stalling ratio of the climate model is limited to 1999–2019, while the validation period for other basins is 1982–2019. Additionally, in low- and mid-latitude regions without rain gauges, MSWEP data rely primarily on reanalysis data until 1999, after which satellite data become the dominant contributor^57^. Therefore, the validation period for daily rainfall from the climate model in all the basins is limited to 2000–2019, given the homogeneity and reliability of the MSWEP data. The results indicate that the simulations well reproduce the observed trends in TC stalling ratios and daily rainfall over time. Although the climate model does not reproduce daily rainfall as accurately as the frequency, the trends of ensemble mean, and most members are within the confidence intervals of the observed trends, indicating reliable model performance. We further evaluated the simulation performance of the model under TC stalling definitions with multiple combinations of the enclosing circle radius and the stalled time. The results indicate that the model consistently reproduces observed TC stalling patterns, regardless of the specific definition applied (Supplementary Figs. 25–34). This consistency confirms that our model’s performance is physically grounded rather than being an artifact under specific definitions, demonstrating its reliable capability to capture real TC dynamics.

However, some discrepancies between the simulations and the observations remain. Spatially, the model underestimates TC stalling ratios in the low-latitude regions of the SH. In areas where the observed stalling ratio is zero, the ensemble simulations produce low, but non-zero, values. These differences primarily arise from internal variability and the statistical properties of ensemble simulations^2,21,76,77^. The steering winds in these low-latitude regions are weak and volatile, and small variations in initial sea surface temperature conditions can shift the position of narrow convergence zones such as the SPCZ. Such sensitivity can lead to slight geographical misalignments and, consequently, an underestimation of stalling ratios in the ensemble mean.

The occurrence of “simulated non‑zero stalling where observations record zero” reflects a known advantage of ensemble modeling^78^: observational records represent only a single realization of the historical climate, whereas multi-member ensembles sample a broader probability distribution and can capture rare but physically plausible events that may not appear in the limited observational sample.

From a temporal perspective, differences in long-term trends primarily reflect internal climate variability. The observational record is relatively short and thus sensitive to interannual and decadal oscillations^16,22,79^, while the ensemble mean filters out such noise and emphasizes external forced, long-term signals. As a result, discrepancies between observed and simulated trends are expected and generally fall within the confidence intervals of the observed estimates.

In addition, because the WP basin contributed a large fraction of global TC stalling events, the model’s overestimation of daily rainfall in this basin influences the basin-wide and hemispheric estimates more strongly than in other regions, where such biases are comparatively small.

Overall, despite these discrepancies, the simulations reproduce the main spatio-temporal characteristics of TC stalling frequency and daily rainfall. To ensure the robustness of our conclusions, we adopt a conservative analytical approach that focuses on climatological differences across multi-decadal periods and emphasizes only those results that pass significance tests across the ensemble.

## Supplementary information

Supplementary Information

Transparent Peer Review file

## Data Availability

The IBTrACS v04 dataset is available from National Oceanic and Atmospheric Administration (NOAA), https://www.ncdc.noaa.gov/ibtracs. The MSWEP dataset is available from GloH2O, https://www.gloh2o.org/mswep/. All other data used in this study are available in the Zenodo database, 10.5281/zenodo.18954765).

## Code availability

The model source code is available from https://www.gfdl.noaa.gov/atmospheric-model/. Codes used in this study are available in the Zenodo database, 10.5281/zenodo.18954765). Details about the code and technology of XGBoost are available from https://xgboost.readthedocs.io/en/stable/index.html.
