# Supplementary material for: Global stalled tropical cyclones in a changing climate
Source: Nat Commun. 2026 Mar 30;17:5145. doi: 10.1038/s41467-026-71320-3 (PMC13249938; doi:10.1038/s41467-026-71320-3)
Supplement: Supplementary file 1 — Supplementary Information [file 41467_2026_71320_MOESM1_ESM.pdf]

Supplementary Information

Global stalled tropical cyclones in a changing climate

Zifeng Deng[1, 2], Gabriele Villarini[2, 4*], Gabriel A. Vecchi[3, 4], Wenchang Yang[3], Zhaoli Wang[1, 5*]

[1]School of Civil Engineering and Transportation, State Key Laboratory of Subtropical Building and Urban Science, South China University of Technology, Guangzhou, China.

[2]Department of Civil and Environmental Engineering, Princeton University, Princeton, NJ, USA.

[3]Department of Geosciences, Princeton University, Princeton, NJ, USA.

[4]High Meadows Environmental Institute, Princeton University, Princeton, NJ, USA.

[5]Pazhou Lab, Guangzhou, China.

*Corresponding authors: Gabriele Villarini (gvillari@princeton.edu), Zhaoli Wang (wangzhl@scut.edu.cn)

This document contains:

Supplementary Figs. 1–34

Supplementary Tables 1–4

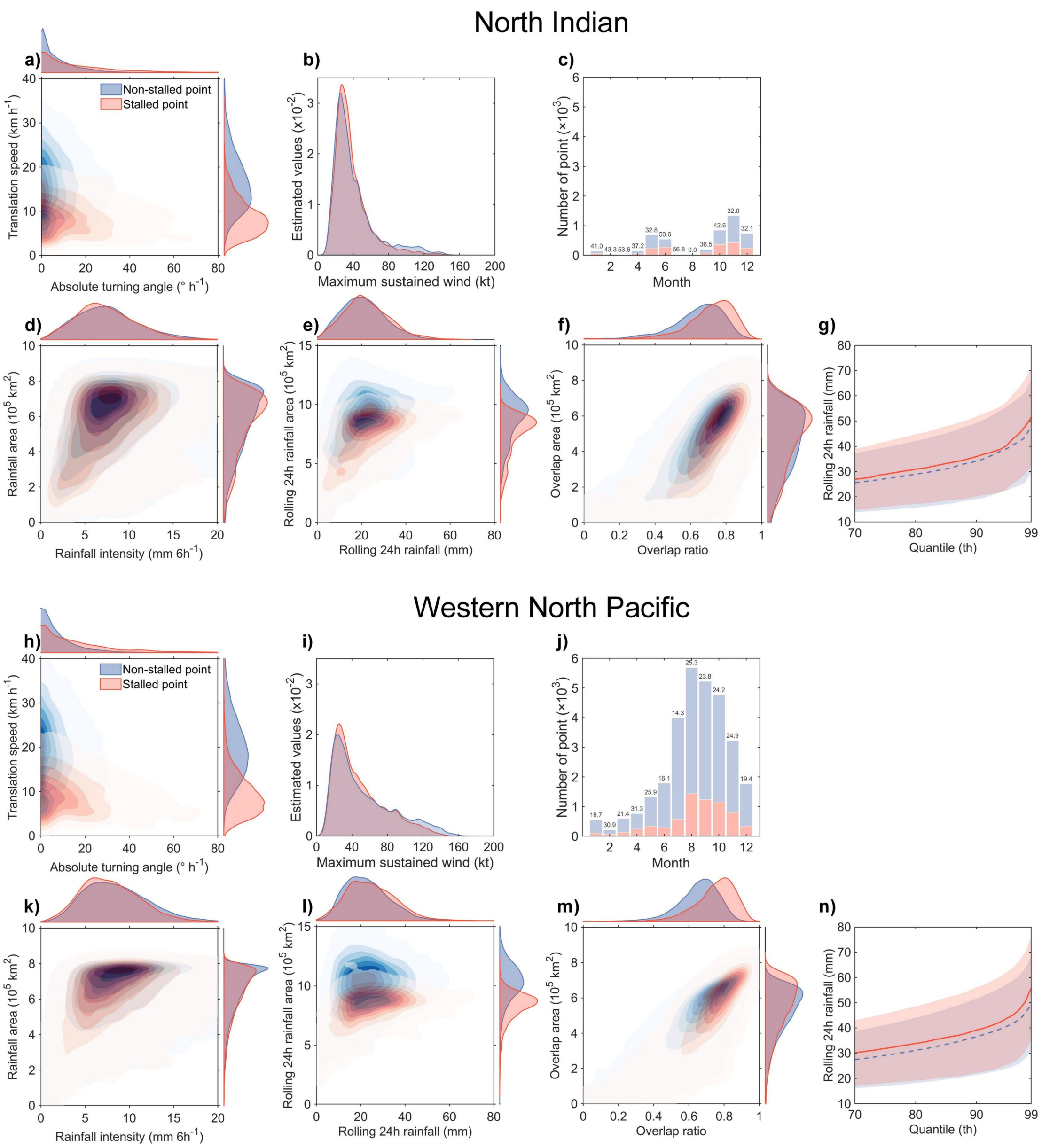

Supplementary Fig. 1 | General characteristics of stalled and non-stalled of tropical cyclone (TC) track points based on the observations in North Indian and Western North Pacific basins. The two-dimensional kernel density estimations of (**a,h**) translation speed against absolute turning angle, (**d,k**) rainfall area against rainfall intensity at 6-hour timescale, (**e,l**) rolling 24h rainfall area against rolling 24h rainfall, and (**f,m**) the overlap area against overlap ratio for the stalled and non-stalled TC track points (see Methods). The kernel density estimations of (**b,i**) maximum sustained wind (MSW) (kt). (**c,j**) Histograms of the frequency of the stalled and non-stalled TC track points in the seasonal cycle. Values of the areal mean rolling 24h rainfall fields between the 70th and 99th percentiles are shown in (**g,n**), with the shading denoting 95% confidence intervals of each percentile.

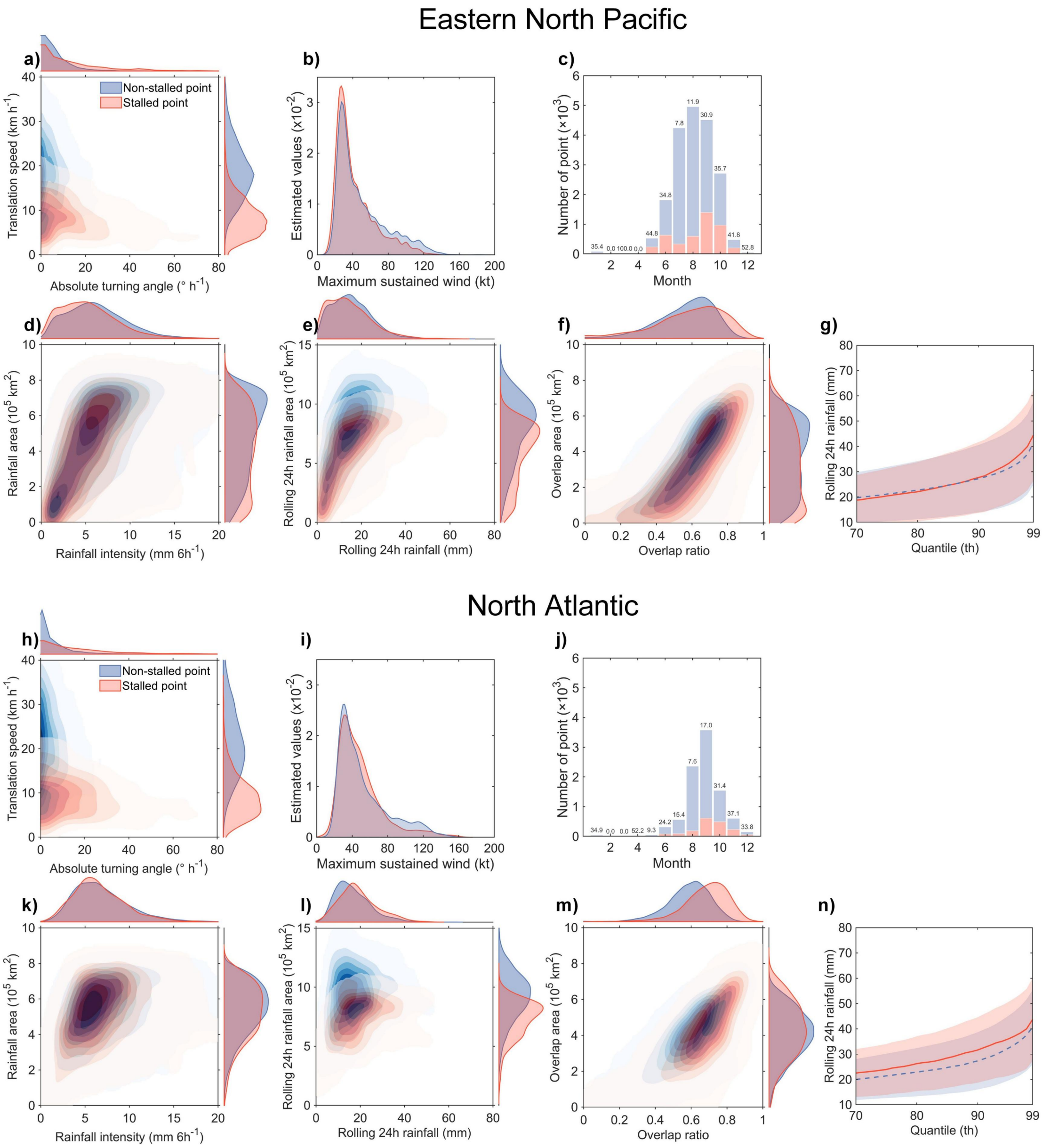

Supplementary Fig. 2 | General characteristics of stalled and non-stalled of tropical cyclone (TC) track points based on the observations in Eastern North Pacific and North Atlantic basins. The two-dimensional kernel density estimations of (**a,h**) translation speed against absolute turning angle, (**d,k**) rainfall area against rainfall intensity at 6-hour timescale, (**e,l**) rolling 24h rainfall area against rolling 24h rainfall, and (**f,m**) the overlap area against overlap ratio for the stalled and non-stalled TC track points (see Methods). The kernel density estimations of (**b,i**) maximum sustained wind (MSW) (kt). (**c,j**) Histograms of the frequency of the stalled and non-stalled TC track points in the seasonal cycle. Values of the areal mean rolling 24h rainfall fields between the 70th and 99th percentiles are shown in (**g,n**), with the shading denoting 95% confidence intervals of each percentile.

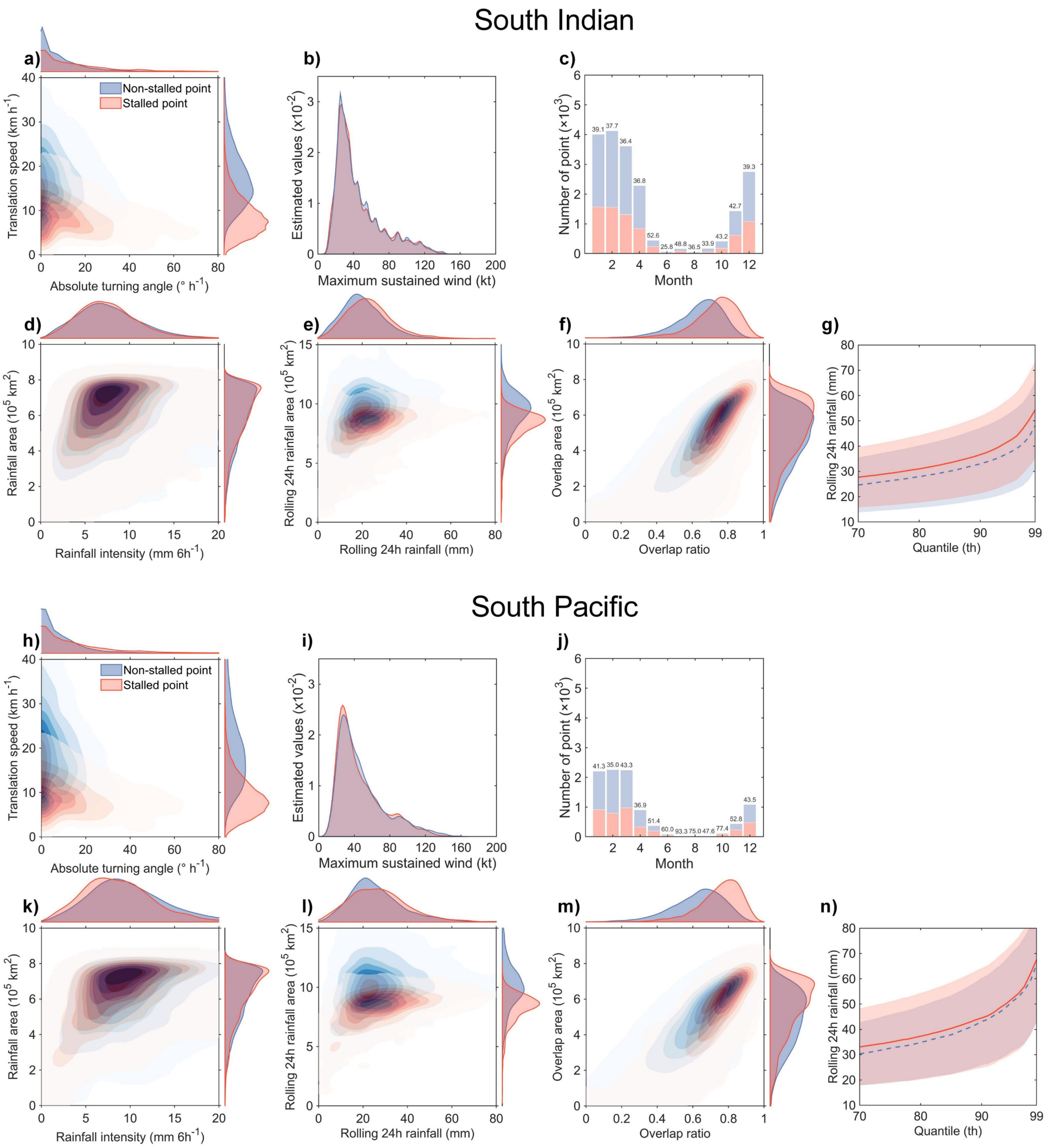

Supplementary Fig. 3 | General characteristics of stalled and non-stalled of tropical cyclone (TC) track points based on the observations in South Indian and South Pacific basins. The two-dimensional kernel density estimations of (**a,h**) translation speed against absolute turning angle, (**d,k**) rainfall area against rainfall intensity at 6-hour timescale, (**e,l**) rolling 24h rainfall area against rolling 24h rainfall, and (**f,m**) the overlap area against overlap ratio for the stalled and non-stalled TC track points (see Methods). The kernel density estimations of (**b,i**) maximum sustained wind (MSW) (kt). (**c,j**) Histograms of the frequency of the stalled and non-stalled TC track points in the seasonal cycle. Values of the areal mean rolling 24h rainfall fields between the 70th and 99th percentiles are shown in (**g,n**), with the shading denoting 95% confidence intervals of each percentile.

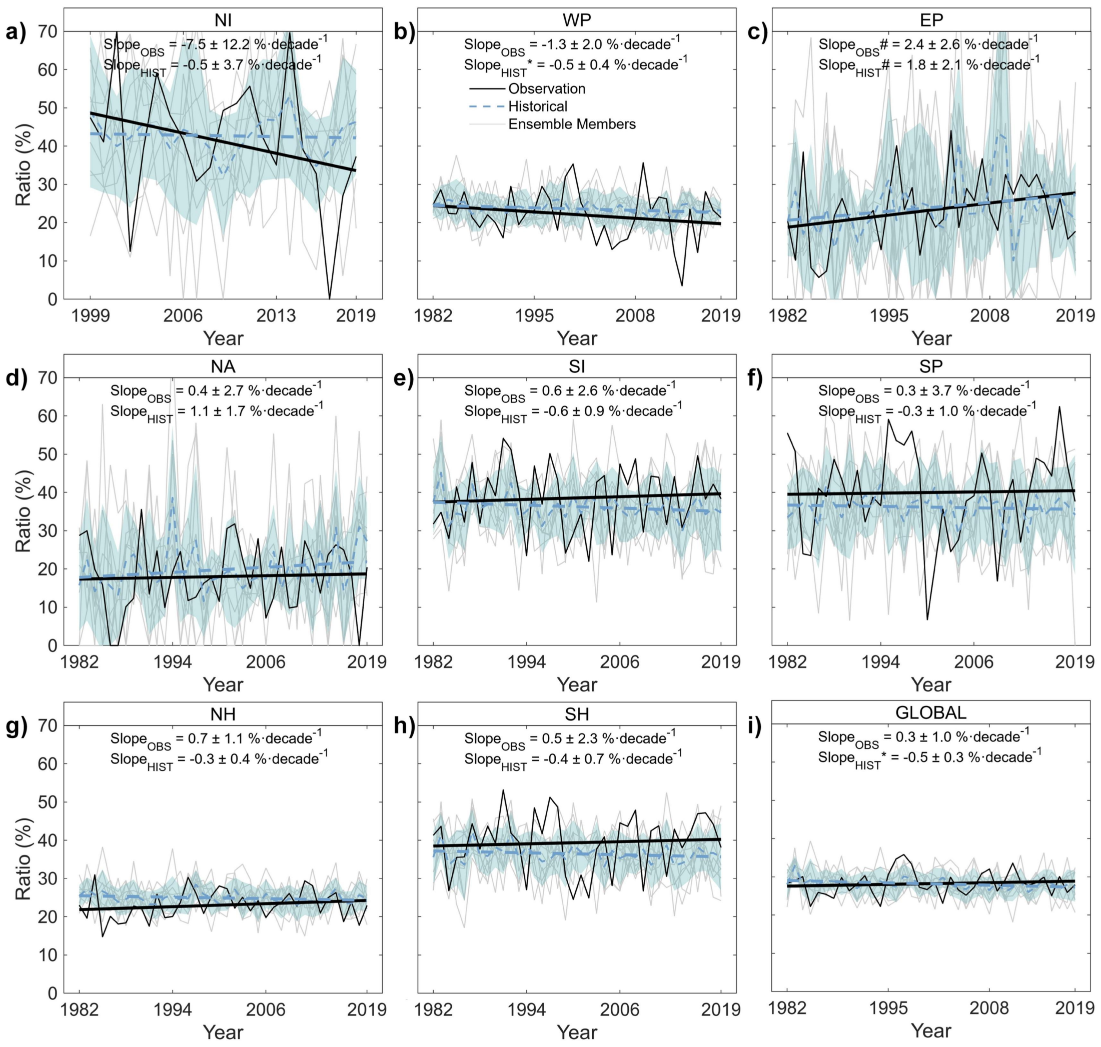

Supplementary Fig. 4 | Time series and their trends of observed and simulated tropical cyclone (TC) stalling ratios. The time series of simulated TC stalling ratios (blue dash line) are the average of ten members in the simulated results, with the shading denoting one standard deviation of the member results (see Methods). Black lines represent the time series of observed TC stalling ratio. Gray lines represent the time series of simulated TC stalling ratio of ten members. The observed and simulated trends are estimated by ordinary least squares method. The symbols "*" and "#" indicate that the trend line has a statistically significant slope at the 5% and 10% level, respectively. The NI, WP, EP, NA, SI, SP, NH, and SH represent the North Indian, Western North Pacific, Eastern North Pacific, North Atlantic, South Indian, South Pacific, North Hemisphere, and South Hemisphere, respectively.

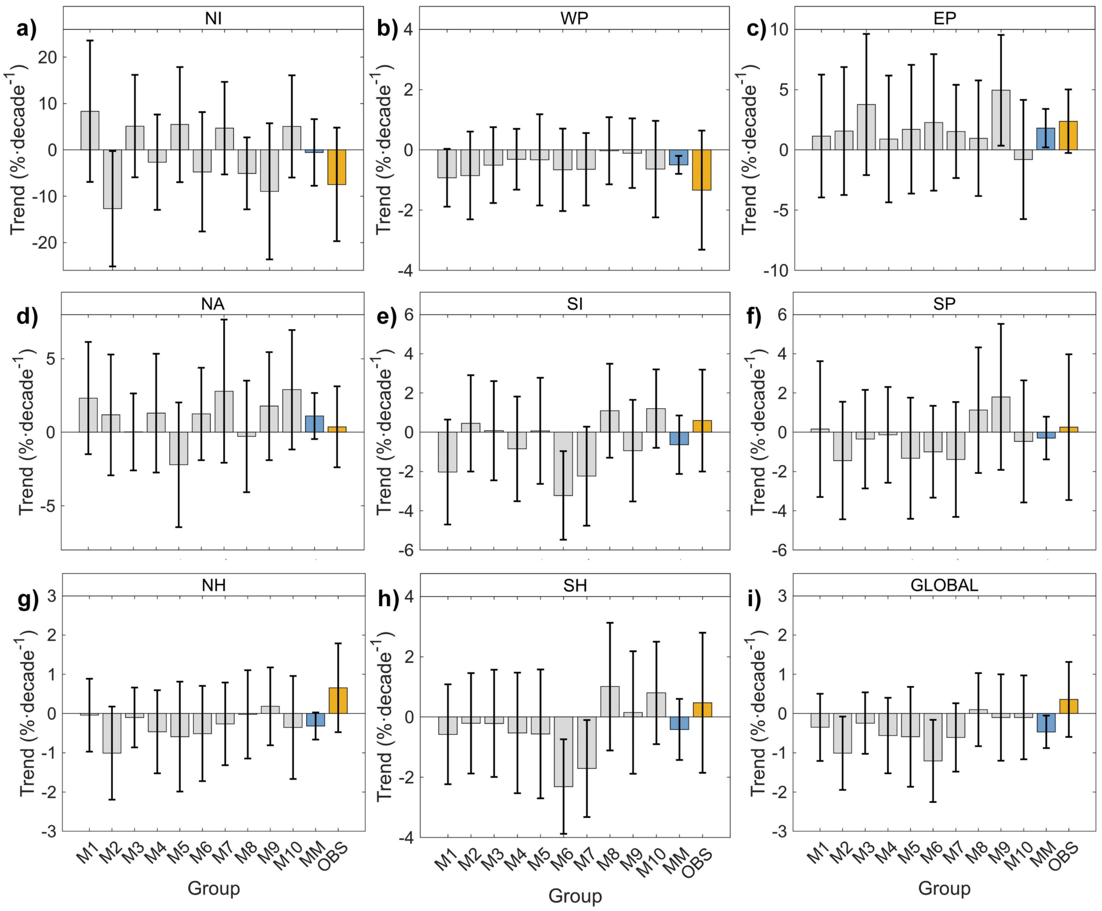

Supplementary Fig. 5 | Trends of time series of observed and simulated tropical cyclone (TC) stalling ratios. The trends of the simulated TC stalling ratios for the ten members of the "Historical" scenario are shown in gray bars, while the trends of their ensemble mean time series are shown as blue bars. The trends in the observed TC stalling ratios are shown in yellow bars. Error bars are the 95% confidence intervals for these trends. "M1"-"M10" represent the results of members one to ten of the "Historical" scenario, respectively. "MM" represents the results of the ensemble mean of the simulations of ten members. "OBS" represents the results of the observations. The NI, WP, EP, NA, SI, SP, NH, and SH represent the North Indian, Western North Pacific, Eastern North Pacific, North Atlantic, South Indian, South Pacific, North Hemisphere, and South Hemisphere, respectively.

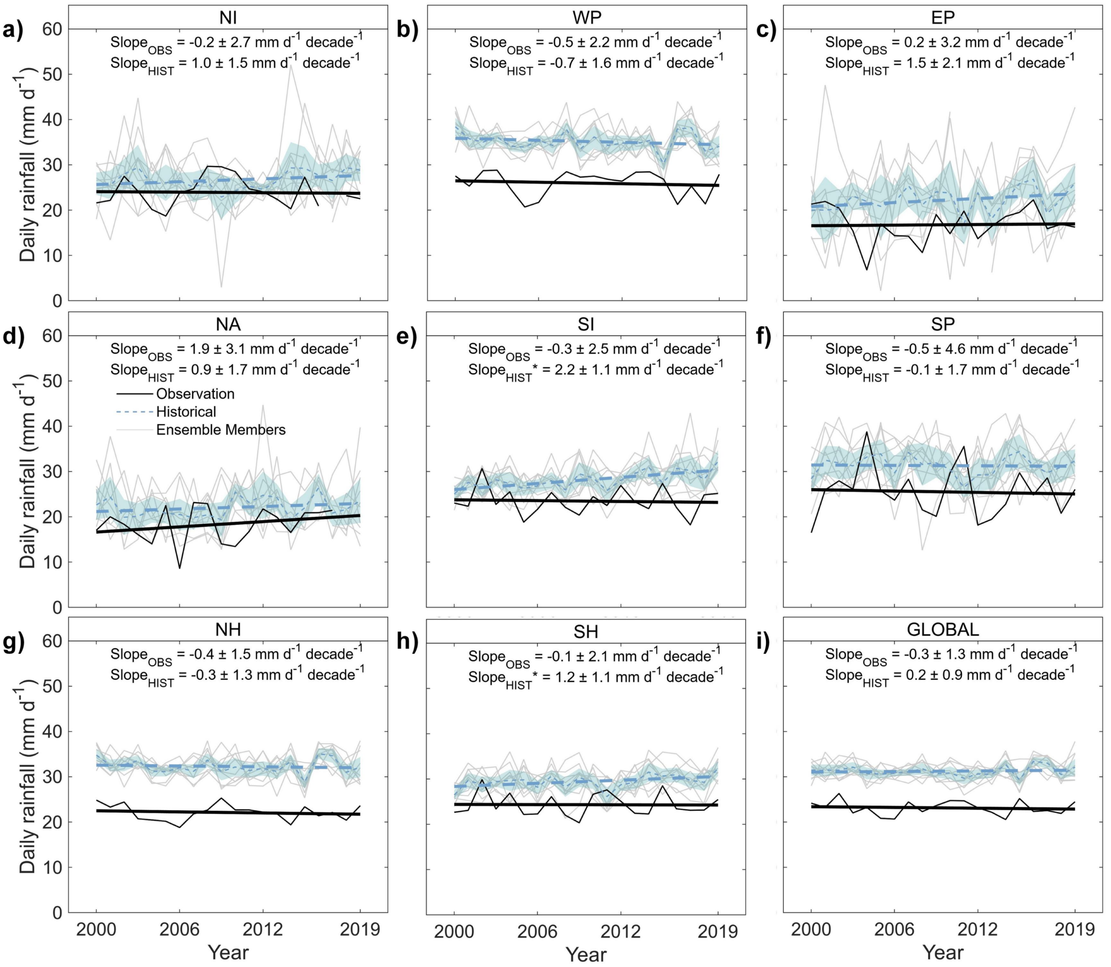

Supplementary Fig. 6 | Time series and trends of observed and simulated daily rainfall of tropical cyclone (TC) stalling. The time series of simulated TC stalling ratios (blue dash line) are the average of ten members in the simulated results, with the shading denoting one standard deviation of the member results (see Methods). Black lines represent the time series of observed TC stalling ratios. Gray lines represent the time series of simulated TC stalling ratios of ten members. The observed and simulated trends are estimated by ordinary least squares method. The symbol "*" indicates that the trend line has a statistically significant slope at the 5% level. The NI, WP, EP, NA, SI, SP, NH, and SH represent the North Indian, Western North Pacific, Eastern North Pacific, North Atlantic, South Indian, South Pacific, North Hemisphere, and South Hemisphere, respectively.

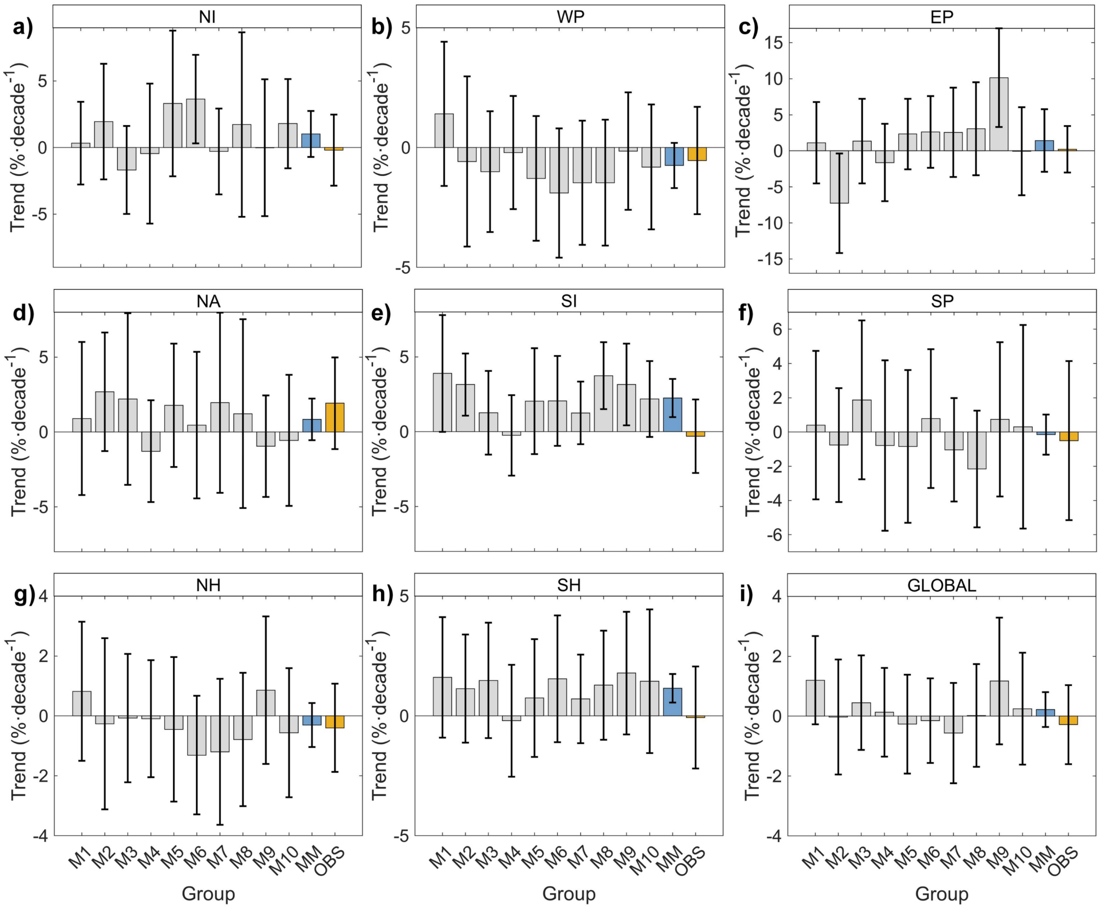

Supplementary Fig. 7 | Trends of time series of observed and simulated daily rainfall of tropical cyclone (TC) stalling. The trends of the simulated daily rainfall of TC stalling for the ten members of the "Historical" scenario are shown in gray bars, while the trends of their ensemble mean time series are shown as blue bars. The trends in the observed TC stalling ratios are shown in yellow bars. Error bars are the 95% confidence intervals for these trends. "M1"-"M10" represent the results of members one to ten of the "Historical" scenario, respectively. "MM" represents the results of the ensemble mean of the simulations of ten members. "OBS" represents the results of the observations. The NI, WP, EP, NA, SI, SP, NH, and SH represent the North Indian, Western North Pacific, Eastern North Pacific, North Atlantic, South Indian, South Pacific, North Hemisphere, and South Hemisphere, respectively.

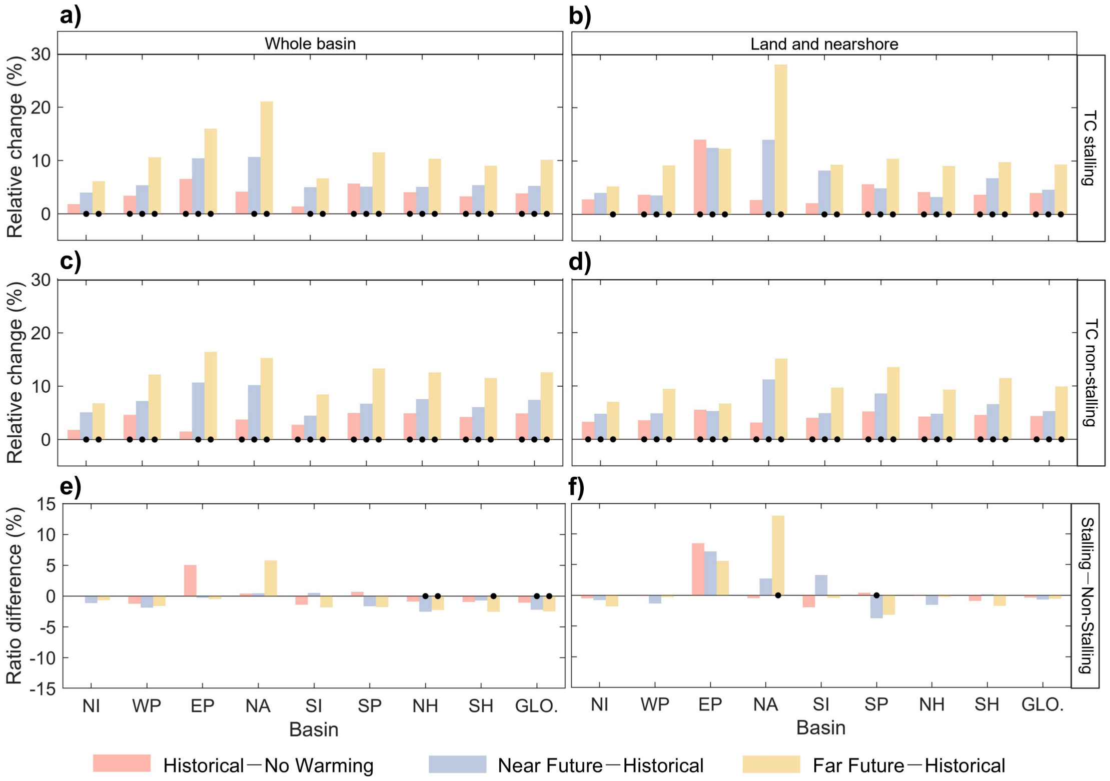

Supplementary Fig. 8 | Changes in the daily rainfall of tropical cyclone (TC) stalling and TC basins under global warming. Relative changes in daily rainfall of TC stalling (**a, b**) and non-stalling (**c, d**) between "Historical" scenario and "No Warming," "Near Future," and "Far Future" scenarios are calculated in the whole basin (**a, c, e**) and the land nearshore regions (**b, d, f**). The differences of the relative changes between TC stalling and non-stalling are shown in panels **e**, **f**. Black dots in panels **a-d** indicate differences significant at the 5% level based on two-tail t-tests. Black dots in panels **e-f** indicate the differences significant between relative changes in the TC stalling and non-stalling at the 5% level based on bootstrap tests. The NI, WP, EP, NA, SI, SP, NH, and SH represent the North Indian, Western North Pacific, Eastern North Pacific, North Atlantic, South Indian, South Pacific, North Hemisphere, and South Hemisphere, respectively. "Hist" represents the "Historical" scenario, "NW" represents the "No Warming" scenario, "NF" represents the "Near Future" scenario, and "FF" represents the "Far Future" scenario.

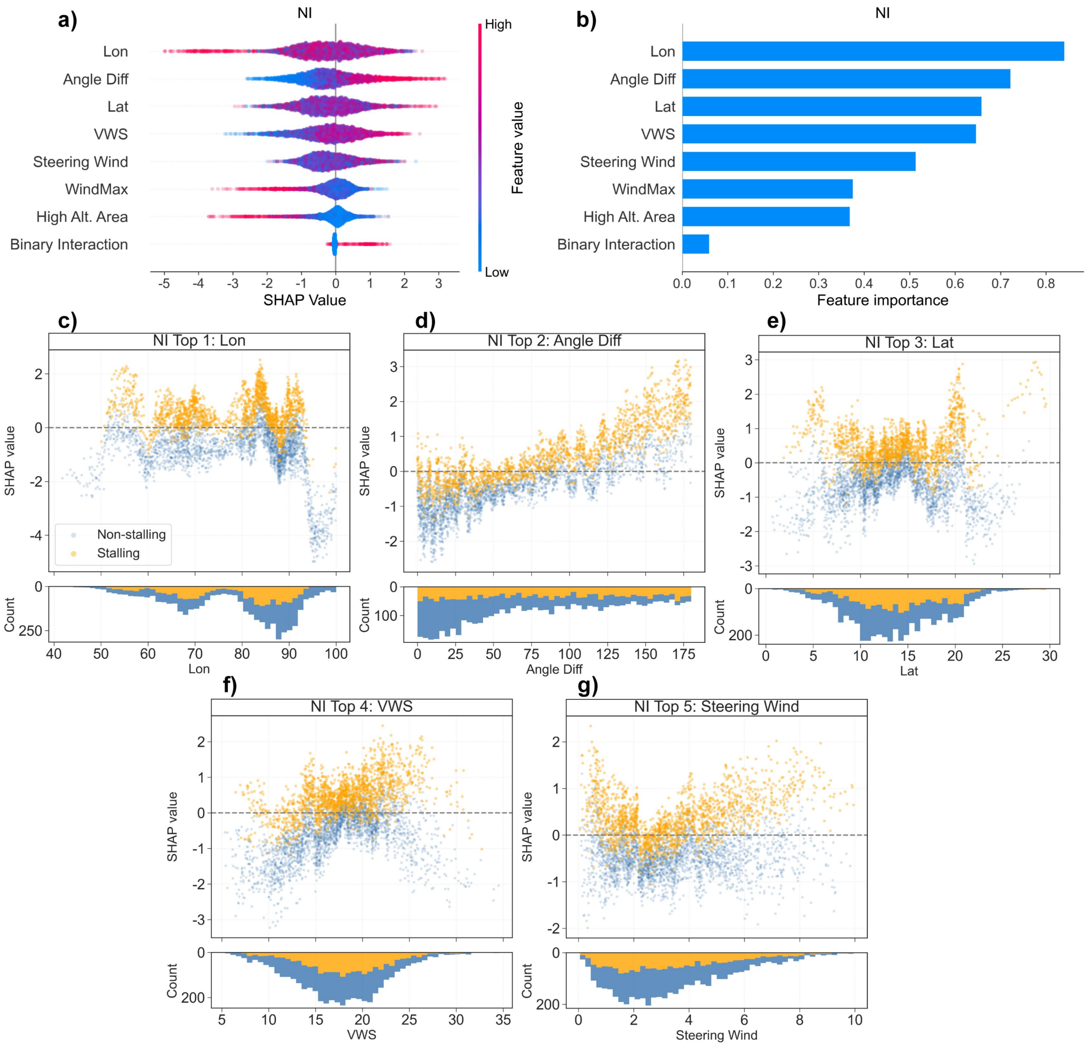

Supplementary Fig. 9 | SHapley Additive exPlanations (SHAP) analysis from the XGBoost model for tropical cyclone (TC) stalling in the North Indian (NI) basin. Beeswarm summary plot (**a**), feature importance (**b**) and relationship between the five most important features and stalling probability SHAP value (**c - g**) based on the XGBoost model in the NI basin.

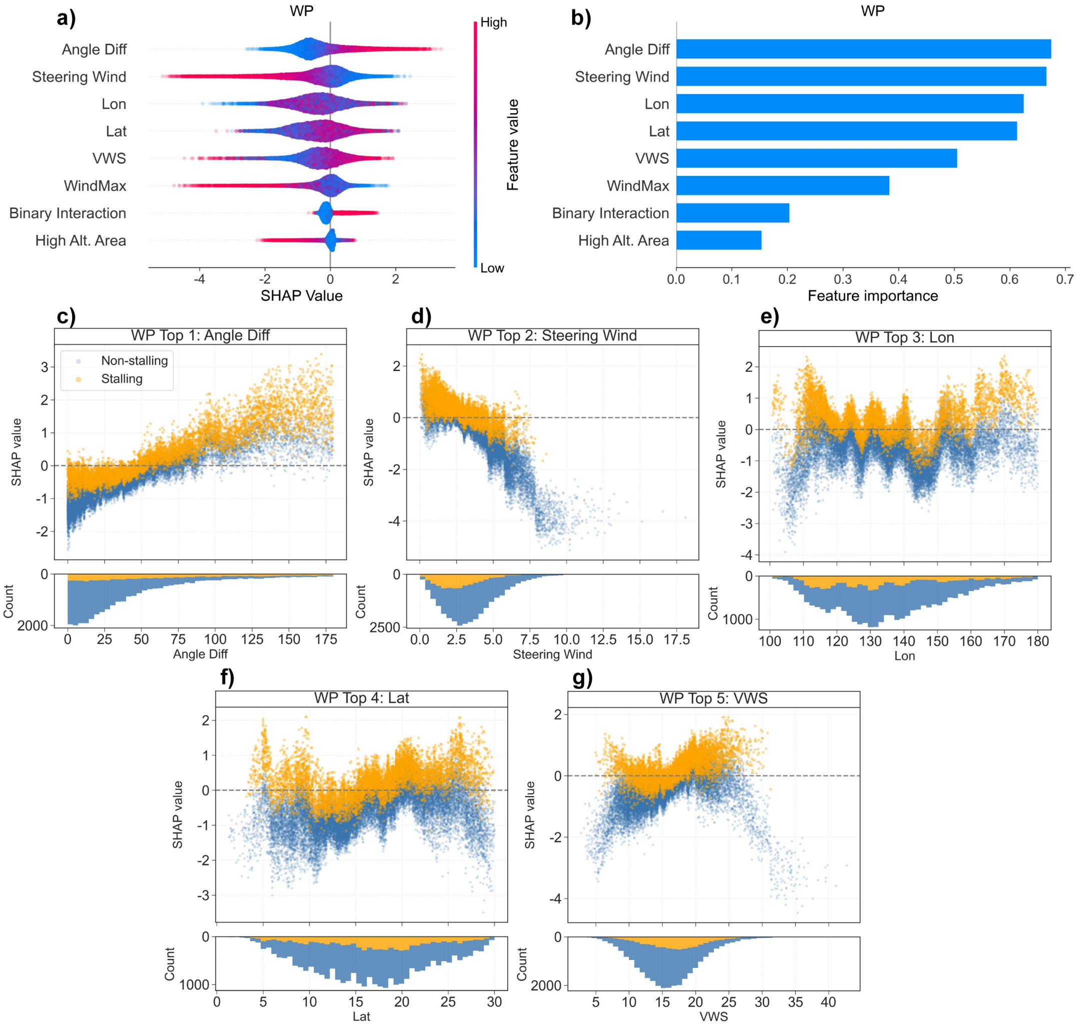

Supplementary Fig. 10 | SHapley Additive exPlanations (SHAP) analysis from the XGBoost model for tropical cyclone (TC) stalling in the Western North Pacific (WP) basin. Beeswarm summary plot (**a**), feature importance (**b**) and relationship between the five most important features and stalling probability SHAP value (**c - g**) based on the XGBoost model in the WP basin.

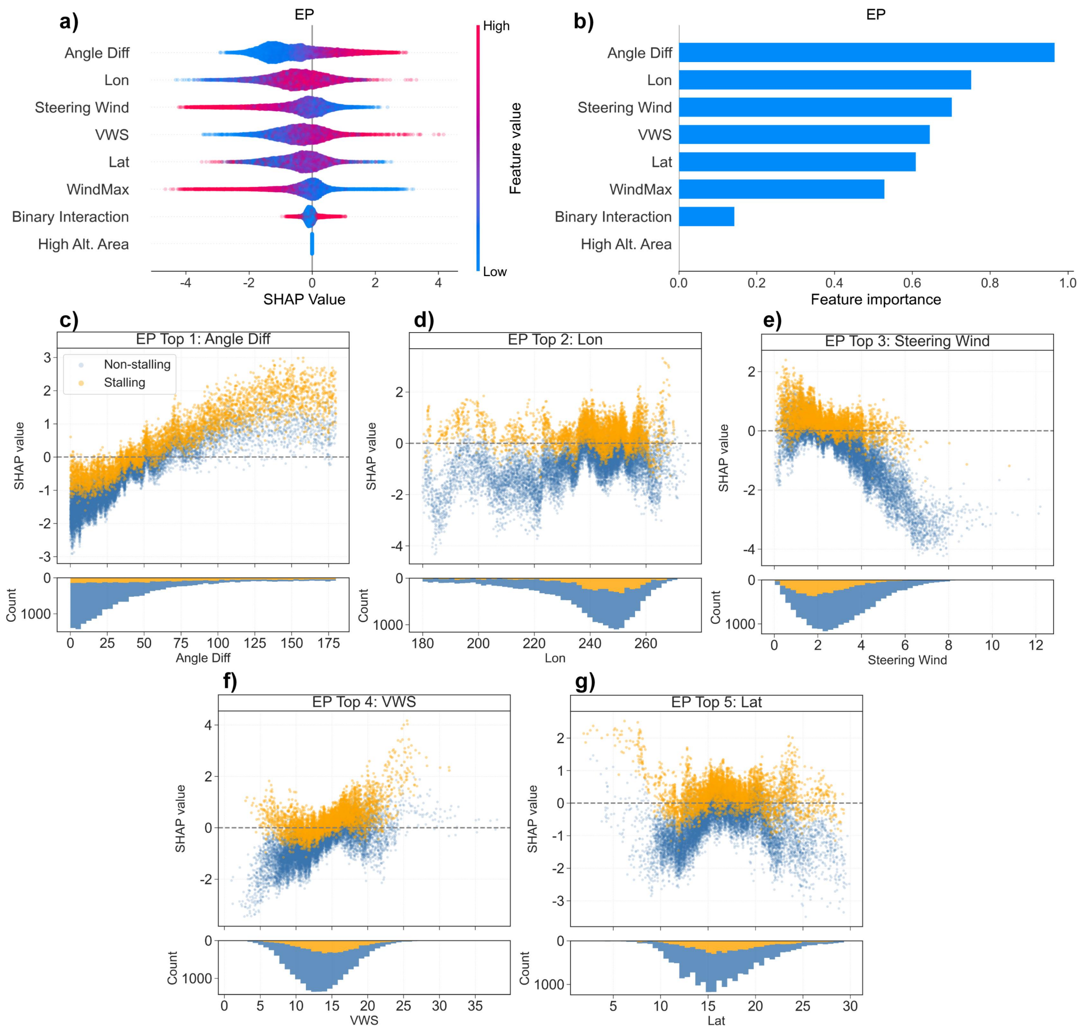

Supplementary Fig. 11 | SHapley Additive exPlanations (SHAP) analysis from the XGBoost model for tropical cyclone (TC) stalling in the Eastern North Pacific (EP) basin. Beeswarm summary plot (**a**), feature importance (**b**) and relationship between the five most important features and stalling probability SHAP value (**c - g**) based on the XGBoost model in the EP basin.

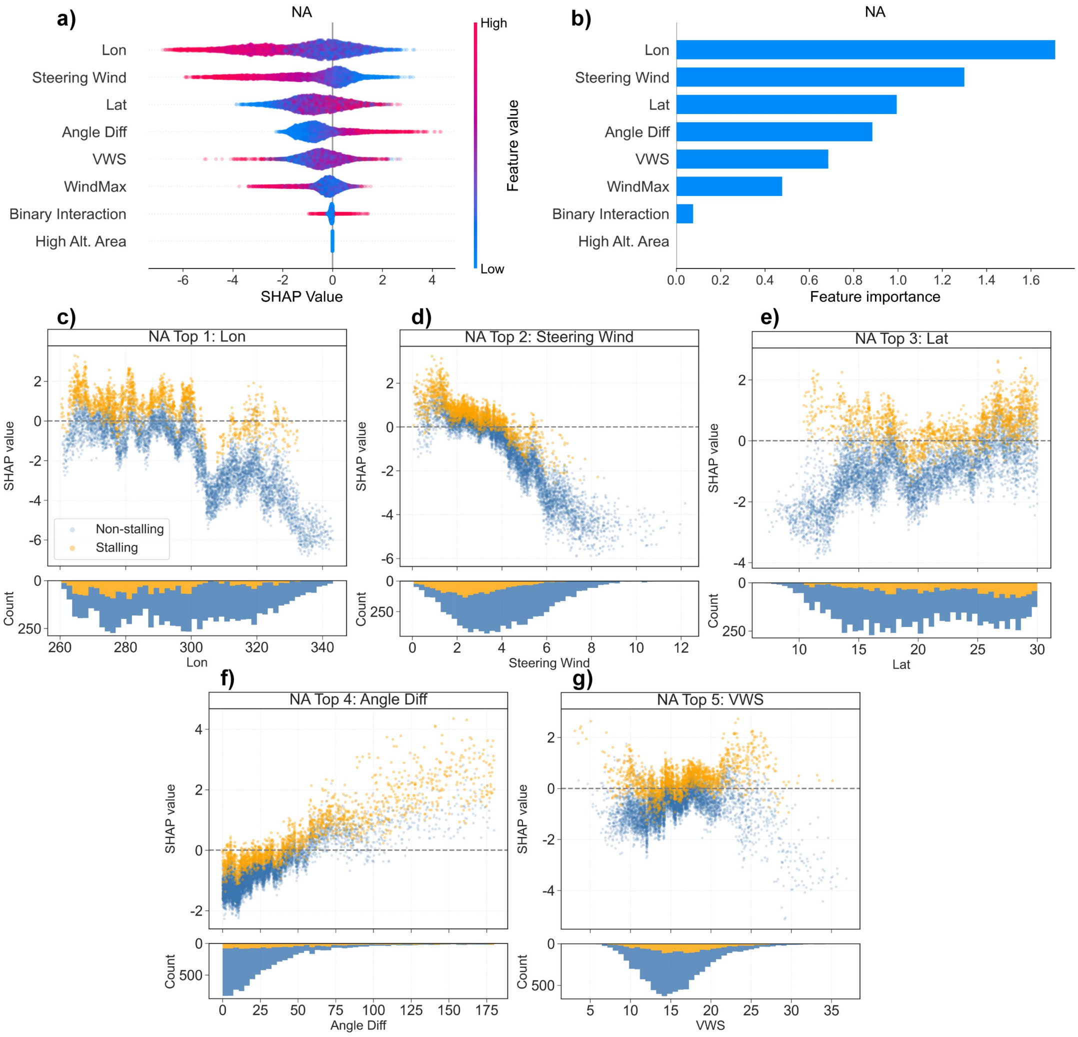

Supplementary Fig. 12 | SHapley Additive exPlanations (SHAP) analysis from the XGBoost model for tropical cyclone (TC) stalling in the North Atlantic (NA) basin. Beeswarm summary plot (**a**), feature importance (**b**) and relationship between the five most important features and stalling probability SHAP value (**c - g**) based on the XGBoost model in the NA basin.

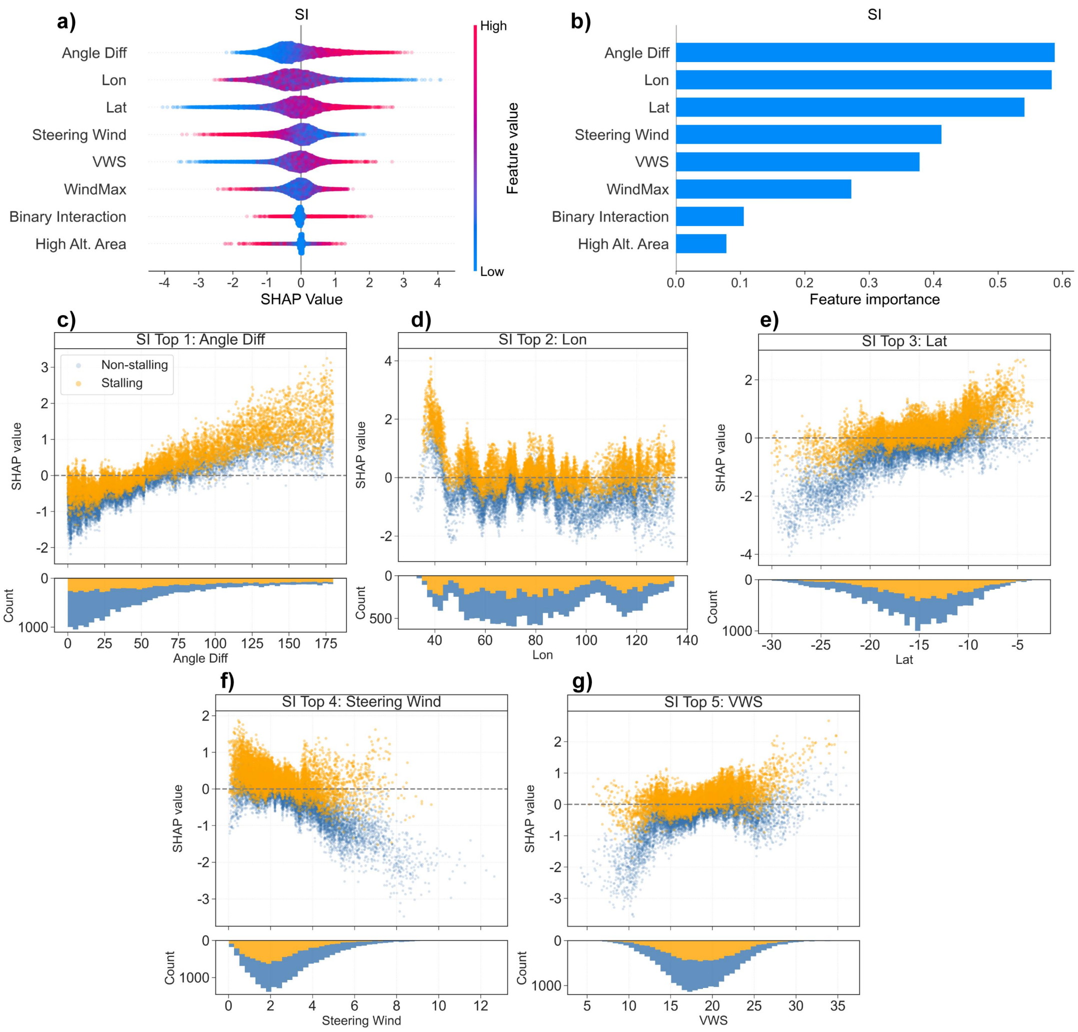

Supplementary Fig. 13 | SHapley Additive exPlanations (SHAP) analysis from the XGBoost model for tropical cyclone (TC) stalling in the South Indian (SI) basin. Beeswarm summary plot (**a**), feature importance (**b**) and relationship between the five most important features and stalling probability SHAP value (**c - g**) based on the XGBoost model in the SI basin.

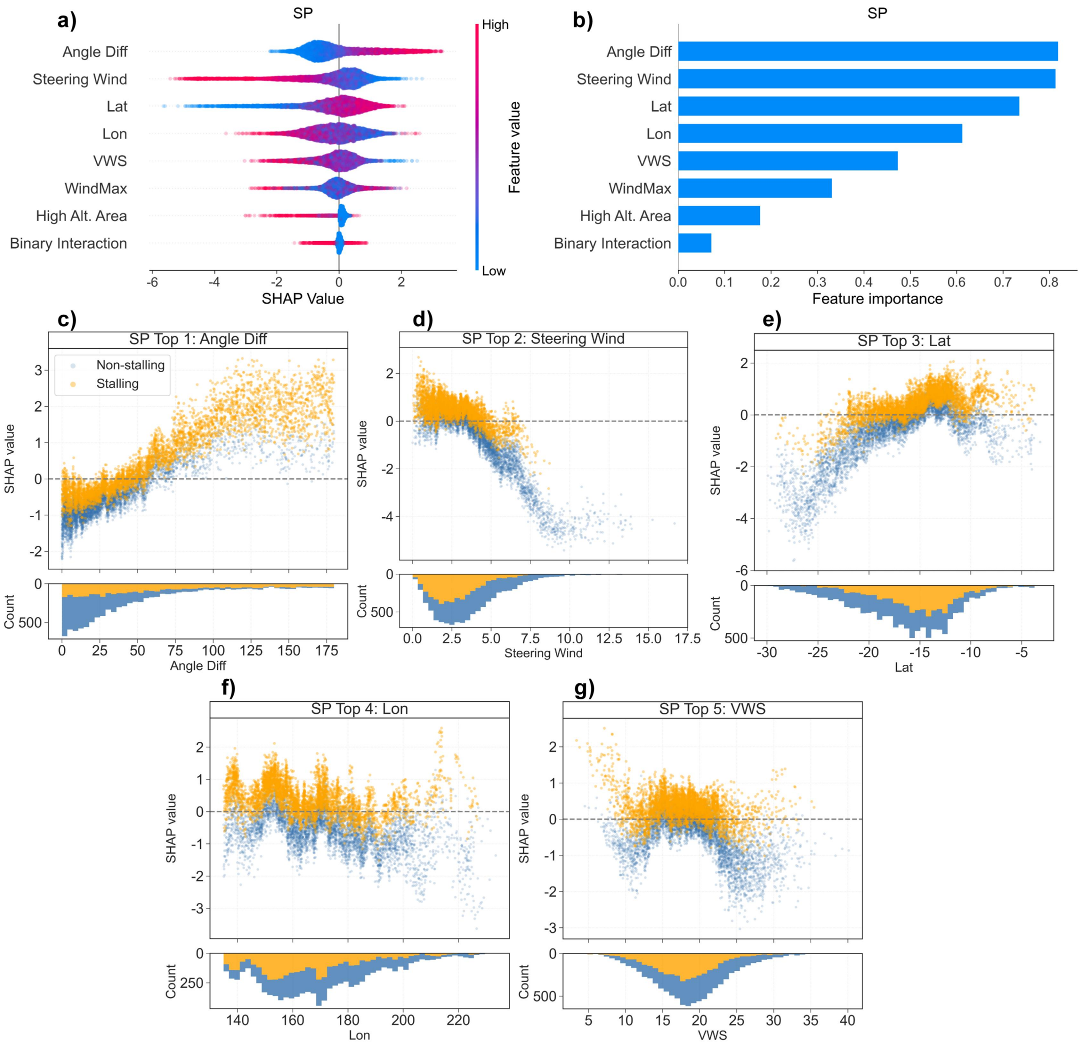

Supplementary Fig. 14 | SHapley Additive exPlanations (SHAP) analysis from the XGBoost model for tropical cyclone (TC) stalling in the South Pacific (SP) basin. Beeswarm summary plot (**a**), feature importance (**b**) and relationship between the five most important features and stalling probability SHAP value (**c - g**) based on the XGBoost model in the SP basin.

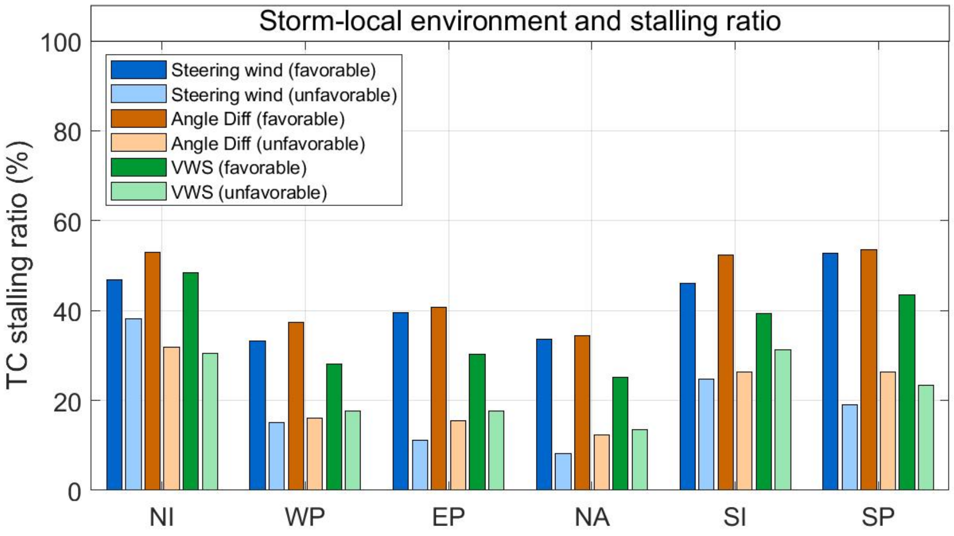

Supplementary Fig. 15 | Storm-local environment and stalling ratio. Histograms for all the basins and three ERA5 environmental variables (steering wind, Angle Diff and VWS) showing the probability of tropical cyclone (TC) stalling for cases' environmental variables being within the stalling-inducing intervals and the probability of TC stalling for cases' environmental variables not being within the stalling-inducing intervals. Critical thresholds are calculated by solving a logit equation for the environmental variable value that yields the average basin-wide probability of TC stalling. The "favorable" in the legend that the environment variable is in stalling-inducing interval, in which the environment variable is conducive to the occurrence of TC stalling. The NI, WP, EP, NA, SI, and SP represent the North Indian, Western North Pacific, Eastern North Pacific, North Atlantic, South Indian, and South Pacific, respectively.

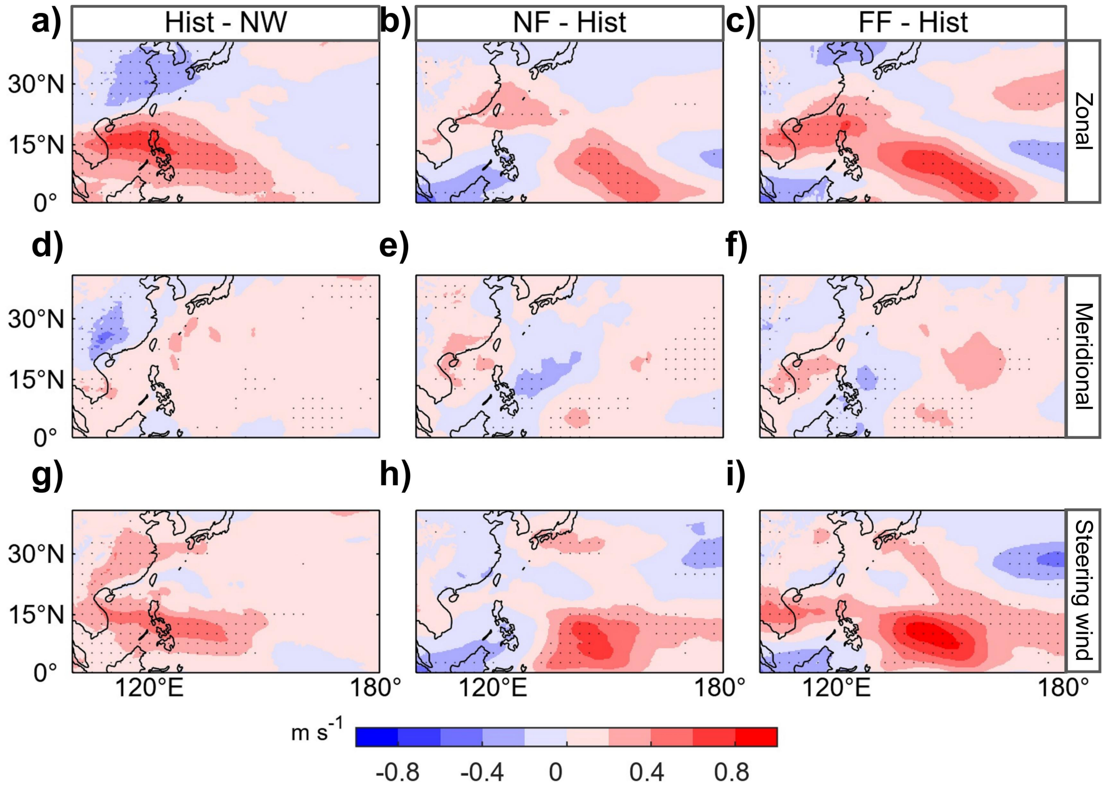

Supplementary Fig. 16 | Changes in the steering winds under climate change. Changes in zonal wind (**a**, **b**, **c**), Meridional wind (**d**, **e**, **f**), and steering wind (**g**, **h**, **i**) are computed as July to November averages in the "Historical" scenario and those in the "No Warming," "Near Future," and "Far Future" scenarios. Stars show where the difference is not statistically significant, based on a two-tailed t test at 95% level. "Hist" represents the "Historical" scenario, "NW" represents the "No Warming" scenario, "NF" represents the "Near Future" scenario, and "FF" represents the "Far Future" scenario.

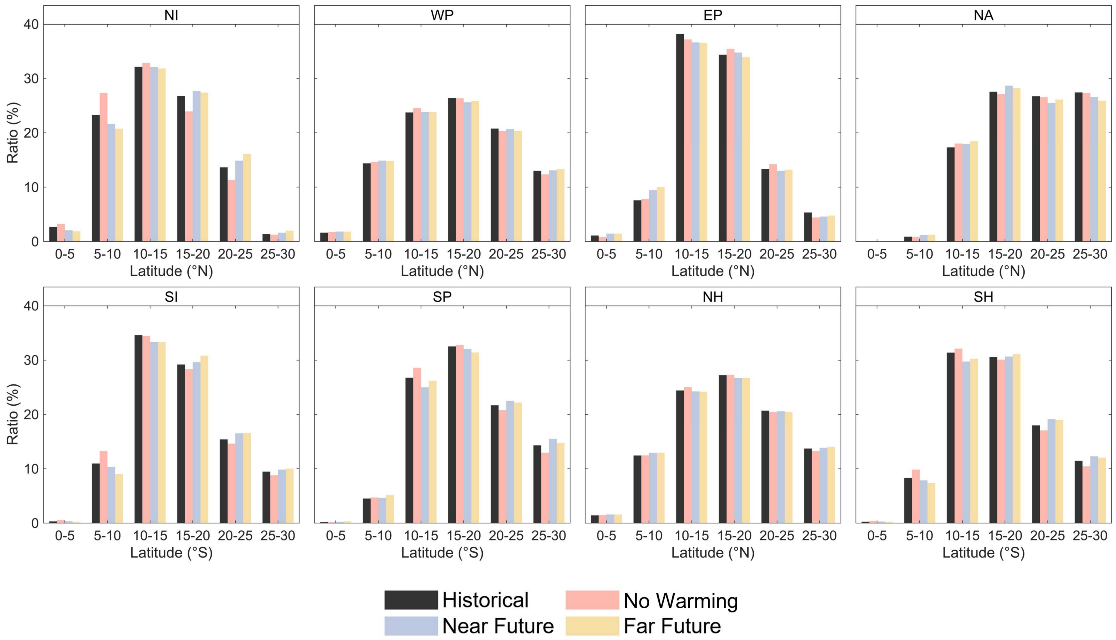

Supplementary Fig. 17 | Changes in zonal distribution of tropical cyclone (TC) track points in model simulations. The ratio of TC track points in each zonal interval to the total track points for each basin in "Historical," "No Warming," "Near Future," and "Far Future" scenarios. The NI, WP, EP, NA, SI, SP, NH, and SH represent the North Indian, Western North Pacific, Eastern North Pacific, North Atlantic, South Indian, South Pacific, North Hemisphere, and South Hemisphere, respectively.

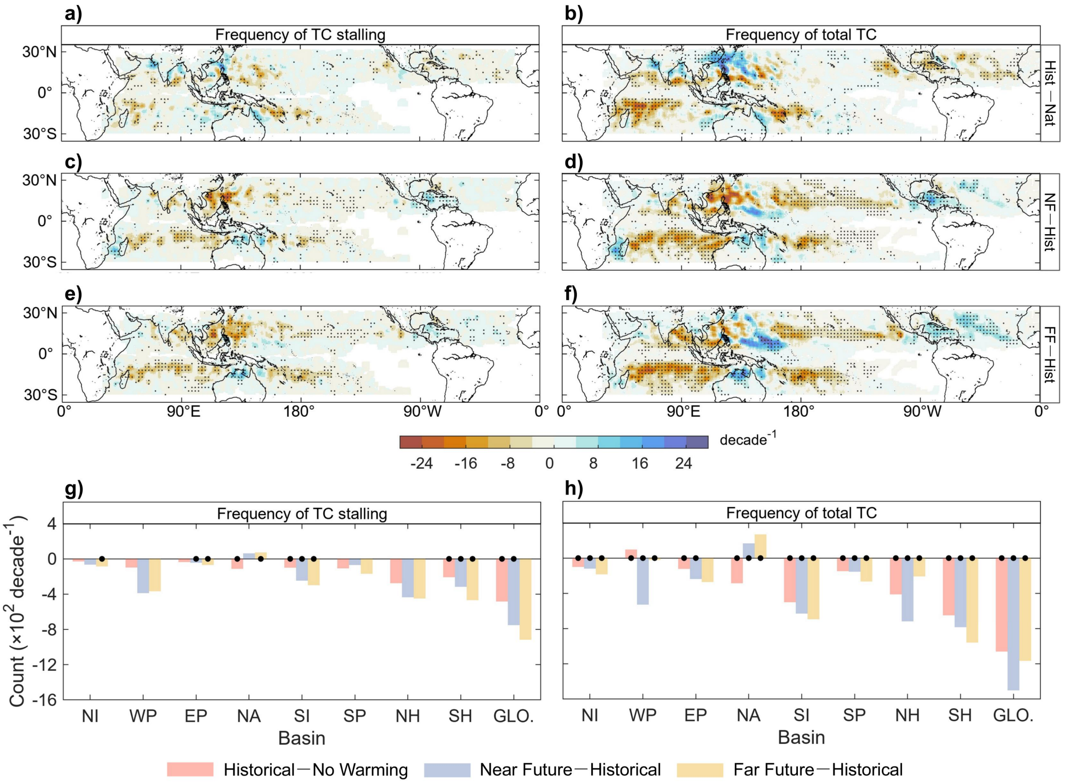

Supplementary Fig. 18 | Changes of frequency of the tropical cyclone (TC) stalling and total TC under global warming. Spatial changes of frequency of the TC stalling (**a, c, e**) and total TC (**b, d, f**) between "Historical" scenario and "No Warming," "Near Future," and "Far Future" scenarios. Changes in track point number of TC stalling (**g**) and total TC (**h**) between "Historical" scenario and "No Warming," "Near Future," and "Far Future" scenarios are calculated across the whole basin. To smooth the spatial results, the results for TC frequency are shown on grids with a spatial resolution of 0.5°, and the result for each 0.5° × 0.5° grid are the result within a 4° × 4° area centered on that grid. "Hist" represents the "Historical" scenario, "NW" represents the "No Warming" scenario, "NF" represents the "Near Future" scenario, and "FF" represents the "Far Future" scenario. Black dots indicate significant differences at the 5% level using bootstrap method in panel **a - f** and using two-tail t-tests in panel **g - h**.

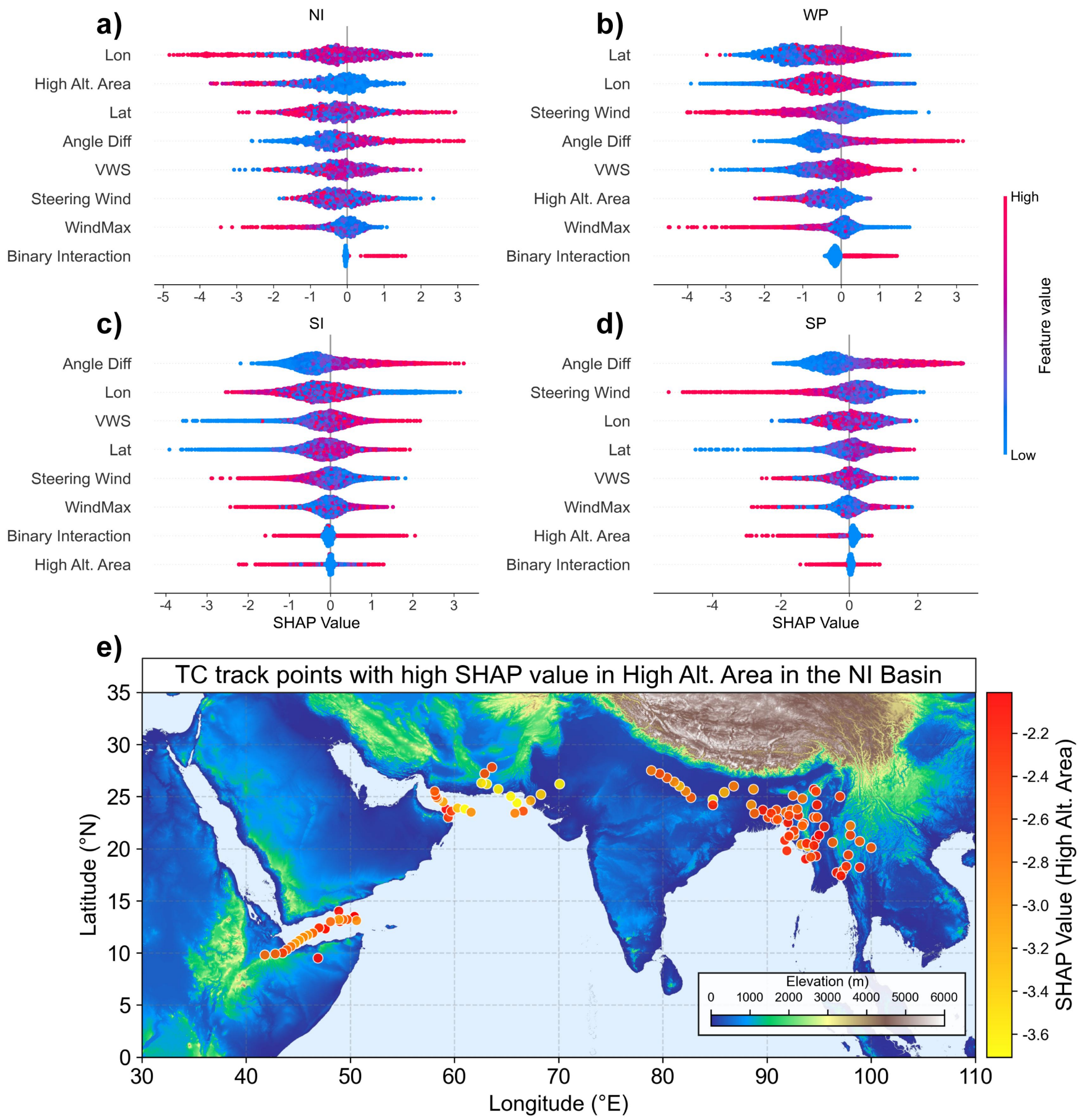

Supplementary Fig. 19 | SHapley Additive exPlanations (SHAP) analysis from the XGBoost model for tropical cyclone (TC) stalling in the land nearshore regions. Beeswarm summary plots (**a - d**) based on the XGBoost model for four basins and TC track points with high SHAP value in the area of high-altitude terrain (High Alt. Area) in the NI Basin (**c - g**). Beeswarm summary plots of basins with less than 10 TC track points in land and nearshore areas are not shown. Track points in panel **e** are characterized by high topographic exposure (High Alt. Area > mean) and strongly negative contributions (SHAP value <-2), and they are also non-stalling. The NI, WP, SI, and SP represent the North Indian, Western North Pacific, South Indian, and South Pacific, respectively.

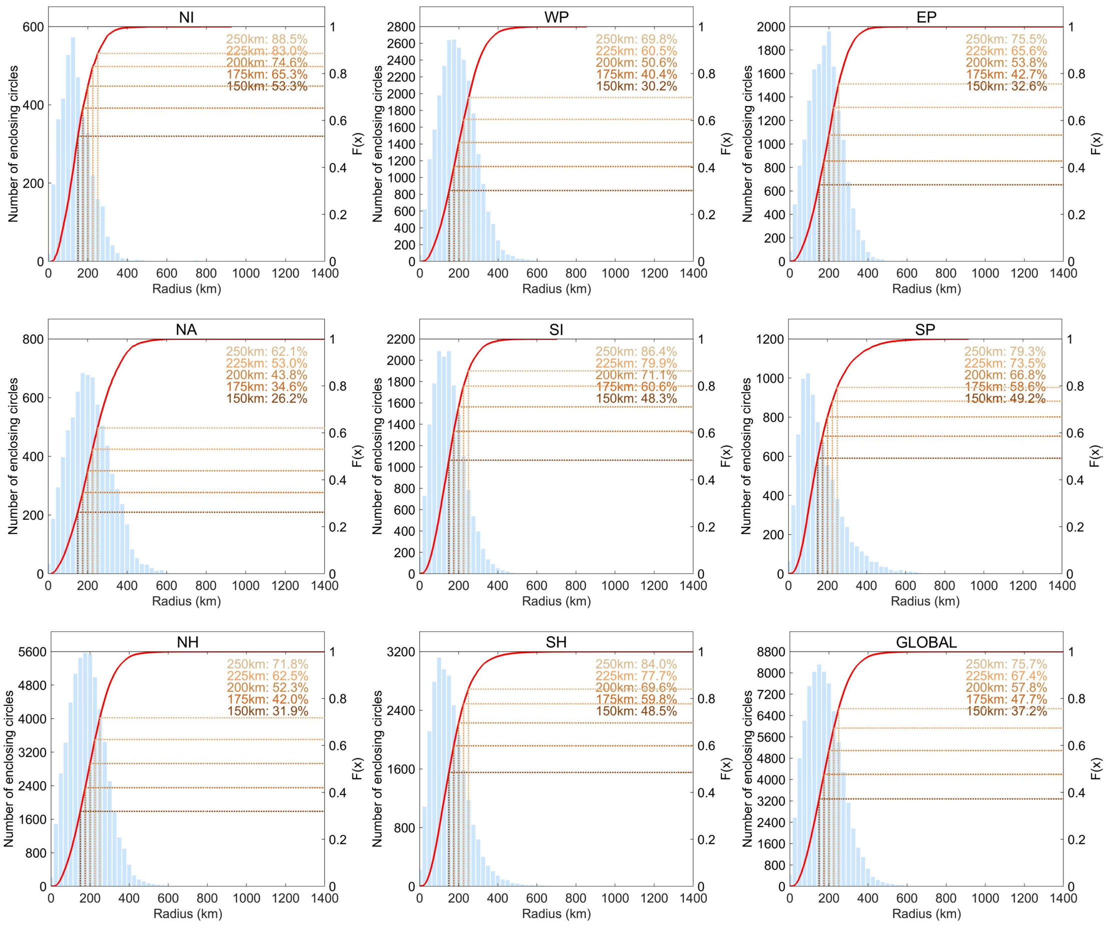

Supplementary Fig. 20 | Histograms and the cumulative density function of the minimum enclosing circle radius with 24-hr stalled time across all the basins during 1982-2019 period. The percentiles of minimum enclosing circle radius at 150, 175, 200, 225, 250 km in this distribution are marked.

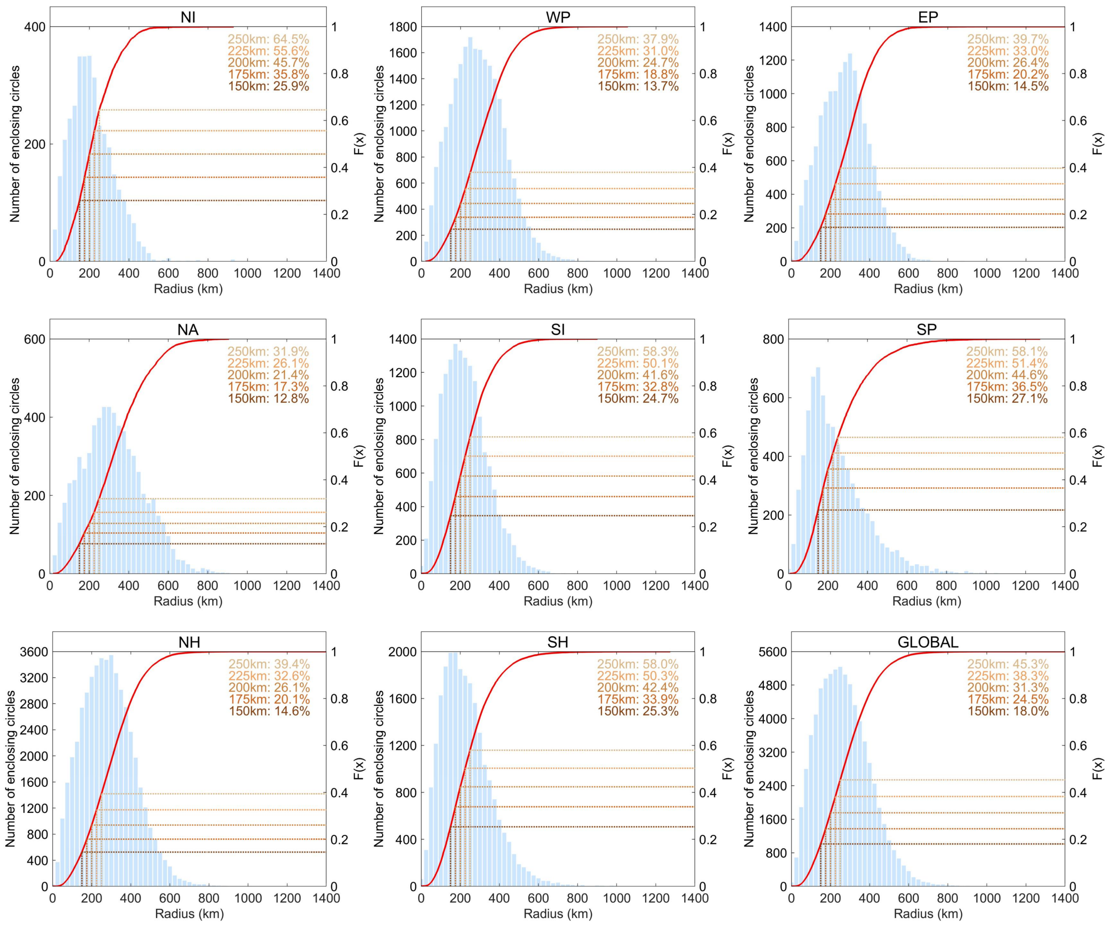

Supplementary Fig. 21 | Histograms and the cumulative density function of the minimum enclosing circle radius with 36-hr stalled time across all the basins during 1982-2019 period. The percentiles of minimum enclosing circle radius at 150, 175, 200, 225, 250 km in this distribution are marked.

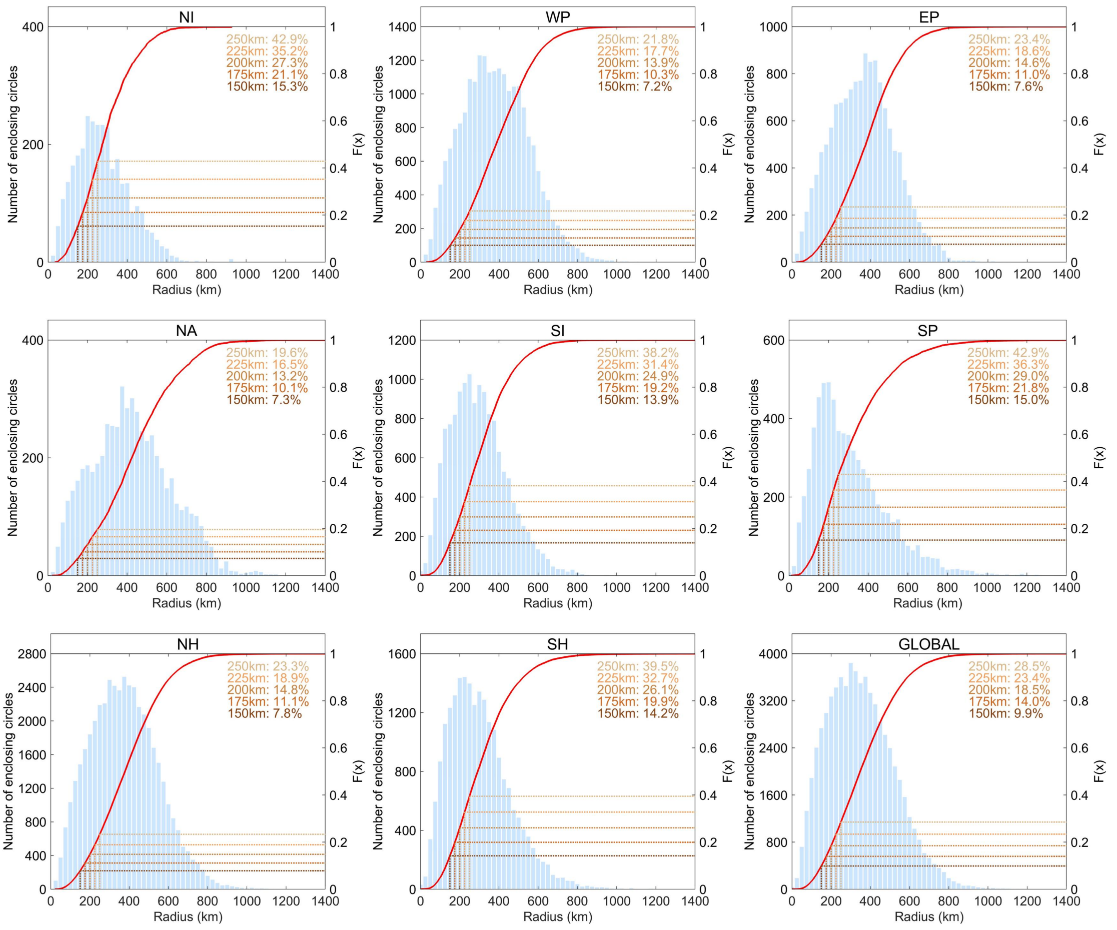

Supplementary Fig. 22 | Histograms and the cumulative density function of the minimum enclosing circle radius with 48-hr stalled time across all the basins during 1982-2019 period. The percentiles of minimum enclosing circle radius at 150, 175, 200, 225, 250 km in this distribution are marked.

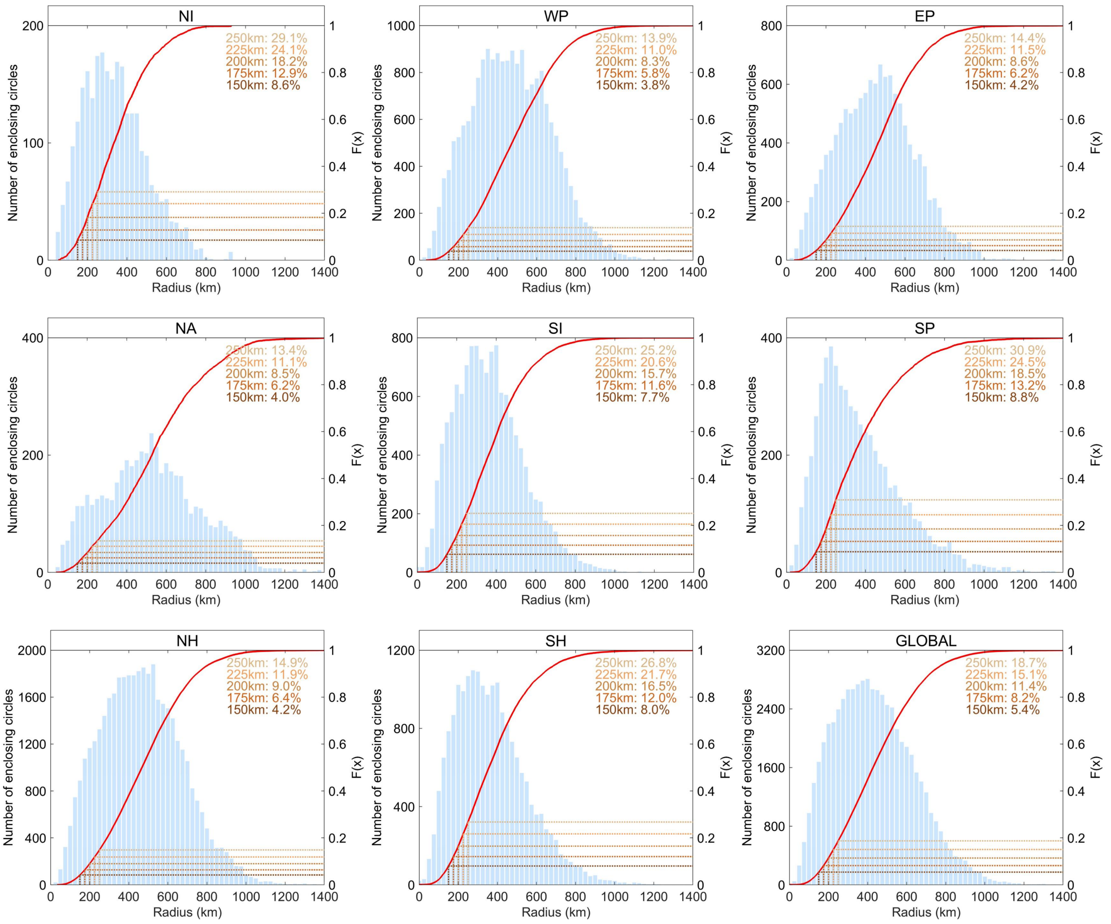

Supplementary Fig. 23 | Histograms and the cumulative density function of the minimum enclosing circle radius with 60-hr stalled time across all the basins during 1982-2019 period. The percentiles of minimum enclosing circle radius at 150, 175, 200, 225, 250 km in this distribution are marked.

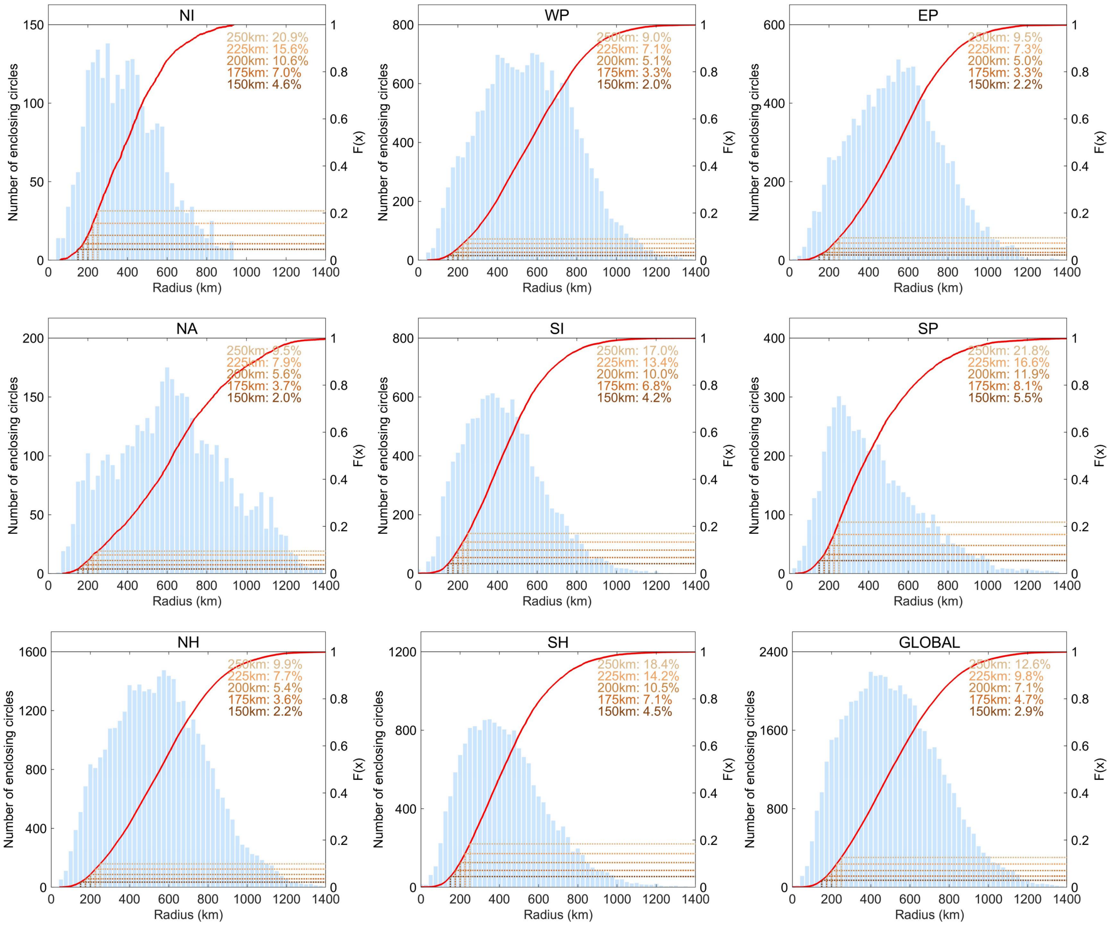

Supplementary Fig. 24 | Histograms and the cumulative density function of the minimum enclosing circle radius with 72-hr stalled time across all the basins during 1982-2019 period. The percentiles of minimum enclosing circle radius at 150, 175, 200, 225, 250 km in this distribution are marked.

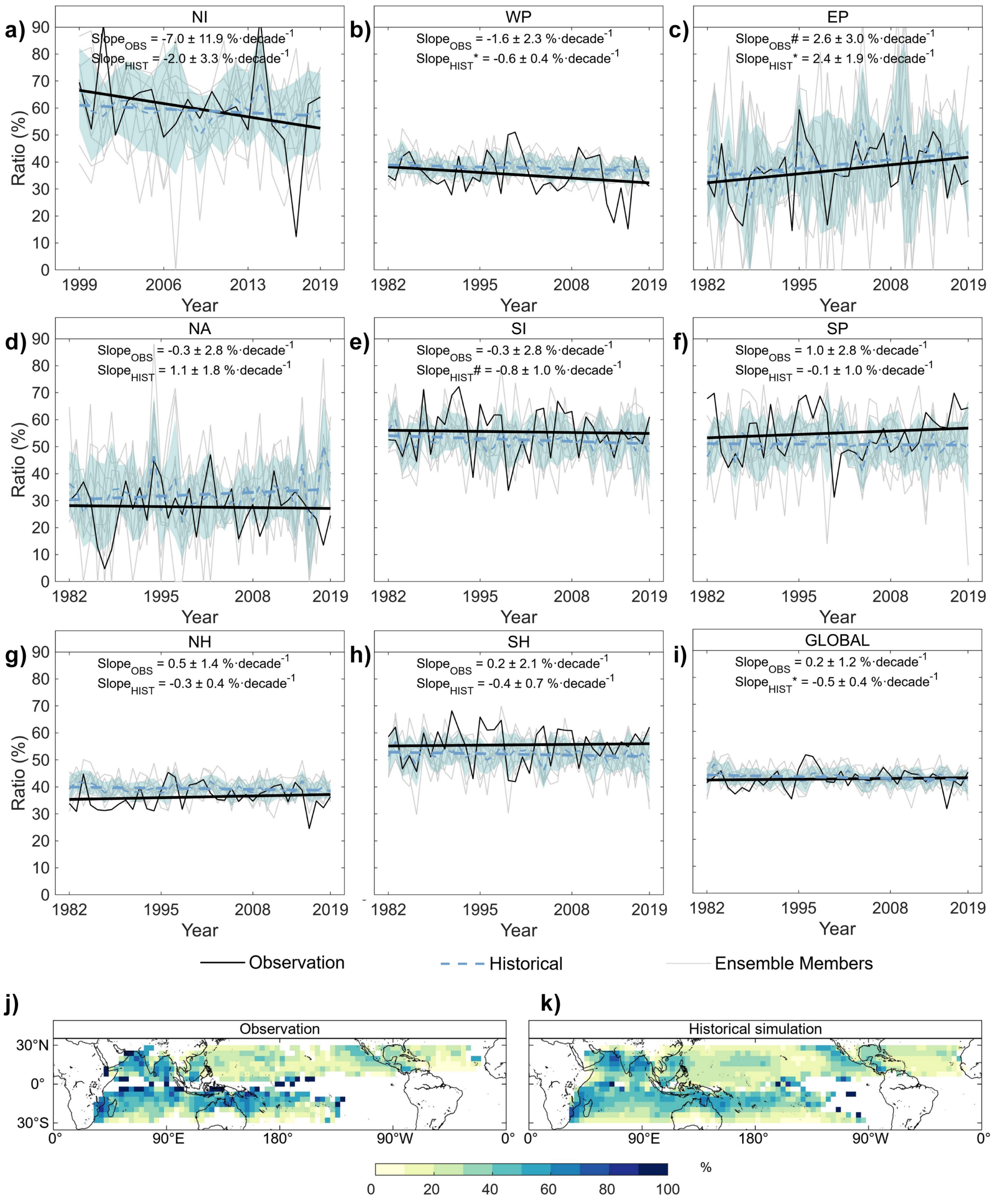

Supplementary Fig. 25 | Time series and spatial distribution of observed and simulated tropical cyclone (TC) stalling ratios under the definition with a 36-hour stalled time and a 200-km enclosing circle radius. The time series of simulated TC stalling ratios (blue dash line) are the average of ten members in the simulated results, with the shading denoting one standard deviation of the member results (**a - i**). The observed and simulated trends are estimated by ordinary least squares method. The symbols "*" and "#" indicate that the trend line has a statistically significant slope at the 5% and 10% level, respectively. Spatial pattern of the ratio of frequency in TC stalling ratio are obtained from observations (**j**) and simulation of "Historical" scenario (**k**). Frequency results in panel **j - k** are shown on grids with a spatial resolution of 4°. These results are based on a TC stalling definition, which specifies that a TC is considered stalled when it remains within a 200-km radius for more than 36 hours.

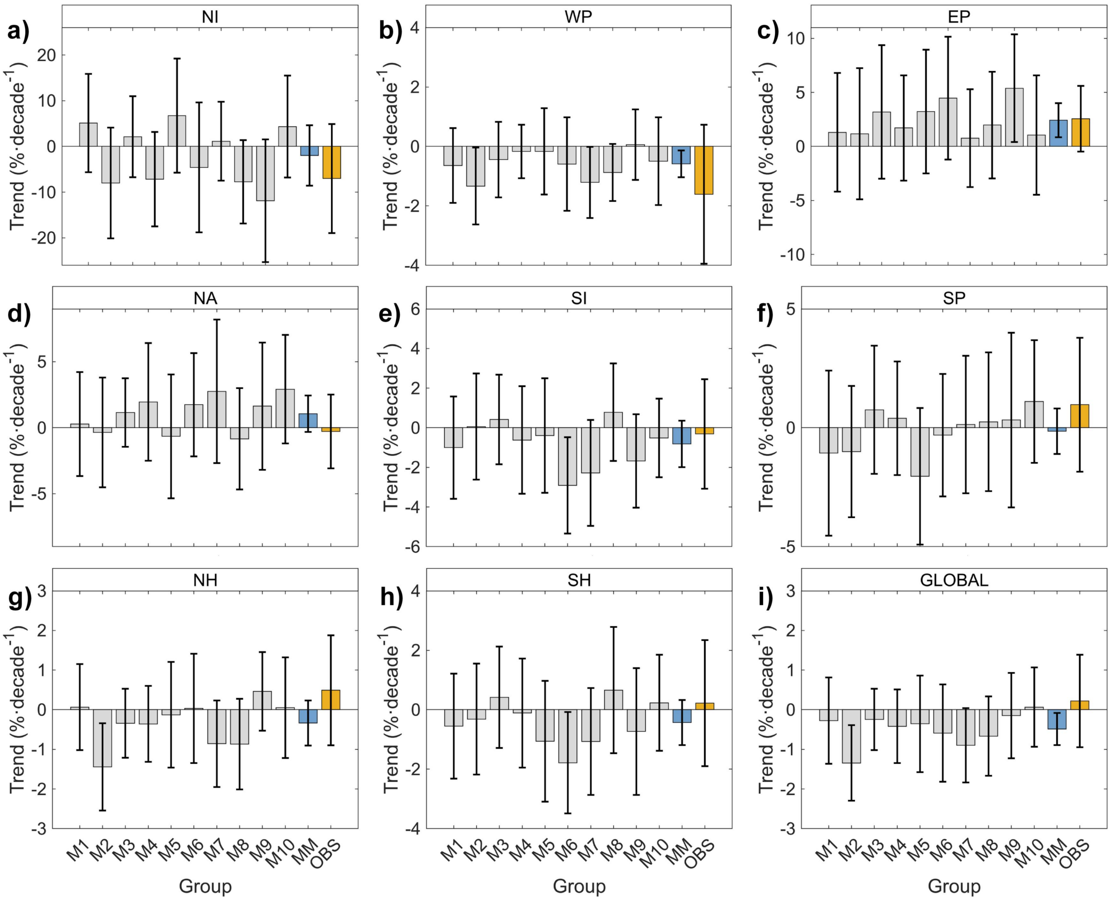

Supplementary Fig. 26 | Trends of time series of observed and simulated tropical cyclone (TC) stalling ratios under the definition with a 36-hour stalled time and a 200-km enclosing circle radius. The trends of the simulated TC stalling ratios for the ten members of the "Historical" scenario are shown in gray bars, while the trends of their ensemble mean time series are shown as blue bars. The trends in the observed TC stalling ratios are shown in yellow bars. Error bars are the 95% confidence intervals for these trends. "M1"-"M10" represent the results of members one to ten of the "Historical" scenario, respectively. "MM" represents the results of the ensemble mean of the simulations of ten members. "OBS" represents the results of the observations. The NI, WP, EP, NA, SI, SP, NH, and SH represent the North Indian, Western North Pacific, Eastern North Pacific, North Atlantic, South Indian, South Pacific, North Hemisphere, and South Hemisphere, respectively. These results are based on a TC stalling definition, which specifies that a TC is considered stalled when it remains within a 200-km radius for more than 36 hours.

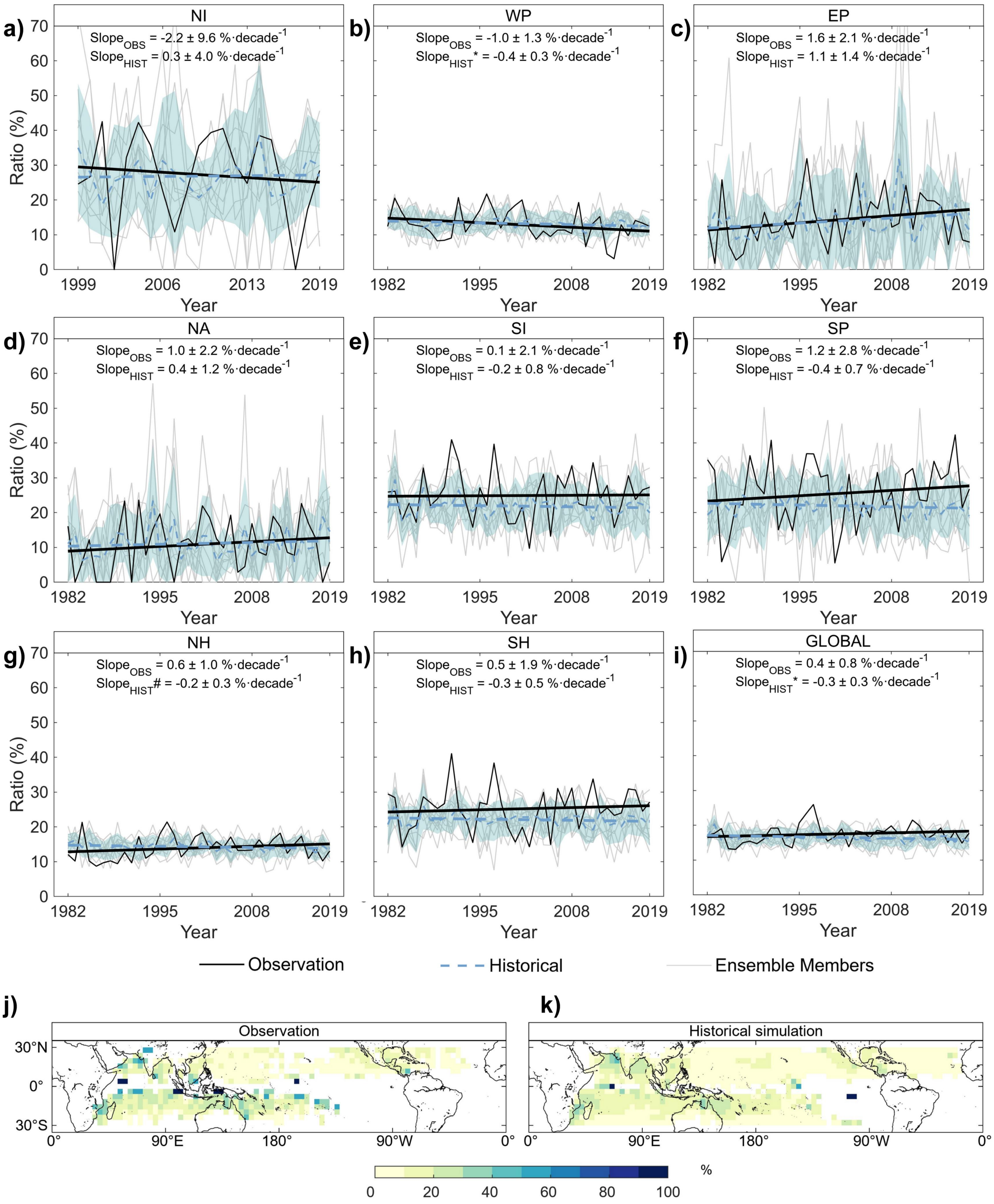

Supplementary Fig. 27 | Time series and spatial distribution of observed and simulated tropical cyclone (TC) stalling ratios under the definition with a 48-hour stalled time and a 150-km enclosing circle radius. The time series of simulated TC stalling ratios (blue dash line) are the average of ten members in the simulated results, with the shading denoting one standard deviation of the member results (**a - i**). The observed and simulated trends are estimated by ordinary least squares method. The symbols "*" and "#" indicate that the trend line has a statistically significant slope at the 5% and 10% level, respectively. Spatial pattern of the ratio of frequency in TC stalling ratio are obtained from observations (**j**) and simulation of "Historical" scenario (**k**). Frequency results in panel **j - k** are shown on grids with a spatial resolution of 4°. These results are based on a TC stalling definition, which specifies that a TC is considered stalled when it remains within a 150-km radius for more than 48 hours.

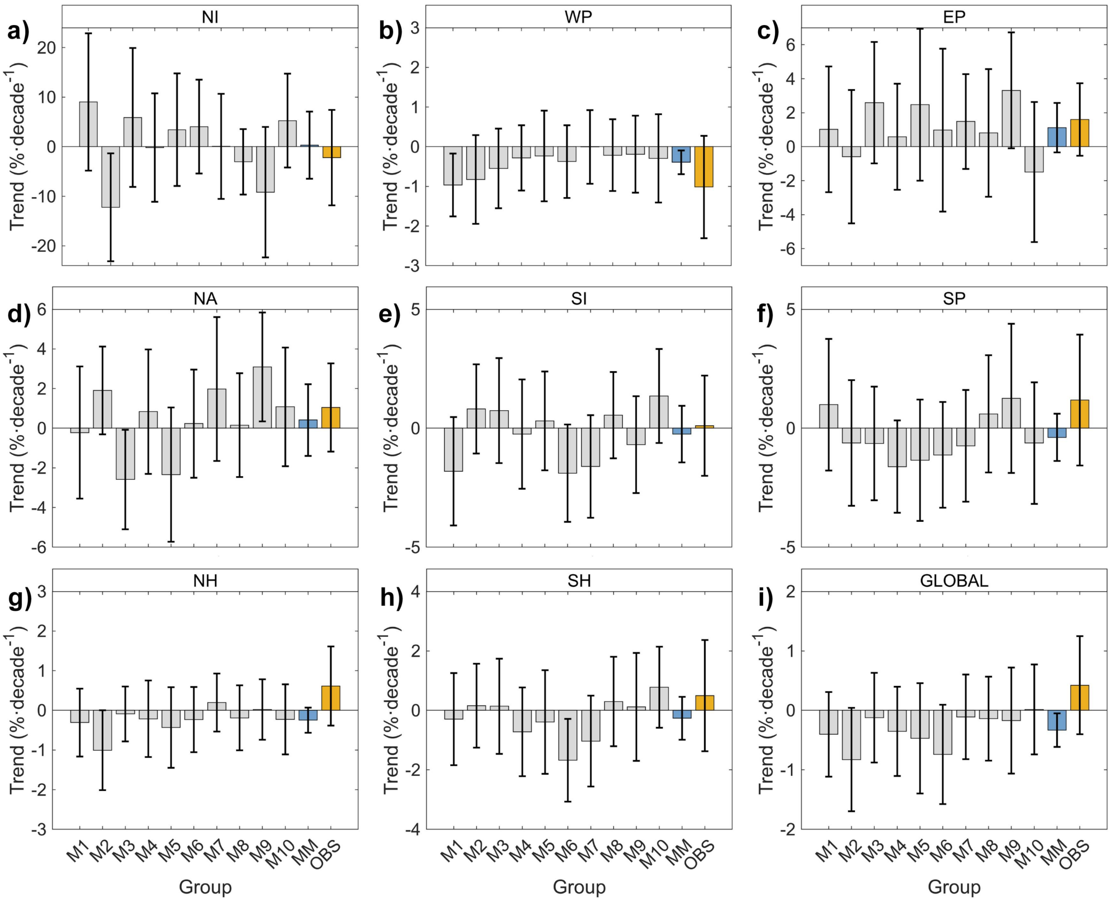

Supplementary Fig. 28 | Trends of time series of observed and simulated tropical cyclone (TC) stalling ratios under the definition with a 48-hour stalled time and a 150-km enclosing circle radius. The trends of the simulated TC stalling ratios for the ten members of the "Historical" scenario are shown in gray bars, while the trends of their ensemble mean time series are shown as blue bars. The trends in the observed TC stalling ratios are shown in yellow bars. Error bars are the 95% confidence intervals for these trends. "M1"-"M10" represent the results of members one to ten of the "Historical" scenario, respectively. "MM" represents the results of the ensemble mean of the simulations of ten members. "OBS" represents the results of the observations. The NI, WP, EP, NA, SI, SP, NH, and SH represent the North Indian, Western North Pacific, Eastern North Pacific, North Atlantic, South Indian, South Pacific, North Hemisphere, and South Hemisphere, respectively. These results are based on a TC stalling definition, which specifies that a TC is considered stalled when it remains within a 150-km radius for more than 48 hours.

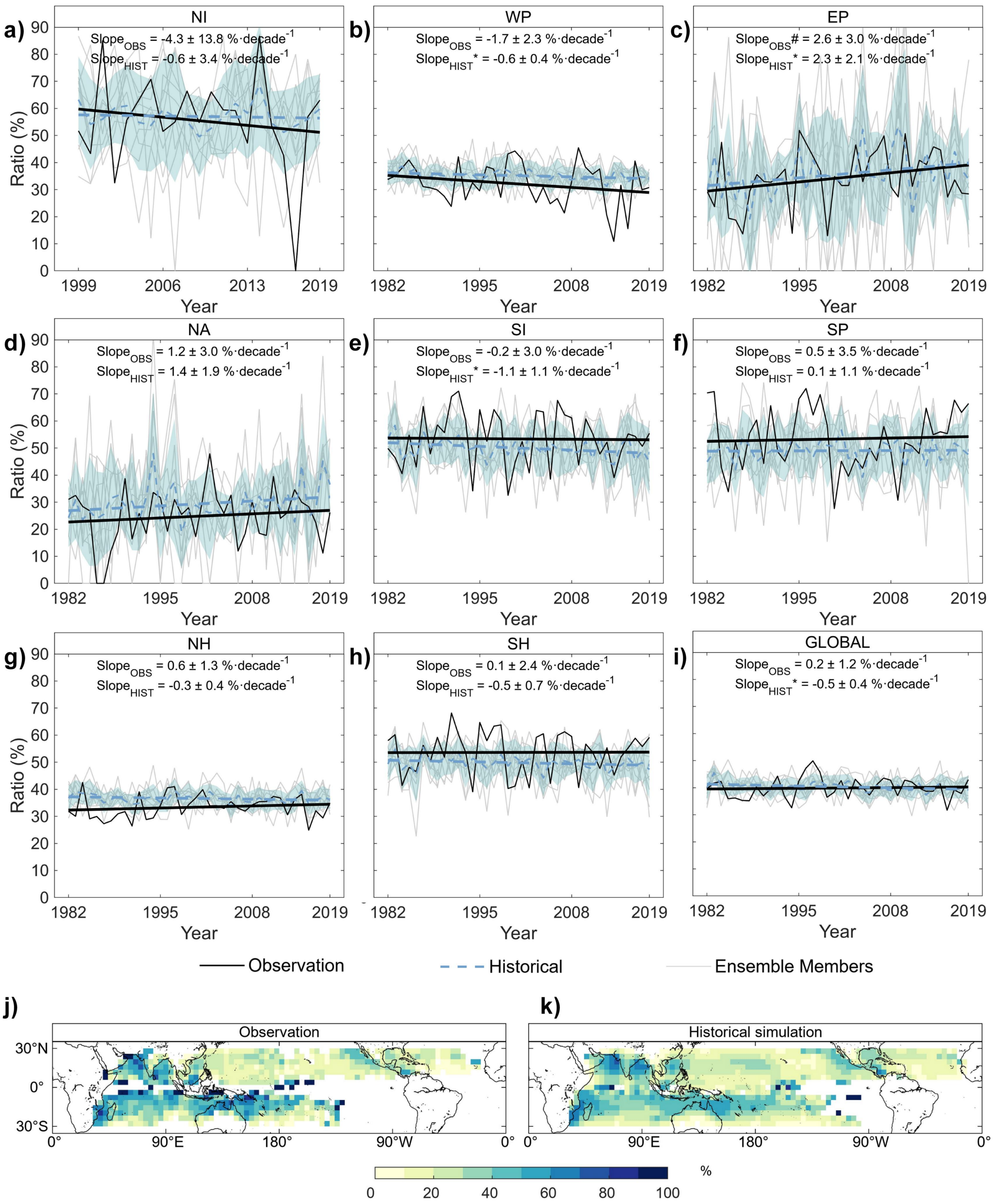

Supplementary Fig. 29 | Time series and spatial distribution of observed and simulated tropical cyclone (TC) stalling ratios under the definition with a 48-hour stalled time and a 250-km enclosing circle radius. The time series of simulated TC stalling ratios (blue dash line) are the average of ten members in the simulated results, with the shading denoting one standard deviation of the member results (**a - i**). The observed and simulated trends are estimated by ordinary least squares method. The symbols "*" and "#" indicate that the trend line has a statistically significant slope at the 5% and 10% level, respectively. Spatial pattern of the ratio of frequency in TC stalling ratio are obtained from observations (**j**) and simulation of "Historical" scenario (**k**). Frequency results in panel **j - k** are shown on grids with a spatial resolution of 4°. These results are based on a TC stalling definition, which specifies that a TC is considered stalled when it remains within a 250-km radius for more than 48 hours.

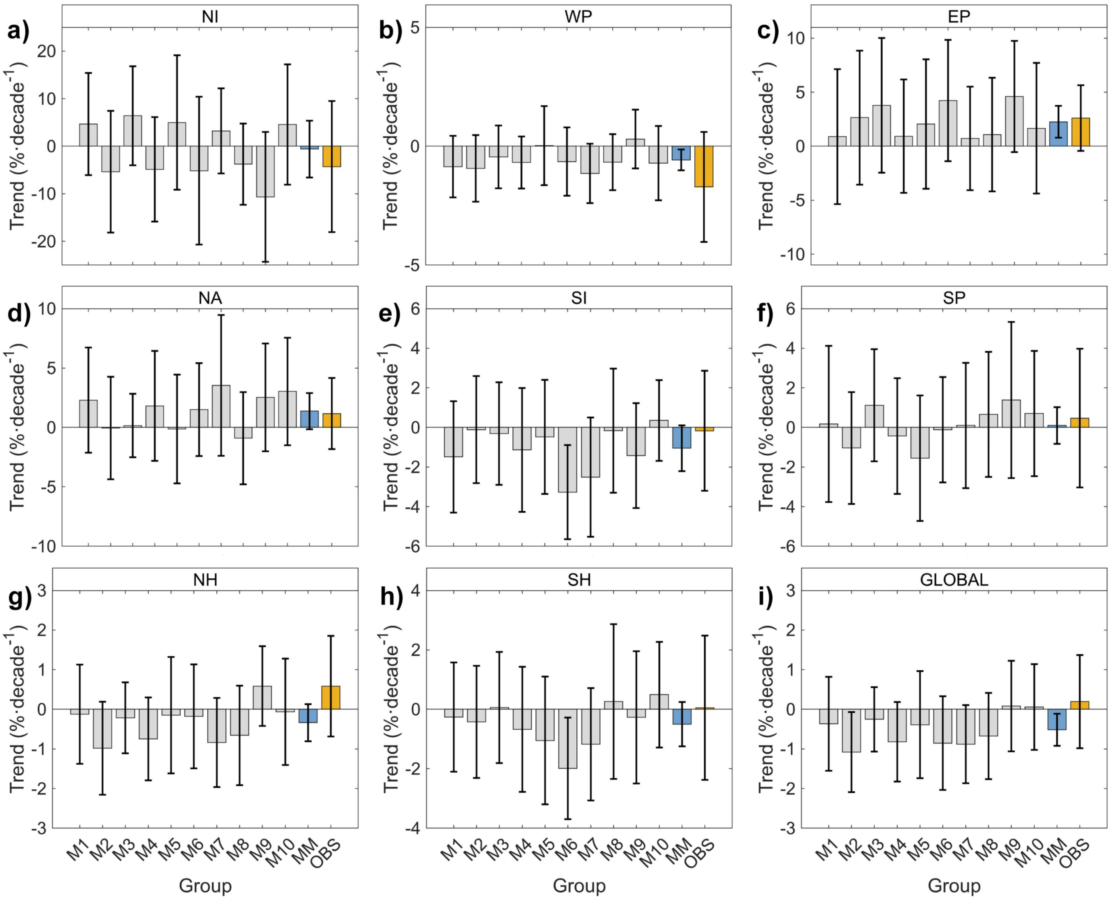

Supplementary Fig. 30 | Trends of time series of observed and simulated tropical cyclone (TC) stalling ratios under the definition with a 48-hour stalled time and a 250-km enclosing circle radius. The trends of the simulated TC stalling ratios for the ten members of the "Historical" scenario are shown in gray bars, while the trends of their ensemble mean time series are shown as blue bars. The trends in the observed TC stalling ratios are shown in yellow bars. Error bars are the 95% confidence intervals for these trends. "M1"-"M10" represent the results of members one to ten of the "Historical" scenario, respectively. "MM" represents the results of the ensemble mean of the simulations of ten members. "OBS" represents the results of the observations. The NI, WP, EP, NA, SI, SP, NH, and SH represent the North Indian, Western North Pacific, Eastern North Pacific, North Atlantic, South Indian, South Pacific, North Hemisphere, and South Hemisphere, respectively. These results are based on a TC stalling definition, which specifies that a TC is considered stalled when it remains within a 250-km radius for more than 48 hours.

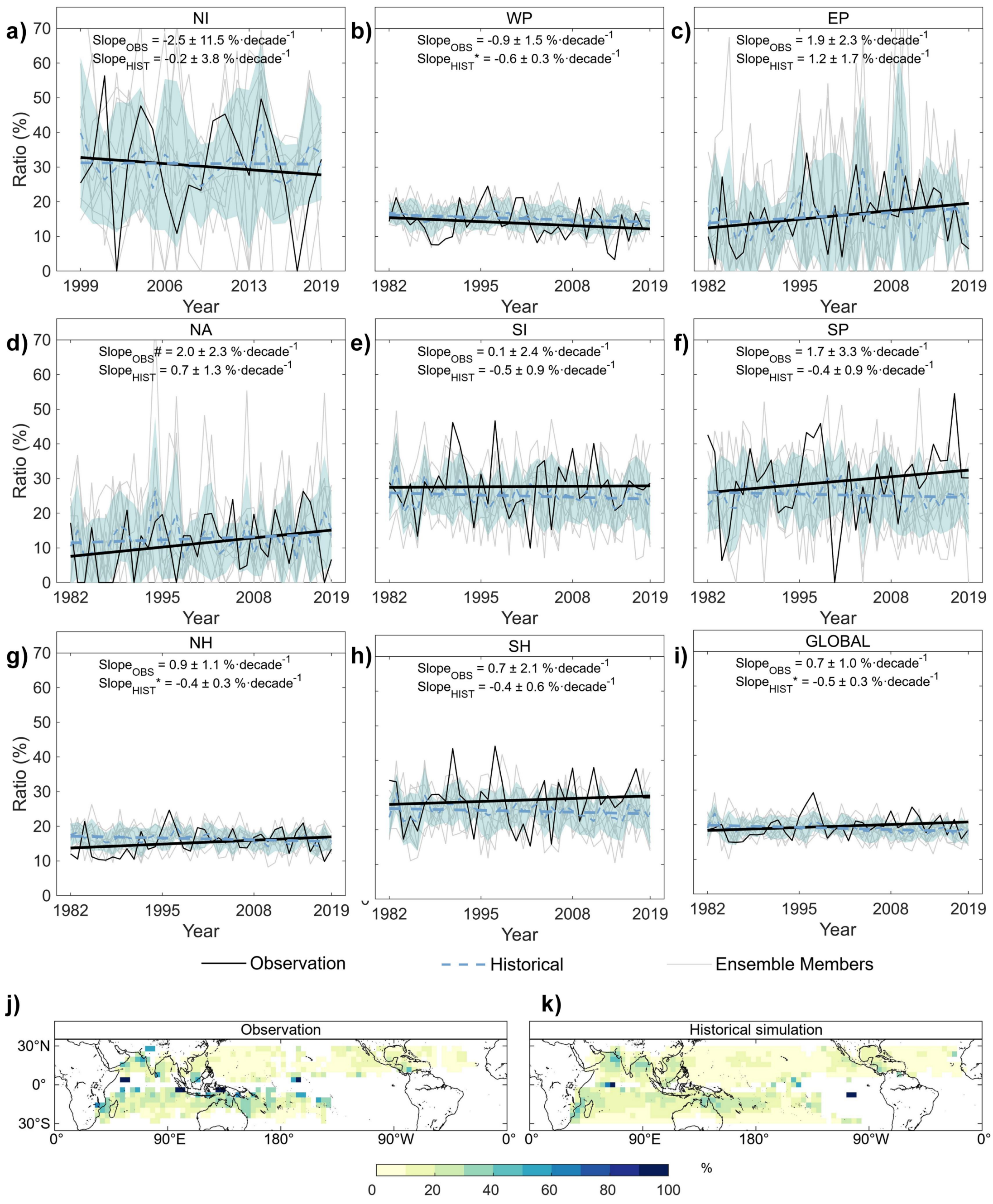

Supplementary Fig. 31 | Time series and spatial distribution of observed and simulated tropical cyclone (TC) stalling ratios under the definition with a 60-hour stalled time and a 200-km enclosing circle radius. The time series of simulated TC stalling ratios (blue dash line) are the average of ten members in the simulated results, with the shading denoting one standard deviation of the member results (**a - i**). The observed and simulated trends are estimated by ordinary least squares method. The symbols "*" and "#" indicate that the trend line has a statistically significant slope at the 5% and 10% level, respectively. Spatial pattern of the ratio of frequency in TC stalling ratio are obtained from observations (**j**) and simulation of "Historical" scenario (**k**). Frequency results in panel **j - k** are shown on grids with a spatial resolution of 4°. These results are based on a TC stalling definition, which specifies that a TC is considered stalled when it remains within a 200-km radius for more than 60 hours.

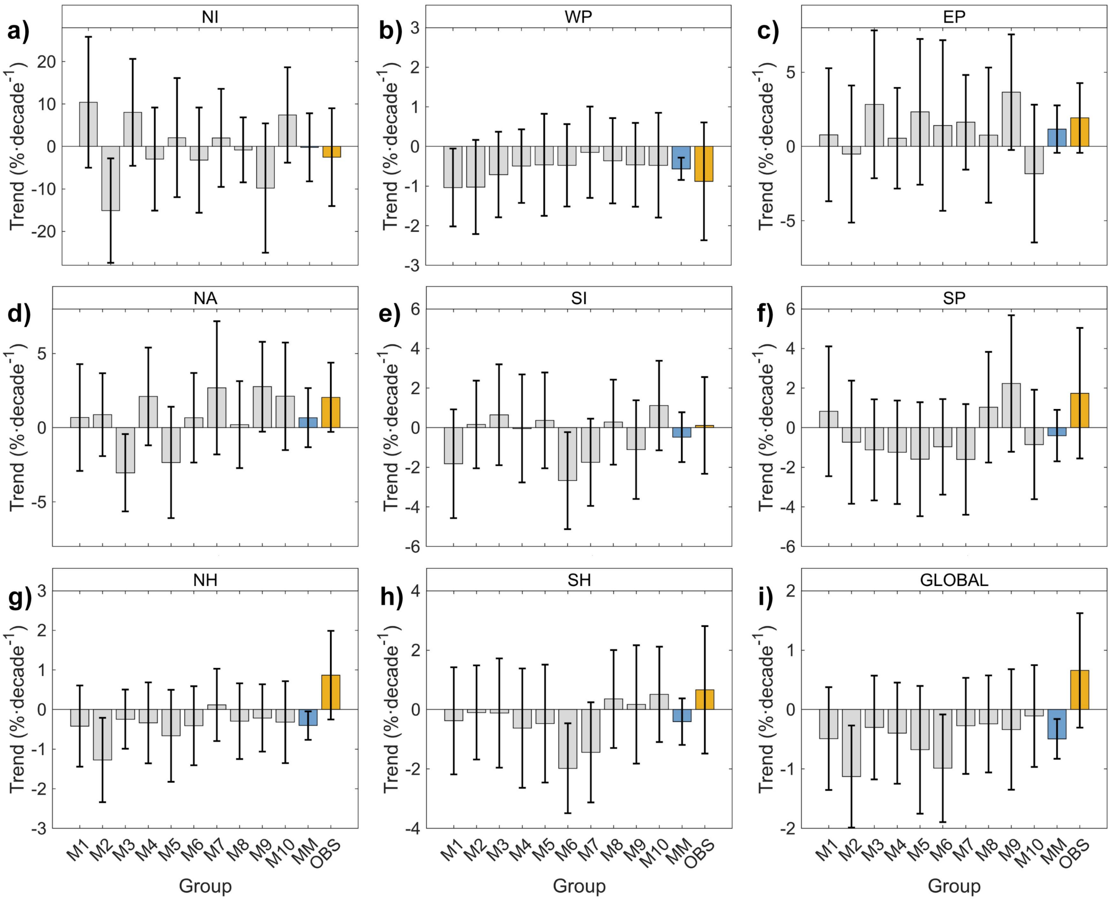

Supplementary Fig. 32 | Trends of time series of observed and simulated tropical cyclone (TC) stalling ratios under the definition with a 60-hour stalled time and a 200-km enclosing circle radius. The trends of the simulated TC stalling ratios for the ten members of the "Historical" scenario are shown in gray bars, while the trends of their ensemble mean time series are shown as blue bars. The trends in the observed TC stalling ratios are shown in yellow bars. Error bars are the 95% confidence intervals for these trends. "M1"-"M10" represent the results of members one to ten of the "Historical" scenario, respectively. "MM" represents the results of the ensemble mean of the simulations of ten members. "OBS" represents the results of the observations. The NI, WP, EP, NA, SI, SP, NH, and SH represent the North Indian, Western North Pacific, Eastern North Pacific, North Atlantic, South Indian, South Pacific, North Hemisphere, and South Hemisphere, respectively. These results are based on a TC stalling definition, which specifies that a TC is considered stalled when it remains within a 200-km radius for more than 60 hours.

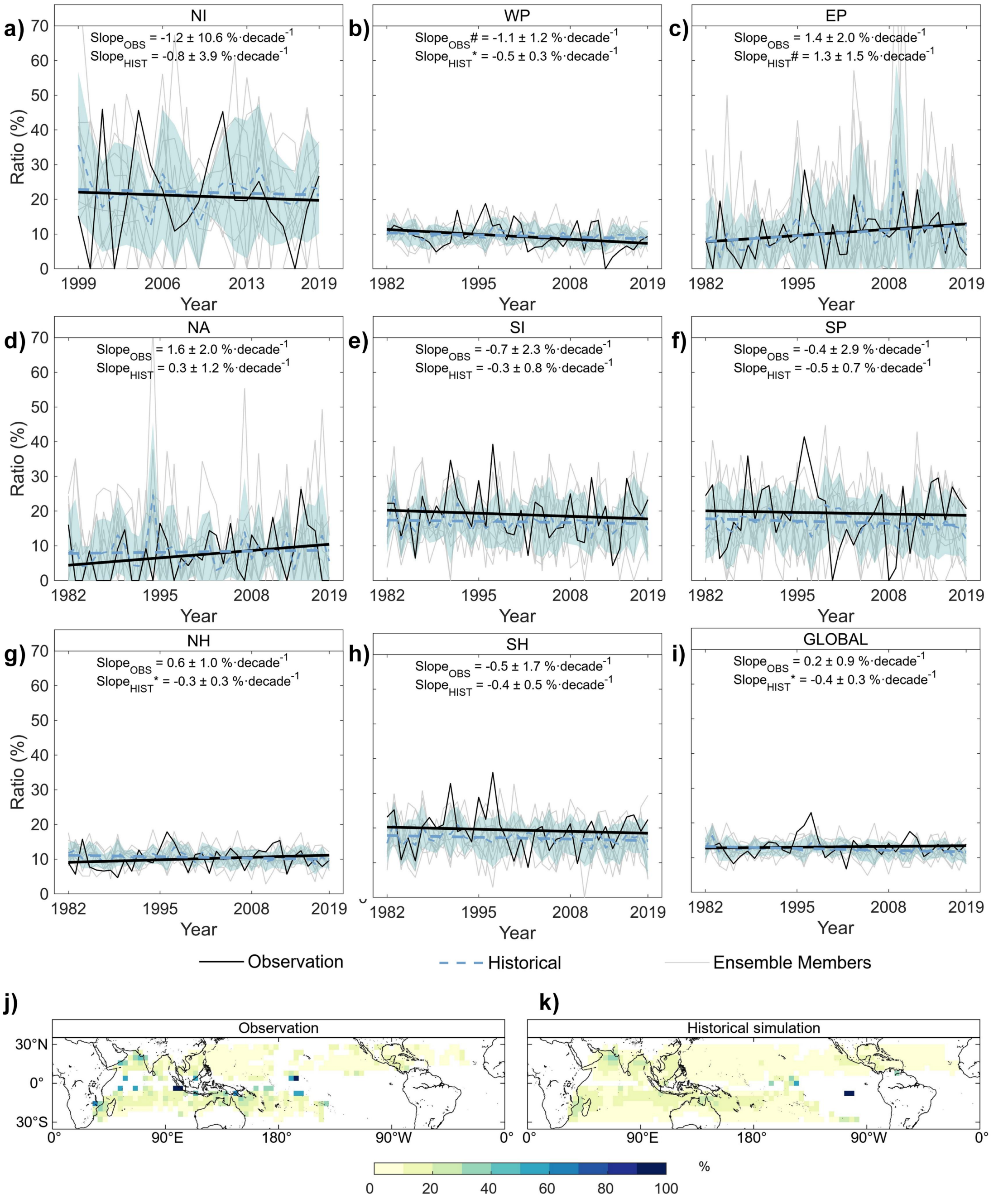

Supplementary Fig. 33 | Time series and spatial distribution of observed and simulated tropical cyclone (TC) stalling ratios under the definition with a 72-hour stalled time and a 200-km enclosing circle radius. The time series of simulated TC stalling ratios (blue dash line) are the average of ten members in the simulated results, with the shading denoting one standard deviation of the member results (**a - i**). The observed and simulated trends are estimated by ordinary least squares method. The symbols “*” and “#” indicate that the trend line has a statistically significant slope at the 5% and 10% level, respectively. Spatial pattern of the ratio of frequency in TC stalling ratio are obtained from observations (**j**) and simulation of “Historical” scenario (**k**). Frequency results in panel **j - k** are shown on grids with a spatial resolution of 4°. These results are based on a TC stalling definition, which specifies that a TC is considered stalled when it remains within a 200-km radius for more than 72 hours.

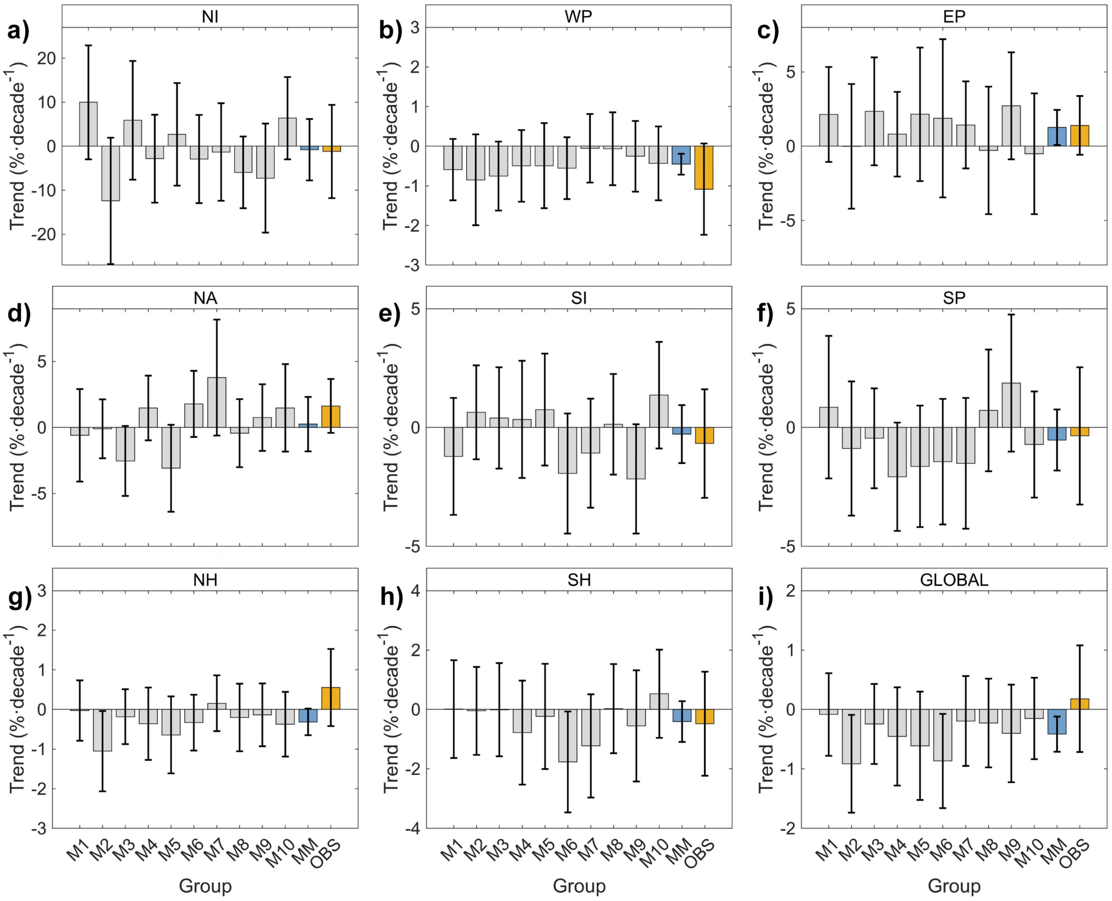

Supplementary Fig. 34 | Trends of time series of observed and simulated tropical cyclone (TC) stalling ratios under the definition with a 72-hour stalled time and a 200-km enclosing circle radius. The trends of the simulated TC stalling ratios for the ten members of the "Historical" scenario are shown in gray bars, while the trends of their ensemble mean time series are shown as blue bars. The trends in the observed TC stalling ratios are shown in yellow bars. Error bars are the 95% confidence intervals for these trends. "M1"-"M10" represent the results of members one to ten of the "Historical" scenario, respectively. "MM" represents the results of the ensemble mean of the simulations of ten members. "OBS" represents the results of the observations. The NI, WP, EP, NA, SI, SP, NH, and SH represent the North Indian, Western North Pacific, Eastern North Pacific, North Atlantic, South Indian, South Pacific, North Hemisphere, and South Hemisphere, respectively. These results are based on a TC stalling definition, which specifies that a TC is considered stalled when it remains within a 200-km radius for more than 72 hours.

Basin	Jan.	Feb.	Mar.	Apr.	May	Jun.	Jul.	Aug.	Sep.	Oct.	Nov.	Dec.	Total
NI	41.0	43.3	53.6	37.2	32.8	50.6	56.8	0	36.5	42.6	32.0	32.1	38.2
WP	18.7	30.9	21.4	31.3	25.9	16.1	14.3	25.3	23.8	24.2	24.9	19.4	23.0
EP	35.4	0	100	0	44.8	34.8	7.8	11.9	30.9	35.7	41.8	52.8	33.0
NA	34.9	0	0	52.2	9.3	24.2	15.4	7.6	17.0	31.4	37.1	33.8	21.9
SI	39.1	37.7	36.4	36.8	52.8	25.8	48.8	36.5	33.9	43.2	42.7	39.3	39.4
SP	41.3	35.0	43.3	36.9	51.4	60.0	93.3	75.0	47.6	77.4	52.8	43.5	54.8
NH	25.1	31.0	24.2	32.7	31.3	28.4	11.4	17.0	24.5	30.0	29.3	24.3	25.8
SH	39.6	36.6	39.0	36.7	52.0	45.6	52.6	48.7	35.4	50.7	45.5	40.3	43.6
GLOBAL	37.9	36.3	37.6	35.4	36.3	29.0	12.2	17.2	24.6	31.2	33.3	33.9	30.4

Supplementary Table 1 | The tropical cyclone stalling ratio (%) in each basin from observation data.

Basin	Area Under Curve .	classification accuracy
NI	0.872 ± 0.007	0.826 ± 0.012
WP	0.890 ± 0.006	0.839 ± 0.009
EP	0.935 ± 0.007	0.890 ± 0.009
NA	0.836 ± 0.009	0.758 ± 0.009
SI	0.880 ± 0.010	0.792 ± 0.011
SP	0.908 ± 0.011	0.834 ± 0.014

Supplementary Table 2 | The performance of basin-specific XGBoost models.

Basin	Stalling-inducing intervals		
	Steering wind (m/s)	Angle Diff (°)	VWS (m/s)
NI	[0, 2.65)	(50.85, 180]	(12.82, 22.98)
WP	[0, 3.51)	(44.83, 180]	(13.67, 28.20)
EP	[0, 3.06)	(39.31, 180]	(11.04, 28.08)
NA	[0, 3.59)	(34.70, 180]	(11.60, 27.28)
SI	[0, 2.87)	(45.57, 180]	(13.09, 21.61)
SP	[0, 3.37)	(38.95, 180]	(8.01, 20.61)

Supplementary Table 3 | Critical Environmental Thresholds and stalling-inducing intervals for Each Basin.

Basin	TC track points	Probability of having ≥ 1 separate coexisting TCs within ...		
		2000 km	3000 km	4000 km
NI	Stalling (1807)	6.0% (108)	11.9% (215)	14.7% (266)
	Non-stalling (3050)	3.0% (91)	6.4% (196)	8.1% (246)
	Ratio difference	3.0%	5.5%	6.6%
WP	Stalling (6802)	26.5% (1801)	44.6% (3033)	52.1% (3546)
	Non-stalling (23616)	17.7% (4189)	35.1% (8284)	42.2% (9964)
	Ratio difference	8.8%	9.5%	9.9%
EP	Stalling (4496)	28.4% (1278)	39.2% (1764)	42.3% (1927)
	Non-stalling (15310)	25.8% (3945)	41.0% (6283)	47.2% (7233)
	Ratio difference	2.6%	-1.8%	-4.9%
NA	Stalling (1846)	9.3% (172)	17.0% (314)	21.8% (402)
	Non-stalling (7888)	12.1% (955)	21.8% (1722)	28.2% (2223)
	Ratio difference	-2.8%	-4.8%	-6.4%
SI	Stalling (7754)	13.0% (1011)	26.6% (2059)	32.8% (2542)
	Non-stalling (12336)	10.8% (1333)	22.6% (2790)	28.7% (3540)
	Ratio difference	2.2%	4.0%	4.1%
SP	Stalling (4157)	12.5% (519)	25.9% (1076)	30.2% (1254)
	Non-stalling (5851)	14..4% (840)	24.6% (1439)	29.1% (1705)
	Ratio difference	-1.9%	1.3%	1.1%
NH	Stalling (14919)	23.7% (3542)	39.0% (5812)	47.1% (7019)
	Non-stalling (49880)	19.3% (9628)	35.3% (17591)	43.7% (21815)
	Ratio difference	4.4%	3.7%	3.4%
SH	Stalling (11917)	13.4% (1595)	28.9% (3438)	35.1% (4179)
	Non-stalling (18171)	12.1% (2202)	24.3% (4415)	31.2% (5668)
	Ratio difference	1.3%	4.6%	3.9%
GLOBAL	Stalling (26876)	20.0% (5365)	37.5% (10067)	46.0% (12370)
	Non-stalling (68011)	17.8% (12096)	34.6% (23529)	44.1% (30013)
	Ratio difference	2.2%	2.9%	1.9%

Supplementary Table 4 | Probability of having at least one separate coexisting tropical cyclones in the vicinity of stalled and non-stalled tropical cyclones track points in each basin from observation data.
