# Supplementary material for: Global stalled tropical cyclones in a changing climate
Source: Nat Commun. 2026 Mar 30;17:5145. doi: 10.1038/s41467-026-71320-3 (PMC13249938; doi:10.1038/s41467-026-71320-3)
Supplement: Supplementary file 2 — Transparent Peer Review file [file 41467_2026_71320_MOESM2_ESM.pdf]

nature portfolio

Peer Review File

Global stalled tropical cyclones in a changing climate

Corresponding Author: Dr Gabriele Villarini

This file contains all reviewer reports in order by version, followed by all author rebuttals in order by version.

Version 0:

Reviewer comments:

Reviewer #1

(Remarks to the Author)
This manuscript investigates and analyzes the special behavior of tropical cyclone (TC) stalling at the global scale, presenting the climatology and trends under climate change. The topic is interesting, and the overall flow of the manuscript is clear. However, there are several key issues that prevent me from providing more positive feedback, particularly concerning the reliability of global circulation models (GCMs) in analyzing TC stalling. My detailed comments are provided below, which I hope will be useful to the authors.

Major Comments
1. Reliability of GCMs for TC stalling analysis
My main concern lies in the reliability of using GCMs to analyze TC stalling. Even with a high-resolution model such as AM2.5C360 (25 km) from Princeton, the resolution is still insufficient to fully capture TC structures. Moreover, most stalling events are characterized by abrupt changes in TC trajectories, which GCMs are generally poor at simulating. I strongly recommend that the authors carefully address this issue by presenting sufficient cases from both observations and simulations to justify the validity of using GCMs for stalling studies.
2. Definition of TC stalling
The definition of TC stalling should be clearly and explicitly stated in the manuscript. Although Hall & Kossin (2019) and Zhang et al. (2024) adopted a similar definition, their studies were conducted mainly in the Northern Hemisphere. The authors here report more frequent stalling in the Southern Hemisphere, likely due to slower translation speeds (Kossin et al., 2019). If the definition is based on the relative proportion of slow-moving TCs, then the threshold may need to be adjusted for different basins. Before proceeding with the analysis, the authors should first discuss the robustness and applicability of their definition across regions.
3. Unclear changes in the Western Pacific (Figure 4)
In Figure 4, the changes in the Western Pacific are difficult to interpret as contiguous or robust. Instead, the signal appears scattered and statistically weak, with many isolated spots on the map. The authors should carefully check the statistical significance of these results to ensure their robustness.
4. Novelty relative to previous work
Although this manuscript claims to be a global study of TC stalling, much of the analysis appears similar to Zhang et al. (2024). For instance, Figure 1 largely reproduces results already reported in that study. Rather than repeating known results, the authors should emphasize the differences among basins, highlight potential mechanisms underlying stalling in different regions, and provide a more systematic analysis of the environmental changes that influence stalling globally. This comparative and mechanistic focus would strengthen the novelty of the work.

Minor Comments
Line 74: I suggest adopting a threshold based on distance (e.g., kilometers), rather than degrees, as in Hall & Kossin (2019), to avoid the influence of varying latitudes on the degree measure.
Line 82: The use of “although” is not appropriate in this sentence; please reconsider the logic.
Lines 85–86: The number of stalled TC points is less meaningful; the ratio of stalled cases to total TCs is more informative.
Lines 215–216: The causes of stalling should be discussed at this point, rather than left implicit.

Kossin, J.P. A global slowdown of tropical-cyclone translation speed. Nature 558, 104–107 (2018). https://doi.org/10.1038/s41586-018-0158-3
Hall, T.M., Kossin, J.P. Hurricane stalling along the North American coast and implications for rainfall. npj Clim Atmos Sci 2, 17 (2019). https://doi.org/10.1038/s41612-019-0074-8

Zhang, L., Cheng, T. F., Lu, M., Xiong, R., & Gan, J. (2023). Tropical cyclone stalling shifts northward and brings increasing flood risks to East Asian Coast. Geophysical Research Letters, 50(10), e2022GL102509.

(Remarks on code availability)
The code is comprehensive, but the explanation is limited.

Reviewer #2

(Remarks to the Author)
This manuscript focuses on global distribution of stalling storms and the associated rainfall under climate change scenarios. This is a timely and important piece. While some literature exists, this analysis provides an insightful geographic perspective looking at multiple basins and the variation in behavior. The data and methods are extremely well described, and I was left without unanswered questions. They are written in a reproducible way, which readers will greatly appreciate. For verification of availability, I used the Zenodo site and was able to see the available information. In fact, the information has already been downloaded 23 times as of the writing of this review. I think this manuscript is very close to being ready for publication – with only a few line items mentioned below. Thus, I give minor revisions for this manuscript.

Line Items

Line 76: add "global" to the sentence before "…global stalled and nonstalled" – so that it is clear that this figure includes all basins together in the depiction of the variables.
Line 88: It is difficult to discern any difference in Figure 1e related to rainfall intensity – are you using Figure 1f to support this statement? It would be helpful to guide the reader to exactly the panel you wish them to consider when describing your results.
Line 91: could this be due to generally colder ocean temperatures in the eastern Pacific leading to less rainfall?
Line 151: In Trepanier et al 2024, we found near-coastal stalls are increasing in the NA. Include if you think it is relevant. We didn't specifically state it is global warming related, but the data support an increase.
Line 288: Supplementary Figure 8 does not define the basins. I think you mean Figure 1i within the actual manuscript.

(Remarks on code availability)
It is reproducible and usable. I also comment on this in my main review.

Reviewer #3

(Remarks to the Author)
Review of: "Global stalled tropical cyclone in a changing climate" by Deng et al.

Recommendation: Reject

The authors generate a tropical cyclone stalling global climatology and its projected changes under a warming climate. The study combines observations (IBTrACS tracks and MSWEP reanalysis data) with ~25 km GCM simulations (AM2.5C360) in AMIP and historical/future ensemble configuration using a unique scenario (RCP4.5). This work is subdivided into two future periods (Near Future 2022-2059 and Far Future 2062-2099). The main conclusion is that while the global probability of tropical cyclone stalling decreases, the daily rainfall associated with these TCs increases significantly. This precipitation increases are particularly over land and coastal regions, with increases approaching ~30% in North America at the end of the century.

Overall, the manuscript is generally well-written and provides helpful insight in the topic. However, it bears some important flaws which make it of poor quality for this journal: lack of a clear methodology, statistically significant results, the analysis done to reach the findings, and the findings themselves. Detailed comments are listed to help improve the paper, which requires a very high and substantial major revision before it can be considered for publication in a high-quality journal like Nature Communications.

General comments:

1. The main findings in this work are not novel enough to be published in this very high-quality journal, given the number of studies concluding an increase in precipitation in TCs. Along these lines, I do not understand the title, as the main findings and analysis are not related to the stalling itself but to the precipitation. On the other hand, the quality of this work would increase notably if it focused on analyzing TC stalling itself, why it stalls and how it could be reduced in the future (i.e., the main dynamical drivers for this reduction), instead of only studying precipitation related to these TCs.
2. Definition of tropical cyclone stalling, rainfall attribution to TC and their robustness. I have some concerns about the tropical cyclone stalling definition because 200-km could be an arbitrary threshold. It could be interesting to do a sensitivity analysis changing the radius (from 150 to 250, for example) and the minimum duration (from 36 to 60 h). In this line, could be interesting testing other radius in the rainfall attribution due to your 500-km fixed threshold may include environmental/monsoonal rainfall. I suggest to test with other radius (400 to 600 km) and rainfall thresholds (0.3 to 1 mm/6 h; 0.5-2 mm/day).
3. Although the authors used ten ensemble members of the same model, I have some concerns about using a unique future

scenario (RCP4.5) and only one model. Including another scenario and several more models would help to capture a wider range of possible responses and improve confidence in the findings. For instance, the use of HighResMIP models could be advised. Or studying this phenomenon in a coupled climate model as we are dealing with TCs.
4. Dynamical changes. The manuscript needs an insightful discussion of the main dynamical factors that potentially drive the changes in the TC stalling over each basin. The author includes in the manuscript a dynamical attribution which are discussed from other studies. However, this work needs a dynamical analysis to find what are the changes in their simulations. By including this, the quality of this work would increase notably.

(Remarks on code availability)

Reviewer #4

(Remarks to the Author)
I co-reviewed this manuscript with one of the reviewers who provided the listed reports. This is part of the Nature Communications initiative to facilitate training in peer review and to provide appropriate recognition for Early Career Researchers who co-review manuscripts.

(Remarks on code availability)

Version 1:

Reviewer comments:

Reviewer #1

(Remarks to the Author)
Review of ‘Global stalled tropical cyclone in a changing climate’ by Deng et al.

I appreciate the authors’ substantial effort in responding carefully to the reviewers’ comments and their constructive attitude throughout the revision. However, I remain concerned—consistent with my previous review and broadly aligned with Reviewer 3—that the manuscript may still fall short of the novelty and robustness typically expected for a high-impact journal such as Nature Communications. My main concerns are as follows:
1. Model–observation discrepancy and implications for projections.
Based on the newly added Figures S25–S34, the global stalling trends appear to differ substantially between the MMM and the observational estimates. This raises a critical concern about the reliability of the model ensemble for inferring future changes in TC stalling. The manuscript would benefit from a clearer discussion of why these discrepancies arise and how they affect confidence in the projected future trends.
2. Limited conceptual novelty relative to prior work.
In my view, the study primarily extends previous regional stalling analyses to a global scale. While this is a useful synthesis, it is not yet clear that the manuscript offers fundamentally new insights into TC behavior or a distinctly innovative study design beyond geographic expansion. Stronger articulation of the unique scientific contribution (e.g., precipitation features of TC stalling before landfall) would be necessary to justify publication in Nature Communications.
3. Mechanistic understanding remains insufficient despite added ML analyses.
I acknowledge the authors’ additional analyses using XGBoost and SHAP to assess the relative importance of candidate drivers. However, the key limitation in TC stalling research is often the lack of a physically grounded explanation and process-level mechanism, rather than a purely statistical attribution of dominant factors. As currently presented, the machine-learning-based results do not yet translate into a clear physical narrative or testable mechanistic framework (e.g., dynamical pathways linking environmental changes to stalling behavior).
Given these concerns, I suggest the authors consider submission to a strong, more specialized venue such as npj Climate and Atmospheric Science or Communications Earth & Environment, where the manuscript may be better aligned with the journal’s scope and expectations. In my opinion, the work is likely to meet the standards of those journals.

(Remarks on code availability)
The code is well prepared.

Reviewer #3

(Remarks to the Author)
I have reviewed the revised manuscript and am pleased to report that the authors have successfully addressed all of my previous concerns. It is evident that a significant amount of hard work has gone into improving the quality and clarity of the paper following my review. The scientific content is now robust, and the arguments are well-supported. I am happy to recommend this manuscript for publication. However, before final acceptance, there are several minor spelling, grammar, and syntax errors that need to be corrected.

Please see the list of specific technical corrections below:
Page 2, Line 39: The text currently reads: "...one of the costliest No Warming disasters in U.S. history."
Correction: This should be corrected to "natural disasters".
Page 2, Line 44: The text reads: "...potentially associated with low-frequency No Warming variability."
Correction: This should be corrected to "natural variability".
Page 3, Line 71: "Furthermore, changes in probability of TC stalling in climate warming is mainly affected by..."
Correction: It should read: "...changes... are mainly affected by..."
Page 14, Line 388: "Our simulations show that the ratio of TC stalling are declining..."
Correction: It should read: "...the ratio of TC stalling is declining..."
Page 3, Line 118: "...there is artifact in the observations..."
Correction: This should read: "...there is an artifact in the observations..."
Page 13, Figure 5 Caption (Line 337) & Figure 3 Caption: "...based on the absolute frequency of TC stalling at least the 25th percentile..."
Correction: This phrasing is slightly awkward. It should likely read: "...at least at the 25th percentile..." or "...exceeding the 25th percentile..."
Page 14, Line 400: "...the role of this factor on the stalling changes caused by warming is not as much as that of the strength..."
Correction: I suggest changing this to: "...is less significant than that of the strength..."
Page 15, Line 413: "...while others that global TC translation speed would increase..."
Correction: It should read: "...while others suggest/project that global TC translation speed..."
Page 18 Line 525: "...it is similar to what used in a previous study..."
Correction: This should read: "...similar to what was used in a previous study..."
Page 16, Line 439: "...as well as those not in 00:00, 06:00..."
Correction: It should read: "...those not at 00:00, 06:00..."
Page 17, Line 488: "...half ensemble members are forced..."
Correction: This should read: "...half of the ensemble members..."

Once these minor issues are resolved, the manuscript will be in excellent shape. Congratulations to the authors on a very interesting study.

(Remarks on code availability)

Reviewer #4

(Remarks to the Author)
I co-reviewed this manuscript with one of the reviewers who provided the listed reports. This is part of the Nature Communications initiative to facilitate training in peer review and to provide appropriate recognition for Early Career Researchers who co-review manuscripts.

(Remarks on code availability)

Version 2:

Reviewer comments:

Reviewer #1

(Remarks to the Author)
I would like to express my sincere appreciation to the authors for their very careful and thoughtful consideration of my suggestions throughout the two previous rounds of review. I have also learned a great deal from this review process. At this stage, I am convinced that the topic of tropical cyclone stalling warrants a global study such as this one. Therefore, I recommend acceptance of this paper and extend my congratulations to the authors.

(Remarks on code availability)

Open Access This Peer Review File is licensed under a Creative Commons Attribution 4.0 International License, which permits use, sharing, adaptation, distribution and reproduction in any medium or format, as long as you give appropriate credit to the original author(s) and the source, provide a link to the Creative Commons license, and indicate if changes were made.
In cases where reviewers are anonymous, credit should be given to 'Anonymous Referee' and the source.
The images or other third party material in this Peer Review File are included in the article's Creative Commons license, unless indicated otherwise in a credit line to the material. If material is not included in the article's Creative Commons license and your intended use is not permitted by statutory regulation or exceeds the permitted use, you will need to obtain permission directly from the copyright holder.
To view a copy of this license, visit https://creativecommons.org/licenses/by/4.0/

REVIEWER COMMENTS

Reviewer #1:

This manuscript investigates and analyzes the special behavior of tropical cyclone (TC) stalling at the global scale, presenting the climatology and trends under climate change. The topic is interesting, and the overall flow of the manuscript is clear. However, there are several key issues that prevent me from providing more positive feedback, particularly concerning the reliability of global circulation models (GCMs) in analyzing TC stalling. My detailed comments are provided below, which I hope will be useful to the authors.

Response: Thank you very much for your comments on our research and manuscript. We have addressed all your comments point-by-point below and revised the manuscript accordingly.

Major Comments

1. Reliability of GCMs for TC stalling analysis

My main concern lies in the reliability of using GCMs to analyze TC stalling. Even with a high-resolution model such as AM2.5C360 (25 km) from Princeton, the resolution is still insufficient to fully capture TC structures. Moreover, most stalling events are characterized by abrupt changes in TC trajectories, which GCMs are generally poor at simulating. I strongly recommend that the authors carefully address this issue by presenting sufficient cases from both observations and simulations to justify the validity of using GCMs for stalling studies.

Response: Thank you for your constructive comment. While internal TC variability (such as structural asymmetry) influences TC motion, the occurrence of TC stalling is largely governed by the external large-scale environment. GCMs are capable of effectively capturing the TC response to these external environmental forcings (e.g., large-scale circulation and coexisting TCs). Previous studies have demonstrated that GCMs and reanalysis datasets can reasonably simulate tropical cyclone tracks, with performance improving significantly as spatial resolution increases (from ~200 km to ~25 km)[1–3]. The spatial resolution of the AM2.5C360 model used in this study is finer than that of most existing models in CMIP6 HighResMIP and is comparable to ERA5. Consequently, the model is well-equipped to capture the characteristics of TC tracks.

The two crucial parameters in the definition of TC stalling are the minimum number of track points of the stalled segment, reflecting “stalled time,” and the “minimum enclosing circle radius” containing the corresponding stalled points. To better enhance readers' confidence in the AM2.5C360's simulated TC stalling performance, we evaluated the model's performance under different definitions with multiple combinations of the enclosing circle radius and the stalled time. The sensitivity test included the definition of stalling from previous studies[4–6]. The results indicate that the model consistently reproduces observed TC stalling patterns, regardless of the specific definition applied (Supplementary Fig. 25-34 of the revised manuscript). This consistency confirms that our model's performance is physically grounded rather than being an artifact under specific definitions, demonstrating a

confirmation of the validity of using GCMs for TC stalling studies.

We have added relevant descriptions in the Validation of climate model simulations section of the Methods section in the revised manuscript.

2. Definition of TC stalling

The definition of TC stalling should be clearly and explicitly stated in the manuscript. Although Hall & Kossin (2019) and Zhang et al. (2024) adopted a similar definition, their studies were conducted mainly in the Northern Hemisphere. The authors here report more frequent stalling in the Southern Hemisphere, likely due to slower translation speeds (Kossin et al., 2019). If the definition is based on the relative proportion of slow-moving TCs, then the threshold may need to be adjusted for different basins. Before proceeding with the analysis, the authors should first discuss the robustness and applicability of their definition across regions.

Response: TC stalling is defined as the behavior of a TC remaining within a 200-km radius for more than 48 hours. The two crucial parameters in the definition of TC stalling are the minimum number of track points of the stalled segment, reflecting "stalled time," and the "minimum enclosing circle radius" containing the corresponding stalled points. Refer to the analysis of the definition of stalling in Zhang et al.[4], We calculated the cumulative density function of the minimum enclosing circle radius with 24-hr, 36-hr, 48-hr, 60-hr, and 72-hr stalled time across all the basins during the 1982-2019 period based on observations (Supplementary Fig. 20-24 of the revised manuscript). The results indicate that the number of identified stalled track points increases with increasing stalled time; conversely, the number of stalled track points decreases with increasing minimum enclosing circle radius. We selected 48 hours as the stalled time and 200 km as the minimum enclosing circle radius because this combination is a compromise between sample size and the extremeness of the events. Moreover, similar definition is used in a previous study[4], which facilitates readers' comparative analysis and discussion. We have added relevant descriptions in the TC stalling section of the Methods section in the revised manuscript.

We did not use a relative proportion (percentile-based threshold) definition because a fixed threshold can more obviously show the frequency differences in TC stalling characteristics among basins. In this study, our primary metric is the "stalling ratio," which reflects the probability of a TC undergoing stalling. Using a percentile-based threshold for each basin would complicate the physical interpretation of the stalling ratio and obscure inter-basin variations. In addition, fixed thresholds have also been adopted in defining other extreme TC behaviors, such as Rapid Intensification (RI), which is usually defined as an increase in maximum sustained winds of at least 30 knots (~55 km/h) within 24 hours[7–10]. Even though the frequency of RI varies significantly across basins, this fixed definition remains the widely accepted standard. We apply a similar logic to the definition of stalling to maintain physical consistency.

3. Unclear changes in the Western Pacific (Figure 4)

In Figure 4, the changes in the Western Pacific are difficult to interpret as contiguous or robust.

Instead, the signal appears scattered and statistically weak, with many isolated spots on the map. The authors should carefully check the statistical significance of these results to ensure their robustness.

Response: To address this comment, we have supplemented the results of the significance test in all maps reflecting differences between scenarios (Fig. 3, Fig. 5, and Supplementary Fig. 18 of the revised submission).

4. Novelty relative to previous work

Although this manuscript claims to be a global study of TC stalling, much of the analysis appears similar to Zhang et al. (2024). For instance, Figure 1 largely reproduces results already reported in that study. Rather than repeating known results, the authors should emphasize the differences among basins, highlight potential mechanisms underlying stalling in different regions, and provide a more systematic analysis of the environmental changes that influence stalling globally. This comparative and mechanistic focus would strengthen the novelty of the work.

Response: We acknowledge that we adopted the visualization style of Figure 1 from Zhang et al. (2024), as this format offers high information density and comprehensively illustrates the characteristics and cumulative hazard effects of TC stalling. However, our study presents numerous novel findings that have not been revealed in previous works. For instance, the Eastern Pacific (EP) basin is unique in that the accumulated rainfall from TC stalling is smaller than that from non-stalled storms, which is closely related to sea surface temperatures. Additionally, we found that basins in the Southern Hemisphere are more prone to stalling than those in the Northern Hemisphere. We have added more analyses of TC stalling results from different basins in the Results section. To better highlight the novelty of our work, we have included supplementary explanations and brief discussions regarding specific phenomena in certain basins (Lines 92-96 and Lines 102-106), thereby emphasizing the differences among basins.

Moreveor, as suggested, we have dedicated a significant portion of the manuscript to further exploring the potential dynamical mechanisms of stalling. We constructed basin-specific XGBoost models incorporating both storm characteristics and storm-local environmental variables. We then used the SHapley Additive exPlanations (SHAP) method to interpret these models, analyzing the impact of key dynamical factors on the occurrence of stalling in each basin. We also investigated the primary dynamical drivers of climate warming impact on stalling by calculating changes in stalling-inducing environments. These additional results and analyses further clarify the novelty of our results with respect to the existing literature.

Please refer to the Results section and Discussion section of the revised manuscript for details.

Minor Comments

Line 74: I suggest adopting a threshold based on distance (e.g., kilometers), rather than degrees, as

in Hall & Kossin (2019), to avoid the influence of varying latitudes on the degree measure.

Response: Thank you for your suggestion. We have changed this distance-based threshold from 2 degrees to 200km, and the related calculation results have also undergone slight changes (Lines 81-84, Fig. 3 and Supplementary Fig. 8 of the revised submission), but the main results and conclusions of this study remain largely unchanged.

Line 82: The use of "although" is not appropriate in this sentence; please reconsider the logic.

Response: We have revised this sentence to make it more fluent (Lines 89-92 of the revised manuscript).

Lines 85–86: The number of stalled TC points is less meaningful; the ratio of stalled cases to total TCs is more informative.

Response: Thank you for your suggestion. We have supplemented the statement regarding the TC stall ratio in the Results section and added relevant information in Fig. 1, Supplementary Fig. 1-3, and Supplementary Table 1 of the revised submission.

Lines 215–216: The causes of stalling should be discussed at this point, rather than left implicit.

Response: We have significantly expanded the analyses of the dynamic mechanism of TC stalling in the Results section, but we still put the discussion on the interaction of coexisting TCs in the discussion section.

Kossin, J.P. A global slowdown of tropical-cyclone translation speed. Nature 558, 104–107 (2018). https://doi.org/10.1038/s41586-018-0158-3

Hall, T.M., Kossin, J.P. Hurricane stalling along the North American coast and implications for rainfall. npj Clim Atmos Sci 2, 17 (2019). https://doi.org/10.1038/s41612-019-0074-8

Zhang, L., Cheng, T. F., Lu, M., Xiong, R., & Gan, J. (2023). Tropical cyclone stalling shifts northward and brings increasing flood risks to East Asian Coast. Geophysical Research Letters, 50(10), e2022GL102509.

Reviewer #1 (Remarks on code availability):
The code is comprehensive, but the explanation is limited.

Response: Thank you for appreciating the quality of our effort in reproducing the analyses, consistent with the positive assessment by Reviewer 2 as well.

Reviewer #2:

This manuscript focuses on global distribution of stalling storms and the associated rainfall under climate change scenarios. This is a timely and important piece. While some literature exists, this analysis provides an insightful geographic perspective looking at multiple basins and the variation in behavior. The data and methods are extremely well described, and I was left without unanswered questions. They are written in a reproducible way, which readers will greatly appreciate. For verification of availability, I used the Zenodo site and was able to see the available information. In fact, the information has already been downloaded 23 times as of the writing of this review. I think this manuscript is very close to being ready for publication – with only a few line items mentioned below. Thus, I give minor revisions for this manuscript.

Response: Thank you for your positive assessment. To further enhance the quality of our research, we have supplemented the analysis of the dynamic mechanisms of TC stalling. Overall, we have addressed all the comments point-by-point below.

Line Items

Line 76: add "global" to the sentence before "…global stalled and nonstalled" – so that it is clear that this figure includes all basins together in the depiction of the variables.

Response: We have revised this sentence to make it clearer (Line 84).

Line 88: It is difficult to discern any difference in Figure 1e related to rainfall intensity – are you using Figure 1f to support this statement? It would be helpful to guide the reader to exactly the panel you wish them to consider when describing your results.

Response: Thank you for your suggestion. We have revised this sentence to guide the reader to the precise panel (Line 99).

Line 91: could this be due to generally colder ocean temperatures in the eastern Pacific leading to less rainfall?

Response: Due to the small TC stalling rainfall area and its small overlap area within the EP basin, the local rainfall accumulation induced by TC stalling is also limited. This is an important reason why the accumulated rainfall from TC stalling is smaller than that from non-stalled storms in the EP basin. Notably, TC rainfall area is controlled primarily by its surrounding sea surface temperature (SST) relative to the tropical mean SST (that is, the relative SST)[11]. Since the SST in the EP basin is lower than that in other regions, this lower SST indeed explains why the rainfall patterns of stalling and non-stalling TCs in the EP basin differ from those in other basins. Your suggestion provides valuable insights for further explaining the differences among basins. We have revised this sentence to make it easier to understand (Lines 102-106).

Line 151: In Trepanier et al 2024, we found near-coastal stalls are increasing in the NA. Include if you think it is relevant. We didn't specifically state it is global warming related, but the data support an increase.

Response: Thank you for your suggestion. Our results show that past climate warming has led to a reduction in TC stalling in the land and nearshore areas of the NA basin, which seems different from the results by the Reviewer. We believe that this difference is primarily attributable to differences in the spatial extent of the study areas: our analysis encompasses a broader domain covering all the land and nearshore regions in the NA basin (including the land and offshore areas of regions such as Cuba, the Dominican Republic and Puerto Rico), while their investigation focuses on the coastline of the American continent within the NA basin (see Fig. 2 in Trepanier et al.[5]). To avoid confusion among readers and reconcile the two studies, we have included a brief comparison and discussion of these results in Lines 169-174 of the revised manuscript.

Line 288: Supplementary Figure 8 does not define the basins. I think you mean Figure 1i within the actual manuscript.

Response: Thank you for your suggestion. We have revised this sentence to make it clearer (Line 456 of the revised manuscript).

Reviewer #2 (Remarks on code availability):
It is reproducible and usable. I also comment on this in my main review.

Response: Thank you for the positive assessment.

Reviewer #3:

The authors generate a tropical cyclone stalling global climatology and its projected changes under a warming climate. The study combines observations (IBTrACS tracks and MSWEP reanalysis data) with ~25 km GCM simulations (AM2.5C360) in AMIP and historical/future ensemble configuration using a unique scenario (RCP4.5). This work is subdivided into two future periods (Near Future 2022-2059 and Far Future 2062-2099). The main conclusion is that while the global probability of tropical cyclone stalling decreases, the daily rainfall associated with these TCs increases significantly. This precipitation increases are particularly over land and coastal regions, with increases approaching ~30% in North America at the end of the century.

Overall, the manuscript is generally well-written and provides helpful insight in the topic. However, it bears some important flaws which make it of poor quality for this journal: lack of a clear methodology, statistically significant results, the analysis done to reach the findings, and the findings themselves. Detailed comments are listed to help improve the paper, which requires a very high and substantial major revision before it can be considered for publication in a high-quality journal like Nature Communications.

Response: Thank you for your comments and suggestions. we have addressed all the comments point-by-point below and revised the manuscript accordingly.

General comments:

1. The main findings in this work are not novel enough to be published in this very high-quality journal, given the number of studies concluding an increase in precipitation in TCs. Along these lines, I do not understand the title, as the main findings and analysis are not related to the stalling itself but to the precipitation. On the other hand, the quality of this work would increase notably if it focused on analyzing TC stalling itself, why it stalls and how it could be reduced in the future (i.e., the main dynamical drivers for this reduction), instead of only studying precipitation related to these TCs.

Response: While previous studies showed that total TC-related rainfall is projected to increase under warming scenarios, this general projection does not clarify how the rainfall contributions from stalling versus non-stalling TCs might diverge in the future.

Our results reveal that, in the future, precipitation associated with stalled TCs over North America is projected to increase more substantially than that from non-stalled TCs: highlighting the amplified rainfall potential of stalled TCs in a warming climate has not been found in previous research. This is also a notable and interesting regional difference.

Most previous studies have only focused on the impact of climate warming on TC rainfall, without paying attention to the response of rainfall brought about by the specific movement behavior of TC under climate warming. For instance, Hurricane Ike, a hurricane with rapid movement, and the slow-moving Hurricane Harvey incurred roughly similar economic losses. That said, the factors behind these losses differed: Harvey's damage was driven by heavy rainfall, which caused extensive flooding, whereas Ike's losses stemmed from storm surge-induced flooding and strong wind gusts. The meaning and contribution of the rainfall part of our study lie in clarifying how the rainfall from hurricanes like Harvey and

that from hurricanes like Ike are expected to change under a warming climate.

As suggested, we have dedicated a significant portion of the revised manuscript to further exploring the potential dynamical mechanisms of stalling. We constructed basin-specific XGBoost models incorporating both storm characteristics and storm-local environmental variables. We then used the SHapley Additive exPlanations (SHAP) method to interpret these models, analyzing the impact of key dynamical factors on the occurrence of stalling in each basin. We also investigated the primary dynamical drivers of climate warming impact on stalling by calculating changes in stalling-inducing environments. These additional results and analyses further clarify the novelty of our results with respect to the existing literature.

Please refer to the Results section and Discussion section of the revised manuscript for more details.

Definition of tropical cyclone stalling, rainfall attribution to TC and their robustness. I have some concerns about the tropical cyclone stalling definition because 200-km could be an arbitrary threshold. It could be interesting to do a sensitivity analysis changing the radius (from 150 to 250, for example) and the minimum duration (from 36 to 60 h). In this line, could be interesting testing other radius in the rainfall attribution due to your 500-km fixed threshold may include environmental/monsoonal rainfall. I suggest to test with other radius (400 to 600 km) and rainfall thresholds (0.3 to 1 mm/6 h; 0.5-2 mm/day).

Response: The two crucial parameters in the definition of TC stalling are the minimum number of track points of the stalled segment, reflecting "stalled time," and the "minimum enclosing circle radius" containing the corresponding stalled points (refer to analysis of the definition of stalling in Zhang et al.[4]). We calculated the cumulative density function of the minimum enclosing circle radius with 24-hr, 36-hr, 48-hr, 60-hr, and 72-hr stalled time across all the basins over the 1982-2019 period based on observations (Supplementary Fig. 20-24 of the revised manuscript). The results indicate that the number of identified stalled track points increases with increasing stalled time; conversely, the number of stalled track points decreases with increasing minimum enclosing circle radius. We selected 48 hours as the stalled time and 200 km as the minimum enclosing circle radius because this combination is a compromise consideration between sample size and the extremeness of the events. Moreover, similar definition is used in a previous study[4], which facilitates readers' comparative analysis and discussion. We have supplemented the relevant descriptions in the TC stalling section of the Methods section.

Regarding the rainfall attribution, we acknowledge that using a fixed 500-km threshold may inadvertently include some environmental or monsoonal rainfall. However, this methodology is widely adopted in the majority of TC rainfall studies. While it is a relatively conservative approach, it is computationally efficient. Although sensitivity analyses for precipitation are interesting, they can significantly increase computational costs, especially in light of the other two parameters the Reviewer already asked us to consider. Our analysis of rainfall is mainly to demonstrate the cumulative effect of heavy rain brought by TC stalling, which is an important part of why stalling is highly destructive (Hurricane Harvey is a typical

example). Our research focuses more on stalling itself and its dynamical drivers. The relationship between rainfall and stalling is very interesting, and it is worth conducting a separate research on rainfall induced by stalling in the future.

2. Although the authors used ten ensemble members of the same model, I have some concerns about using a unique future scenario (RCP4.5) and only one model. Including another scenario and several more models would help to capture a wider range of possible responses and improve confidence in the findings. For instance, the use of HighResMIP models could be advised. Or studying this phenomenon in a coupled climate model as we are dealing with TCs.

Response: First, we selected the RCP4.5 scenario as it serves as a reasonable "middle-of-the-road" baseline, reflecting likely outcomes assuming the continuation of current climate policies. Given the substantial computational resources required for these high-resolution simulations, we prioritized the RCP4.5 scenario to provide a representative projection of future conditions within our available capacity.

Second, the use of several members from a single model allows us to examine the role of internal variability, Furthermore, the performance of the AM2.5C360 model has been extensively validated in previous peer-reviewed studies. AM2.5C360 is able to simulate many aspects of the observed TC variability over the past few decades during which reliable observations are available[12–14]. The AM2.5-C360 model generates realistic TC annual cycles and response to El Niño-Southern Oscillation and tropical cloud[13,15].

Third, the spatial resolution of AM2.5C360 is finer than that of the majority of climate models currently available in CMIP6 HighResMIP, enabling it to capture TC track characteristics with high fidelity. Compared to standard CMIP6 models, AM2.5C360 also provides a more comprehensive set of variables necessary for our detailed mechanism and precipitation analyses. To better enhance readers' confidence in the AM2.5C360's simulated TC stalling performance, we evaluated the model's performance under different definitions with multiple combinations of the enclosing circle radius and the stalled time. The sensitivity test included the definition of stalling from previous studies[4–6]. The results indicate that the model consistently reproduces observed TC stalling patterns, regardless of the specific definition applied (Supplementary Fig. 25-34 of the revised manuscript). This consistency confirms that our model's performance is physically grounded rather than being an artifact under specific definitions, confirming the reliability of using AM2.5C360 model for TC stalling studies.

We have added relevant descriptions in the Validation of climate model simulations section and Model and numerical experiments section of the Methods section in the revised manuscript.

Finally, we agree that TC stalling is a complex phenomenon with many aspects remaining to be explored. While investigating the variability of TC stalling across a diverse multi-model ensemble and a wider range of emission scenarios would be valuable, such an extensive comparison is substantial enough to warrant a separate, dedicated study. Future research would also benefit from using even finer spatial resolutions, such as coupled nested regional climate models or convection-permitting models, to further resolve the impacts of

internal TC structure on stalling behavior.

4. Dynamical changes. The manuscript needs an insightful discussion of the main dynamical factors that potentially drive the changes in the TC stalling over each basin. The author includes in the manuscript a dynamical attribution which are discussed from other studies. However, this work needs a dynamical analysis to find what are the changes in their simulations. By including this, the quality of this work would increase notably.

Response: As suggested, we have dedicated a significant portion of the manuscript to further exploring the potential dynamical mechanisms of stalling. We constructed basin-specific XGBoost models incorporating both storm characteristics and storm-local environmental variables. We then used the SHapley Additive exPlanations (SHAP) method to interpret these models, analyzing the impact of key dynamical factors on the occurrence of stalling in each basin. We also investigated the primary dynamical drivers of climate warming impact on stalling by calculating changes in stalling-inducing environments. These additional results and analyses further clarify the novelty of our results with respect to the existing literature.

Please refer to the Results section and Discussion section of the revised manuscript for details.

Reviewer #4 (Remarks to the Author):

I co-reviewed this manuscript with one of the reviewers who provided the listed reports. This is part of the Nature Communications initiative to facilitate training in peer review and to provide appropriate recognition for Early Career Researchers who co-review manuscripts.

Response: We appreciate the transparency regarding the co-review process. We are very supportive of the Nature Communications initiative to facilitate training and provide appropriate recognition for Early Career Researchers. We would like to extend our gratitude to both the primary Reviewer and the Co-Reviewer for their time and the comprehensive feedback provided in the reports, which have been instrumental in improving the quality of our manuscript.

Reference

1. Hodges, K., Cobb, A. & Vidale, P. L. How well are tropical cyclones represented in reanalysis datasets? *Journal of Climate* **30**, 5243–5264 (2017).
2. Roberts, M. J. *et al.* Impact of model resolution on tropical cyclone simulation using the HighResMIP–PRIMAVERA multimodel ensemble. *Journal of Climate* **33**, 2557–2583 (2020).
3. Zhang, G., Murakami, H., Knutson, T. R., Mizuta, R. & Yoshida, K. Tropical cyclone motion in a changing climate. *Science Advances* **6**, eaaz7610 (2020).
4. Zhang, L., Cheng, T. F., Lu, M., Xiong, R. & Gan, J. Tropical cyclone stalling shifts northward and brings increasing flood risks to East Asian Coast. *Geophysical Research Letters* **50**, e2022GL102509 (2023).
5. Hall, T. M. & Kossin, J. P. Hurricane stalling along the North American coast and implications for rainfall. *Npj Climate and Atmospheric Science* **2**, 17 (2019).
6. Trepanier, J. C., Nielsen-Gammon, J., Brown, V. M., Thompson, D. T. & Keim, B. D. Stalling North Atlantic Tropical Cyclones. *Journal of Applied Meteorology and Climatology* **63**, 1409–1426 (2024).
7. Bhatia, K. *et al.* A potential explanation for the global increase in tropical cyclone rapid intensification. *Nature communications* **13**, 6626 (2022).
8. Kaplan, J. & DeMaria, M. Large-scale characteristics of rapidly intensifying tropical cyclones in the North Atlantic basin. *Weather and forecasting* **18**, 1093–1108 (2003).
9. Peng, K., Tian, Y.-X., Fang, J., Liu, Y. & Gu, J.-F. Diversity of tropical cyclones rapid intensification. *Geophysical Research Letters* **51**, e2023GL108006 (2024).
10. Li, Y. *et al.* Recent increases in tropical cyclone rapid intensification events in global offshore regions. *Nature Communications* **14**, 5167 (2023).
11. Lin, Y., Zhao, M. & Zhang, M. Tropical cyclone rainfall area controlled by relative sea surface temperature. *Nature Communications* **6**, 6591 (2015).
12. Yang, W., Hsieh, T.-L. & Vecchi, G. A. Hurricane annual cycle controlled by both seeds and genesis probability. *Proceedings of the National Academy of Sciences* **118**, e2108397118 (2021).
13. Hsieh, T.-L., Yang, W., Vecchi, G. A. & Zhao, M. Model spread in the tropical cyclone frequency and seed propensity index across global warming and ENSO-like perturbations. *Geophysical Research Letters* **49**, e2021GL097157 (2022).
14. Rios, G., Vecchi, G. & Yang, W. Reducing tropical cyclone activity in global climate models by evaporative suppression. *Journal of Geophysical Research: Atmospheres* **130**, e2024JD043302 (2025).
15. Hsieh, T.-L. *et al.* Dependence of tropical cyclone seeds and climate sensitivity on tropical cloud response. *Science advances* **10**, eadi2779 (2024).

REVIEWER COMMENTS

Reviewer #1:

I appreciate the authors' substantial effort in responding carefully to the reviewers' comments and their constructive attitude throughout the revision. However, I remain concerned—consistent with my previous review and broadly aligned with Reviewer 3—that the manuscript may still fall short of the novelty and robustness typically expected for a high-impact journal such as Nature Communications.

Response: Thank you very much for your comments on our manuscript. We have addressed all your comments point-by-point below and revised the manuscript accordingly.

My main concerns are as follows:

1. Model–observation discrepancy and implications for projections.

Based on the newly added Figures S25–S34, the global stalling trends appear to differ substantially between the MMM and the observational estimates. This raises a critical concern about the reliability of the model ensemble for inferring future changes in TC stalling. The manuscript would benefit from a clearer discussion of why these discrepancies arise and how they affect confidence in the projected future trends.

Response: Thank you for your constructive comment. Based on a comprehensive evaluation of both spatial patterns and temporal trends, we find that the model demonstrates a reliable capability to reproduce TC stalling characteristics within the expected margins of uncertainty for climate simulations.

From a **spatial perspective**, the high-resolution ensemble successfully reproduces the observed hotspots of TC stalling and the spatial patterns of stalling-ratio changes across multiple definitions of stalling (i.e., combinations of enclosing-circle radius and stalled time). While some regions in the simulations exhibit locally high stalling ratios (e.g., parts of the southeastern Pacific), these areas contain very few TCs, and thus have negligible influence on basin-scale statistics.

From a **temporal perspective**, the simulated and observed trends are statistically consistent, even when they appear visually different in isolated cases. Both trends are estimated using ordinary least squares, and the simulated and observed trend lines lie close to each other across basins and definitions. Although in a few instances the trend signs differ, the trend magnitudes for the multi-model mean (MMM) and for most ensemble members lie **within the confidence intervals of the observational trends**, indicating no statistically significant discrepancy. These differences reflect acceptable levels of uncertainty inherent in climate modeling rather than a fundamental deficiency in model performance.

We acknowledge several **known limitations**. First, the model underestimates stalling ratios in low-latitude regions of the Southern Hemisphere. In areas where observations show zero stalling, the simulations may produce low, but nonzero, values. These differences arise from internal model variability and ensemble sampling properties. In regions with weak steering flows such as the SPCZ, small differences in initial conditions can shift the position

of narrow convergence zones, affecting low-latitude stalling estimates. Furthermore, instances in which "observations are zero but simulations are non-zero" reflect a well-understood advantage of ensemble simulations: observations represent only a *single realization* of historical climate, whereas ensembles sample a broader distribution that includes rare but physically plausible events not captured in the historical record.

Differences in **temporal trends** are largely attributable to internal climate variability. The observational record is relatively short and sensitive to interannual to decadal oscillations, while the MMM suppresses such noise and primarily reflects externally forced, long-term signals. Thus, some degree of mismatch between observed and simulated trends is expected, and, as noted above, these differences fall within observational confidence intervals. Additionally, year-to-year matching between observed and simulated TC outcomes is not feasible given current model resolution and simplified atmosphere–ocean coupling. Accordingly, our evaluation focuses on climatological behavior, consistent with standard practices in previous studies.

We recognize the inherent uncertainties of climate models. To ensure robustness, we adopted a conservative analytical framework that emphasizes long-term climatological differences and retains only results that pass statistical significance tests across multiple ensemble members. This approach ensures that our projected future changes reflect robust signals rather than noise or model artifacts.

In summary, we acknowledge that discrepancies exist between observations and simulations. However, our analysis demonstrates that:

1. the spatial patterns of TC stalling are well reproduced;
2. simulated and observed trends are statistically consistent across all basins;
3. remaining discrepancies fall within established expectations of internal variability and ensemble sampling; and
4. the revised Methods section ("Validation of climate model simulations," **Lines 677–704**) now explicitly clarifies these points.

Therefore, the model–observation discrepancies do **not** undermine confidence in the projected future trends, and we have strengthened the manuscript accordingly.

2. Limited conceptual novelty relative to prior work.

In my view, the study primarily extends previous regional stalling analyses to a global scale. While this is a useful synthesis, it is not yet clear that the manuscript offers fundamentally new insights into TC behavior or a distinctly innovative study design beyond geographic expansion. Stronger articulation of the unique scientific contribution (e.g., precipitation features of TC stalling before landfall) would be necessary to justify publication in Nature Communications.

Response: Thank you for your comment. We respectfully clarify that this study offers several contributions that go beyond a geographic extension of previous regional analyses. Specifically, the manuscript advances the scientific understanding of TC stalling in **three distinct and substantive ways**, each addressing gaps not previously resolved in the literature.

First, our global analysis reveals previously unrecognized spatial characteristics of TC stalling. Prior studies have focused mainly on the Western North Pacific and North Atlantic,

implicitly suggesting that these basins dominate stalling behavior. In contrast, our results show that the **highest stalling ratios occur in the Southern Hemisphere basins**, a finding not obtainable from regional studies alone. This global perspective identifies new stalling hotspots and provides a unified climatological framework that allows regional results to be placed in a broader physical context.

Second, the study provides a more comprehensive and physically grounded understanding of the mechanisms driving TC stalling. Whereas earlier work has typically relied on case studies or basin-specific attribution analyses, we perform a **global, quantitative assessment** that connects large-scale circulation, storm-local environments, and internal vortex structure into a coherent mechanistic narrative. This includes clarifying the relative importance of steering wind magnitude, directional misalignment, vertical wind shear, and geographic controls across basins. Furthermore, our results show that the contribution of binary TC interactions is **substantially more limited and more basin-dependent** than previously inferred from regional studies, advancing the physical interpretation of stalling dynamics and preventing overgeneralization of mechanisms such as Fujiwhara interactions.

Third, this study represents the **first global evaluation** of TC stalling in a warming climate using high-resolution global climate model simulations. While previous work has used pseudo-global warming frameworks or regional climate models to explore specific events or specific basins, a consistent global assessment of stalling response to climate change has been lacking. Our results demonstrate that changes in the probability of TC exposure to weak steering environments act as the **primary modulator of stalling variability** under warming. We also evaluate, for the first time, the ability of high-resolution GCMs to reproduce stalling behavior, establishing a new modeling benchmark for the field.

Taken together, these three advances demonstrate that this work is not a simple geographic extension, but instead provides **new insights into global stalling behavior**, a **new mechanistic framework**, and **new climate-change projections** unavailable in prior literature. We have revised the Introduction and Discussion to more clearly articulate these points and highlight the novelty of the study.

3. Mechanistic understanding remains insufficient despite added ML analyses.
I acknowledge the authors' additional analyses using XGBoost and SHAP to assess the relative importance of candidate drivers. However, the key limitation in TC stalling research is often the lack of a physically grounded explanation and process-level mechanism, rather than a purely statistical attribution of dominant factors. As currently presented, the machine-learning-based results do not yet translate into a clear physical narrative or testable mechanistic framework (e.g., dynamical pathways linking environmental changes to stalling behavior).

Response: Thank you for this thoughtful and constructive comment. We agree that a statistical ranking of predictors alone is insufficient and that a clear, physically grounded mechanistic explanation is essential for understanding TC stalling. In the revised manuscript, we have therefore substantially strengthened the mechanistic component of the study.

While our original submission included physically motivated interpretations of the SHAP results, we have now gone further by synthesizing these findings into a **coherent,**

process-based framework that explains TC stalling across **three spatial scales**:

1. **Large-scale synoptic environment**, which establishes the background steering flow and its spatial climatology (e.g., the influence of subtropical highs, trade-wind regimes, and low-latitude weak-flow regions).
2. **Storm-local environment**, which exerts direct kinematic forcing on the vortex. This includes the magnitude of the steering wind and the directional misalignment between the storm motion and environmental flow, two of the most influential variables identified by SHAP and physically linked to track deceleration and deflection.
3. **Internal vortex structure**, which modulates the storm's response to external forcing. In particular, strong vertical wind shear can induce vortex tilting and asymmetric upper- and lower-level circulations, resulting in irregular track deviations or stalling.

This three-scale organizational framework integrates the machine-learning results with established TC dynamics (e.g., baroclinic tilt, environmental steering theory, and shear-induced track modulation), providing the process-level explanation the reviewer requested. The revised Discussion (Lines 385–404) now presents this mechanistic narrative explicitly and links the ML-identified drivers to dynamical pathways that are consistent with physical theory and prior literature.

Additionally, we clarify the basin-specific role of binary TC interactions. While previous regional studies have emphasized this mechanism, our global analysis shows that its influence is **limited and heterogeneous**, and in many cases implicitly captured by changes in the local steering environment. This finding further refines the mechanistic interpretation of stalling at the global scale.

Together, these revisions transform the ML-based attribution into a **testable, physically interpretable mechanistic framework**, directly addressing the Reviewer's concern and improving the clarity and scientific value of the manuscript.

Given these concerns, I suggest the authors consider submission to a strong, more specialized venue such as npj Climate and Atmospheric Science or Communications Earth & Environment, where the manuscript may be better aligned with the journal's scope and expectations. In my opinion, the work is likely to meet the standards of those journals.

Response: We thank the Reviewer for their candid assessment and for suggesting alternative venues. We appreciate the concern regarding the novelty and rigor expected for *Nature Communications*, and we have taken this comment seriously in preparing the revised manuscript. After implementing the substantial revisions described in the responses above, including clarifying the model–observation discrepancies, developing a physically grounded mechanistic framework, and articulating the unique contributions of our global analysis, we believe the manuscript now fully satisfies the expectations of the journal.

The revised version offers **three major advances** that, taken together, represent a significant step beyond previous work:

1. **New global insights** into the climatology of TC stalling, including the identification of previously unrecognized stalling hotspots in the Southern Hemisphere and a unified global framework for interpreting regional results.

2. **A new mechanistic explanation**, synthesized across synoptic, storm-local, and vortex-internal scales, which integrates machine-learning findings with established TC dynamics and provides testable physical pathways for stalling behavior.
3. **The first global climate-model–based assessment** of stalling behavior under warming, including the identification of weak-steering exposure as the primary modulator of future changes, thereby providing a new dynamical lens for interpreting stalling under climate change.

These contributions collectively provide **conceptual, mechanistic, and methodological innovations** that we believe are fully aligned with the scope of *Nature Communications*, which highlights studies offering broad relevance, new insights, and significant advances in understanding.

We respectfully hope that, with the strengthened analyses and clarifications in this revised manuscript, both the Reviewer and the Editor will find the paper suitable for publication in *Nature Communications*.

Reviewer #1 (Remarks on code availability):
The code is well prepared.

Response: Thank you for your positive feedback and for recognizing the care we took in ensuring the analyses are fully reproducible.

Reviewer #3:

I have reviewed the revised manuscript and am pleased to report that the authors have successfully addressed all of my previous concerns. It is evident that a significant amount of hard work has gone into improving the quality and clarity of the paper following my review. The scientific content is now robust, and the arguments are well-supported. I am happy to recommend this manuscript for publication. However, before final acceptance, there are several minor spelling, grammar, and syntax errors that need to be corrected.

Please see the list of specific technical corrections below:

1. Page 2, Line 39: The text currently reads: "...one of the costliest No Warming disasters in U.S. history."
Correction: This should be corrected to "natural disasters".

2. Page 2, Line 44: The text reads: "...potentially associated with low-frequency No Warming variability."
Correction: This should be corrected to "natural variability".

3. Page 3, Line 71: "Furthermore, changes in probability of TC stalling in climate warming is mainly affected by..."
Correction: It should read: "...changes... are mainly affected by..."

4. Page 14, Line 388: "Our simulations show that the ratio of TC stalling are declining..."
Correction: It should read: "...the ratio of TC stalling is declining..."

5. Page 3, Line 118: "...there is artifact in the observations..."
Correction: This should read: "...there is an artifact in the observations..."

6. Page 13, Figure 5 Caption (Line 337) & Figure 3 Caption: "...based on the absolute frequency of TC stalling at least the 25th percentile..."
Correction: This phrasing is slightly awkward. It should likely read: "...at least at the 25th percentile..." or "...exceeding the 25th percentile..."

7. Page 14, Line 400: "...the role of this factor on the stalling changes caused by warming is not as much as that of the strength..."
Correction: I suggest changing this to: "...is less significant than that of the strength..."

8. Page 15, Line 413: "...while others that global TC translation speed would increase..."
Correction: It should read: "...while others suggest/project that global TC translation speed..."

9. Page 18 Line 525: "...it is similar to what used in a previous study..."
Correction: This should read: "...similar to what was used in a previous study..."

10. Page 16, Line 439: "...as well as those not in 00:00, 06:00..."
Correction: It should read: "...those not at 00:00, 06:00..."

11. Page 17, Line 488: "...half ensemble members are forced..."
Correction: This should read: "...half of the ensemble members..."
Once these minor issues are resolved, the manuscript will be in excellent shape. Congratulations to the authors on a very interesting study.

Response: We sincerely appreciate the Reviewer's positive assessment of our revised manuscript and their recognition of the extensive efforts invested in strengthening the analysis, clarity, and overall scientific contribution. We are grateful for the Reviewer's recommendation for publication. We have carefully addressed all of the minor spelling, grammar, and syntax corrections listed in the review. We have also thoroughly proofread the entire manuscript to ensure the flow, grammar, and presentation quality now meet the journal's standards. We thank the Reviewer once again for the constructive and supportive feedback, which has helped us substantially improve the clarity and readability of the manuscript.

Reviewer #4 (Remarks to the Author):

I co-reviewed this manuscript with one of the reviewers who provided the listed reports. This is part of the Nature Communications initiative to facilitate training in peer review and to provide appropriate recognition for Early Career Researchers who co-review manuscripts.

Response: We appreciate the transparent explanation of the co-review process. We fully support the Nature Communications initiative to provide training opportunities and formal recognition for Early Career Researchers involved in peer review. We would also like to express our sincere gratitude to both the primary Reviewer and the Co-Reviewer for their time and for the thoughtful, detailed feedback provided in the reports. Their comments have been extremely helpful in improving the quality and clarity of our manuscript.

Reference

1. Fatichi, S. *et al.* Uncertainty partition challenges the predictability of vital details of climate change. *Earth's Future* **4**, 240–251 (2016).
2. Fischer, E. M., Beyerle, U. & Knutti, R. Robust spatially aggregated projections of climate extremes. *Nature Climate Change* **3**, 1033–1038 (2013).
3. Chand, S. S. *et al.* Declining tropical cyclone frequency under global warming. *Nature Climate Change* **12**, 655–661 (2022).
4. Zhang, G., Murakami, H., Knutson, T. R., Mizuta, R. & Yoshida, K. Tropical cyclone motion in a changing climate. *Science Advances* **6**, eaaz7610 (2020).
5. Tao, M. *et al.* Multi-decadal variability controls short-term stratospheric water vapor trends. *Communications Earth & Environment* **4**, 441 (2023).
6. Kossin, J. P. A global slowdown of tropical-cyclone translation speed. *Nature* **558**, 104–107 (2018).
7. Yamaguchi, M., Chan, J. C., Moon, I.-J., Yoshida, K. & Mizuta, R. Global warming changes tropical cyclone translation speed. *Nature Communications* **11**, 47 (2020).
8. Utsumi, N. & Kim, H. Observed influence of anthropogenic climate change on tropical cyclone heavy rainfall. *Nature Climate Change* **12**, 436–440 (2022).
9. Eusebi, R., Vecchi, G. A., Yang, W. & Fueglistaler, S. The Influence of Patterned Warming and CO 2 on North Atlantic Hurricane Frequency. *Journal of Climate* **38**, 5391–5410 (2025).
10. Chan, D., Vecchi, G. A., Yang, W. & Huybers, P. Improved simulation of 19th-and 20th-century North Atlantic hurricane frequency after correcting historical sea surface temperatures. *Science advances* **7**, eabg6931 (2021).
11. Zhang, L., Cheng, T. F., Lu, M., Xiong, R. & Gan, J. Tropical cyclone stalling shifts northward and brings increasing flood risks to East Asian coast. *Geophysical Research Letters* **50**, e2022GL102509 (2023).
12. Trepanier, J. C., Nielsen-Gammon, J., Brown, V. M., Thompson, D. T. & Keim, B. D. Stalling North Atlantic Tropical Cyclones. *Journal of Applied Meteorology and Climatology* **63**, 1409–1426 (2024).
13. Hall, T. M. & Kossin, J. P. Hurricane stalling along the North American coast and implications for rainfall. *Npj Climate and Atmospheric Science* **2**, 17 (2019).
14. Hassanzadeh, P. *et al.* Effects of climate change on the movement of future landfalling Texas tropical cyclones. *Nature communications* **11**, 3319 (2020).
15. Zhang, W., Villarini, G., Vecchi, G. & Smith, J. Urbanization exacerbated the rainfall and flooding caused by hurricane Harvey in Houston. *NATURE* **563**, 384-+ (2018).
16. Dong, K. & Neumann, C. J. On the relative motion of binary tropical cyclones. *Monthly weather review* **111**, 945–953 (1983).
